# Supplementary material for: Monomeric and Oligomeric Decorsins of the Asian Medicinal Leech Hirudinaria manillensis
Source: Int J Mol Sci. 2025 Nov 14;26(22):11017. doi: 10.3390/ijms262211017 (PMC12651989; doi:10.3390/ijms262211017)
Supplement: Supplementary file 1 [file ijms-26-11017-s001.zip › Figure S7.pptx]

## Slide 1
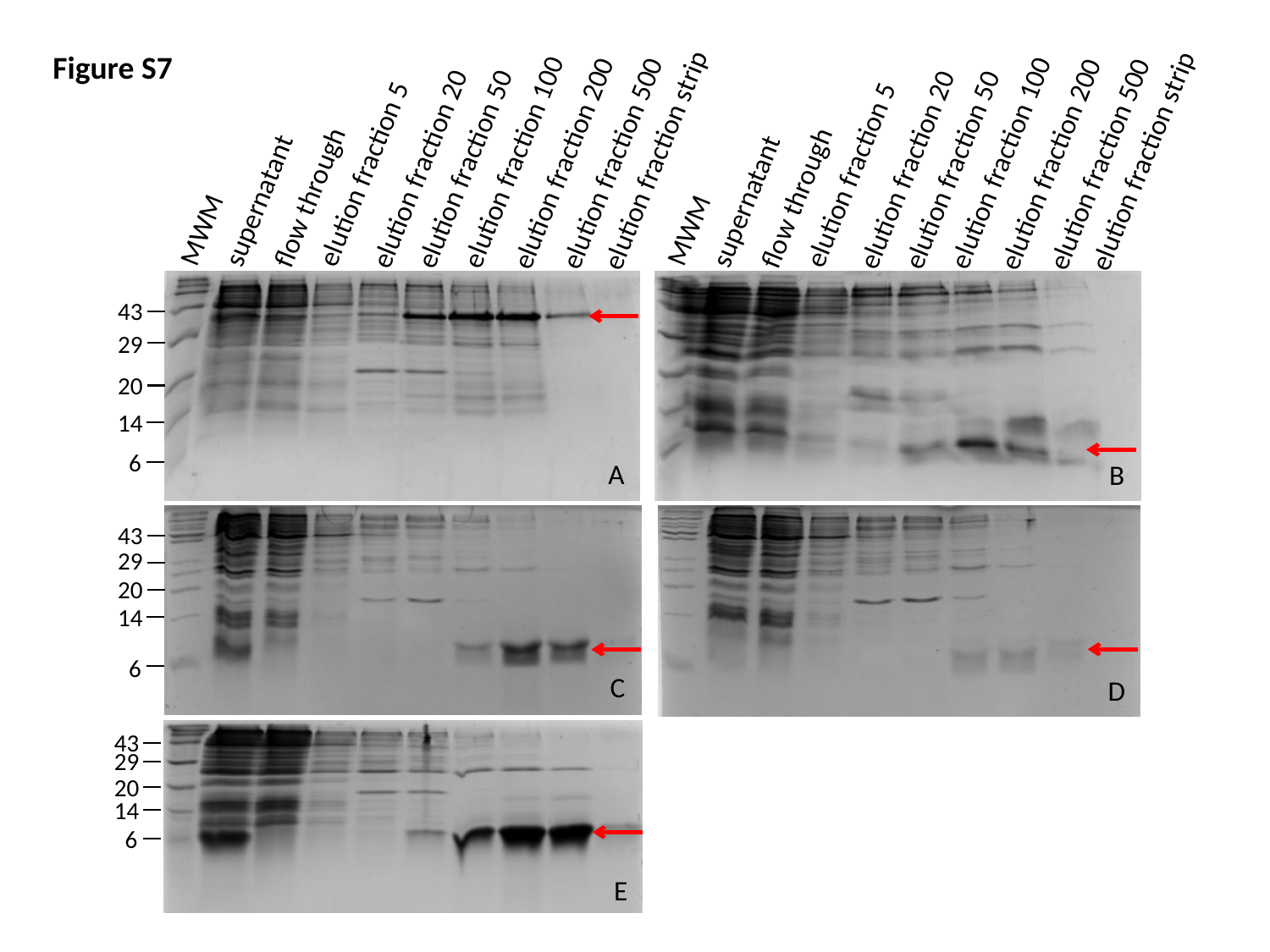

elution fraction strip
elution fraction 100
elution fraction 500
elution fraction 200
elution fraction 20
elution fraction 50
elution fraction 5
flow through
supernatant
MWM
43
29
20
14
 6
elution fraction strip
elution fraction 100
elution fraction 500
elution fraction 200
elution fraction 20
elution fraction 50
elution fraction 5
flow through
supernatant
MWM
Figure S7
A
B
43
29
20
14
 6
C
D
43
29
20
14
 6
E

## Slide 2
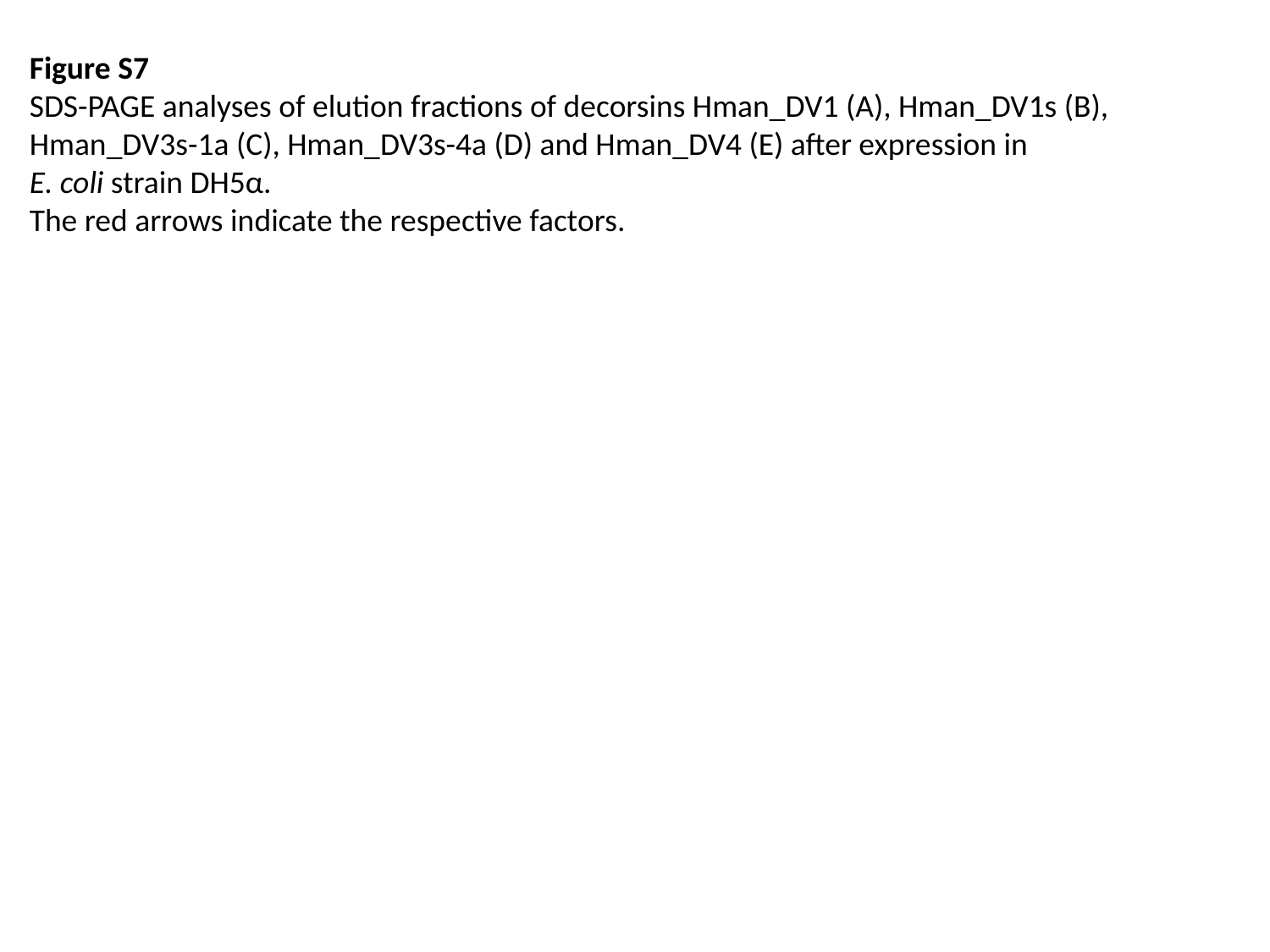

Figure S7
SDS-PAGE analyses of elution fractions of decorsins Hman_DV1 (A), Hman_DV1s (B),
Hman_DV3s-1a (C), Hman_DV3s-4a (D) and Hman_DV4 (E) after expression in
E. coli strain DH5α.
The red arrows indicate the respective factors.
