# Supplementary material for: Monomeric and Oligomeric Decorsins of the Asian Medicinal Leech Hirudinaria manillensis
Source: Int J Mol Sci. 2025 Nov 14;26(22):11017. doi: 10.3390/ijms262211017 (PMC12651989; doi:10.3390/ijms262211017)
Supplement: Supplementary file 1 [file ijms-26-11017-s001.zip › File S1.pdf]

Figure S1. Multiple sequence alignment of putative decorsin gene ranges derived from the genome data of *H. manillensis* provided by Guan et al. (2020), Zheng et al. (2023) and Liu et al. (2023), respectively. The putative decorsin genes are marked in yellow (Hman_DV1), cyan (Hman_DV2), green (Hman_DV3), light grey (Hman_DV4) and red (Hman_DV5). Start and stop codons are marked in bold.

```
                 *        20         *        40         *        60         *        80         *
Guan  : ATGTTAGTTCTGATAATTTGTCTTCTACTGTCCTTTTCATTGGACGTCGATGCAAATAGTTCAAAGAGTGAGTTAATACGATACATTTTG :   90
Zheng : ATGTTAGTTCTGATAATTTGTCTTCTACTGTCATTTTCATTGGACGTCGATGCAAATAGTTCAAAGAGTGAGTTAATACGATTCATTTTG :   90
Liu   : ATGTTAGTTCTGATAATTTGTCTTCTACTGTCCTTTTCATTGGACGTCGATGCAAATAGTTCAAAGAGTGAGTTAATACGATTCATTTTG :   90
        ATGTTAGTTCTGATAATTTGTCTTCTACTGTCcTTTTCATTGGACGTCGATGCAAATAGTTCAAAGAGTGAGTTAATACGATtCATTTTG

               100         *       120         *       140         *       160         *       180
Guan  : AGAAGATATATCAAAACATGTGTATTATATTTTGTAATAGTTTTCAATTGATAATTTAAACGAAGTTCTTGTATAACAGAGAAATTTAGA :  180
Zheng : AGAAGATATATCAAAACATGTGTATTATATTTTGTAATAGTTTTCAATTGATAATTTAAACGAAGTTCTTGTATAACAGAGAAATTTAGA :  180
Liu   : AGATGATATATCAAAACATGTGTATTATATTTTGTAATAGTTTTCAATTGATAATTTAAACGAAGTTCTTGTATAACAGAGAAATTTAGA :  180
        AGAaGATATATCAAAACATGTGTATTATATTTTGTAATAGTTTTCAATTGATAATTTAAACGAAGTTCTTGTATAACAGAGAAATTTAGA

                 *       200         *       220         *       240         *       260         *
Guan  : TAAACATACTTAAATAAAGATTAAAAATAAATACTTATTTTTCAAATAAAATTCTTTCAATATATTAGTTATTGTTATATAATTAAGCAG :  270
Zheng : TAAACATACTTAAATAAAGATTAAAAATAAATACTTATTTTTCAAATAAAGTGCTTTCAATATATTAGTTATTGTTATATAATTAAGCAG :  270
Liu   : TAAACATACTTAAATAAAGATTAAAAATAAATACTTATTTTTCAAATAAAATGCTTTCAATATATTAGTTATTGTTATATAATTAAGCAG :  270
        TAAACATACTTAAATAAAGATTAAAAATAAATACTTATTTTTCAAATAAAaTgCTTTCAATATATTAGTTATTGTTATATAATTAAGCAG

               280         *       300         *       320         *       340         *       360
Guan  : TAGTAGCATTAATATTCAATGTAGGATCACAGCCATGTGAATTAGTGGATAGTGACGAATTTGGATTACCAGTCCCTCCTTGTATTTGTG :  360
Zheng : TAGTATCATTAATATTCAATGTAGGATCACAGCCATGTGGATTAGTGGATAGTGACGAATTTGGATTACCAGTCCCTCCTTGTATTTGTG :  360
Liu   : TAGTAGCATTAATATTCAATGTAGGATCACAGCCATGTGGATTAGTGGATAGTGACGAATTTGGATTACCAGTCCCTCCTTGTATTTGTG :  360
        TAGTAgCATTAATATTCAATGTAGGATCACAGCCATGTGgATTAGTGGATAGTGACGAATTTGGATTACCAGTCCCTCCTTGTATTTGTG

                 *       380         *       400         *       420         *       440         *
Guan  : CTTGGGTTAGTCACTGATCAGCTTTCATAATATCTGCTTATTAAAGATATGCTTATACGCAATTTCTCAATTTGGTATCACTCACGATAT :  450
Zheng : CTTGGGTTAGTCACTGATCAGCTTTGATAATATCTACTTATTAAAGATATGCTTATATGCAATTTCTCAATTTGGTATCACTCACGATAT :  450
Liu   : CTTGGGTTAGTCACTGATCAGCTTTCATAATATCTACTTATTAAAGATATGCTTATATGCAATTTCTCAATTTGGTATCACTCACGATAT :  450
        CTTGGGTTAGTCACTGATCAGCTTTcATAATATCTaCTTATTAAAGATATGCTTATAtGCAATTTCTCAATTTGGTATCACTCACGATAT

               460         *       480         *       500         *       520         *       540
Guan  : TTATGTGAATTGTGAAGCTGATAAATAGTTTAGAAAATACTATGAAGCCATGCCATTATTTATGAAGGATAAGGAGTGTGCACAAGACGA :  540
Zheng : TTATGTAAATTGTGAAGCTGATAAATAGTTTAGAAAATACTATGAAGCCATGCCATTATTTATGAAGGATAAGGAGTGTGCACAAGACGA :  540
Liu   : TTATGTGAATTGTGAAGCTGATAAATAGTTTAGAAAATACTATGAAGCCATGCCATTATTTATGAAGGATAAGGAGTGTGCACAAGACGA :  540
        TTATGTgAATTGTGAAGCTGATAAATAGTTTAGAAAATACTATGAAGCCATGCCATTATTTATGAAGGATAAGGAGTGTGCACAAGACGA

                 *       560         *       580         *       600         *       620         *
Guan  : ATATTGTCAAATTGCTAAAGGAGACGATATCAACTATTGTTTAAAAAGTAGGTTCCGAGTAAATTACGAAATAAAGAATAATCTCATCAT :  630
Zheng : ATATTGTCAAATTGCTAAAGGAGACGATATCAACTATTGTTTAAAAAGTAAGTTCCGAGTAAATTACGAAATAAAGAATAATCTCATCAT :  630
Liu   : ATATTGTCAAATTGCTAAAGGAGACGATATCAACTATTGTTTAAAAAGTAGGTTCCGAGTAAATTACGAAATAAAGAATAATCTCATCAT :  630
        ATATTGTCAAATTGCTAAAGGAGACGATATCAACTATTGTTTAAAAAGTAgGTTCCGAGTAAATTACGAAATAAAGAATAATCTCATCAT

               640         *       660         *       680         *       700         *       720
Guan  : TTAATGTTGCTCTTTTAAAATCAGGAAATTGCGCTGGTGATAAGACTGATGCTCCAATGTGCATTTGTGGAGTAAGTAAAATTGTATCCA :  720
Zheng : TTAATGTTGCTCTTTTAAAATCAGGAAATTGCGCTGGTGATAAGACTGATGCTCCAATGTGCATTTGTGGAGTAAGTAAAATTTTATCCA :  720
Liu   : TTAATGTTGCTCTTTTAAAATCAGGAAATTGCGCTGGTGATAAGACTGATGCTCCAATGTGCATTTGTGGAGTAAGTAAAATTTTATCCA :  720
        TTAATGTTGCTCTTTTAAAATCAGGAAATTGCGCTGGTGATAAGACTGATGCTCCAATGTGCATTTGTGGAGTAAGTAAAATTtTATCCA

                 *       740         *       760         *       780         *       800         *
Guan  : ATATCTATTTTTGTTATTATTATTATTATTATTAT-----------------------------TGTTATAAATGAACTATCTGTTGTAT :  781
Zheng : ATATCTATTTTTGTTATTATTATTATTATTATTATATTATTATTATTATTATTATTATTATTATTGTTATAAATGAACTATCTGTTGTAT :  810
Liu   : ATATCTATTTTTGTTATTATTATTATTAT-----------------------------------TGTTATAAATGAACTATCTGTTGTAT :  775
        ATATCTATTTTTGTTATTATTATTATTATtattat                             TGTTATAAATGAACTATCTGTTGTAT

               820         *       840         *       860         *       880         *       900
Guan  : TTCAGGATAAGCGATGCAGCCATAAGGAAGACTGCAAGAAAGTTGGGGACAAATTACAATGTGTTGAAAGTGAGTTTCTCTTAGTTGCAT :  871
Zheng : TTCAGGATAAGCGATGCAGCCATAAGGAAGACTGCAAGAAAGTTGGGGACAAATTACAATGTGTTGAAAGTGAGTTTCTCTTAGTTGCAT :  900
Liu   : TTCAGGATAAGCGATGCAGCCATAAGGAAGACTGCAAGAAAGTTGGGGACAAATTACAATGTGTTGAAAGTGAGTTTCTCTTAGTTGCAT :  865
        TTCAGGATAAGCGATGCAGCCATAAGGAAGACTGCAAGAAAGTTGGGGACAAATTACAATGTGTTGAAAGTGAGTTTCTCTTAGTTGCAT

                 *       920         *       940         *       960         *       980         *
Guan  : TTGTTGCTACCATGATTTTAAAACATTATATGATTGTATGCACACAAGGATCACTCGATAATTGCATTGAGCTTTGTAATTACTCCTGTA :  961
Zheng : TTGTTGCTACCATGATTTTAAAACATTATATGATTGTATGTACACAAGGATCACTCGATAATTGCATTGAGCTTTGTAATTACTCCTGTA :  990
Liu   : TTGTTGCTACCATGATTTTAAAACATTATATGATTGTATGCACACAAGGATCACTCGATAATTGCATTGAGCTTTGTAATTACTCCTGTA :  955
        TTGTTGCTACCATGATTTTAAAACATTATATGATTGTATGcACACAAGGATCACTCGATAATTGCATTGAGCTTTGTAATTACTCCTGTA

              1000         *      1020         *      1040         *      1060         *      1080
Guan  : TCTTGATCAATCATCGGATGTGAGTGATCTCAACTCAAAGAAAGTTTAAACAAAGCTCAGACATGTCTCTTCACAATGATATAGGCTTTA : 1051
Zheng : TCTTGATCAATCATCGGATGTGAGTGATCTCAACTCAAAGAAAGTTTAAACAAAGCTCAGACATGTCTCTTCACAATGATATAGGCTTTA : 1080
Liu   : CCTTGATCAATCATCGGATGTGAGTGATCTCAACTCAAAGAAAGTTTAAACAAAGCTCAGACATGTCTCTTCACAATGATATAGGCTTTA : 1045
        tCTTGATCAATCATCGGATGTGAGTGATCTCAACTCAAAGAAAGTTTAAACAAAGCTCAGACATGTCTCTTCACAATGATATAGGCTTTA

                 *      1100         *      1120         *      1140         *      1160         *
Guan  : ---TATTTTGCCATAGGATAACAATTTTGTTTAATGAGTGATTTGTAGAAATTCTTATAGATGATTTAAGATGCATTAGGAGCACGTTTT : 1138
Zheng : ---TATTTTGCCATAGGATAACAATTTTGTTTAATGAGTGATTTGTAGAAATTCTTATAGATGATTTAAGATGCATTAGGAGCACGTTTT : 1167
Liu   : CAATATTTTGCCATAGGATAACAATTTTGTTTAATGAGTGATTTGTAGAAATTCTTATAGATGATTTAAGATGCATTAGGAGCACGTTTT : 1135
           TATTTTGCCATAGGATAACAATTTTGTTTAATGAGTGATTTGTAGAAATTCTTATAGATGATTTAAGATGCATTAGGAGCACGTTTT
```

```
            1180         *        1200         *        1220         *        1240         *        1260
Guan  : TCAGAAAAGTGCTAAACAAAGCCACAAAGATTTTAAGATATAACATCAATAATAAAATATTGCAGCAGGTTTCGGTTCCGTTTCTCAGCA : 1228
Zheng : TCAGAAAAGTGCTAAACAAAGCCACAAAGATTTTAAGATATAACATCAATAATAAAATATTGCAGCAGGTTTCGGTTCCGTTTCTCAGCA : 1257
Liu   : TCAGAAAAGTGCTAAACAAAGCCACAAAGATTTTAAGATATAACATCAATAATAAAATATTGCAGCAGGTTTCGGTTCCCTTTCTCAGCA : 1225
        TCAGAAAAGTGCTAAACAAAGCCACAAAGATTTTAAGATATAACATCAATAATAAAATATTGCAGCAGGTTTCGGTTCCgTTTCTCAGCA

                 *        1280         *        1300         *        1320         *        1340         *
Guan  : AATGCATGTTTAAGCTGCAGCCCTTTGATTTCATTAAAATTTTGCTACTAACTAATTTTTAACCAAAATTAAATAAAGGTCTATTTGAAA : 1318
Zheng : AATGCATGTTTAAGCTGCAGCCCTTTGATTTCATTAAAATTTTGCTACTAACTAATTTTTAACCAAAATTAAATAAAAGTCTATTTGAAA : 1347
Liu   : AATGCTTGTTTAAGCTGCAGCCCTTTGATTTCATTAAAATTTTGCTACTAACTAATTTTTAACCAAAATTAAATAAAGGTCTATTTGAAA : 1315
        AATGCaTGTTTAAGCTGCAGCCCTTTGATTTCATTAAAATTTTGCTACTAACTAATTTTTAACCAAAATTAAATAAAgGTCTATTTGAAA

            1360         *        1380         *        1400         *        1420         *        1440
Guan  : GCTTCATGATTTATGAAAGATCCAGAAAGCTATGGGATATGTTAACTCAGCATTTATAAATGTTACTTACTAAATGTAGCTGAATTCTTT : 1408
Zheng : GCTTCAAGATTTATGAAAGATCCATTCAGCTATGGAATATGTTAACTCAGCATTTATAAATGTTACTTACTAAATGTAGCTGAATTCTTT : 1437
Liu   : GCTTCATGATTTATGAAAGATCCAGAAAGCTATGGGATATGTTAACTCAGCATTTATAAATGTTACTTACTAAATGTAGCTGAATTCTTT : 1405
        GCTTCAtGATTTATGAAAGATCCAgaaAGCTATGGgATATGTTAACTCAGCATTTATAAATGTTACTTACTAAATGTAGCTGAATTCTTT

                 *        1460         *        1480         *        1500         *        1520         *
Guan  : TCCATTATATGTTGTGTTAATTGGCTGTTGCTTTTTATCACGTTTTTTTTTT--AATTTTAAAAATAATTTTTTAATAAATTCTTTACGA : 1496
Zheng : TCCATTATATGTTGTGTTAATTGGCTGTTGCTTTTTATCACGTTTTTTTTTTTTAATTTTAAAAATAATTTTTTAATAAATTCTTTACGA : 1527
Liu   : TCCATTATATGTTGTGTTAATTGGCTGTTGCTTTTTATCACGTTTTTTTTT---AATTTTAAAAATAATTTTTTAATAAATTCTTTACGA : 1492
        TCCATTATATGTTGTGTTAATTGGCTGTTGCTTTTTATCACGTTTTTTTTTt  AATTTTAAAAATAATTTTTTAATAAATTCTTTACGA

            1540         *        1560         *        1580         *        1600         *        1620
Guan  : ACTGGTTTACGAATGCTTTGGATGTCACAGGAATACAGTGTTGGTTTAATGCAGAGGTAACATGAGGTAGTTGACTAAAGTTTTAGTAGT : 1586
Zheng : ACTGGTTTACGAATTCTTTGGATGTCACAGGAATACAGTGTTGGTTTAATGCAGAGGTAACATGAGGTAGTTGACTAAAGTTTTAGT--- : 1614
Liu   : ACTGGTTTACGAATTCTTCGGATGTCACAGGAATACAGTGTTGGTTTAATGCAGAGGTAACATGAGGTAGTTGACTAAAGTTTTAGT--- : 1579
        ACTGGTTTACGAATtCTTtGGATGTCACAGGAATACAGTGTTGGTTTAATGCAGAGGTAACATGAGGTAGTTGACTAAAGTTTTAGT

                 *        1640         *        1660         *        1680         *        1700         *
Guan  : ATATGATTTTATTTATCCTGAATGCTAAAATATATCTAAATATATATAAAGATACACTTGTTGTTTATGGACGAAAAGAAGAAATTTCAA : 1676
Zheng : ATATGATTTTATTTATCCTGAGTGCTAAAATATATCTAGATATATATAAAGATACACTTGTTGTTTATGGACGAAAAGAAGAAATTTCAA : 1704
Liu   : ATATGATTTTATTTATCCTGAGTGCTAAAATATATCTAGATATATATAAAGATACACTTGTTGTTTATGGACGAAAAGAAGAAATTTCAA : 1669
        ATATGATTTTATTTATCCTGAgTGCTAAAATATATCTAgATATATATAAAGATACACTTGTTGTTTATGGACGAAAAGAAGAAATTTCAA

            1720         *        1740         *        1760         *        1780         *        1800
Guan  : CGAATTATACATGAATGTACAAAAGTGACATTAACGTTGATATTAATATTAAATACAGAACCGAAGCTTTGTTCAAAAGTGAAGAAAGAT : 1766
Zheng : CGAATTATACATGAATGTACAAAAGTGACATTAACGTTGATATTAATATTAAATACAGAACCGAAGCTTTGTTCAAAAGTGAAGAAAGAT : 1794
Liu   : CGAATTATACATGAATGTACAAAAGTGACATTAACGTTGATATTAATATTAAATACAGAACCGAAGCTTTGTTCAAAAGTGAAGAAAGAT : 1759
        CGAATTATACATGAATGTACAAAAGTGACATTAACGTTGATATTAATATTAAATACAGAACCGAAGCTTTGTTCAAAAGTGAAGAAAGAT

                 *        1820         *        1840         *        1860         *        1880         *
Guan  : GAATTTGGAATACCGATTCGTCCCTGTGCTTGTCAATGGGTTAGTATTGATCAATTACTGGTAATTTATCTACTCACTAAAAAAACGAGCT : 1856
Zheng : GAATTTGGAATACCGATTCGTCCCTGTGCTTGTCAATGGGTTAGTATCGATCAATTACTGGTAATTTATCTACTCACTAAAAAAACGAGCT : 1884
Liu   : GAATTTGGAATACCGATTCGTCCCTGTGCTTGTCAATGGGTTAGTATCGATCAATTACTGGTAATTTATCTACTCACTAAAAAAACGAGCT : 1849
        GAATTTGGAATACCGATTCGTCCCTGTGCTTGTCAATGGGTTAGTATcGATCAATTACTGGTAATTTATCTACTCACTAAAAAAACGAGCT

            1900         *        1920         *        1940         *        1960         *        1980
Guan  : TGTATGTAATTTCTCAATTACTCATGACATTTATGTCACTGTAAAGTTTTAATTAATTAAAATGAGGCGCTTCATTCTTTATACGCTCAT : 1946
Zheng : TGTATGTAATTTCTCAATTACTCATGACATTTATGTCACTGTAAAGTTTTAATTAATTAAAATGAGGCGCTTCATTCTTTATACGCTCAT : 1974
Liu   : TGTATGTAATTTCTCAATTACTCATGACATTTATGTCACTGTAAAGTTTTAATTAATTAAAATGAGGCGCTTCATTCTTTATACGCTCAT : 1939
        TGTATGTAATTTCTCAATTACTCATGACATTTATGTCACTGTAAAGTTTTAATTAATTAAAATGAGGCGCTTCATTCTTTATACGCTCAT

                 *        2000         *        2020         *        2040         *        2060         *
Guan  : CCATCCATTCATTCATCCACGCATTCGTCCATCTTATCCTTCCTTCCATCTATAAATAAATCCTACCACCCATCCCCATCCAACTTAACC : 2036
Zheng : CCATCCATTCATTCATCCACGCATTCGTCCATCTTATCCTTCCTTCCATCTATAAATAAATCCTACCACCCATCCCCCAGCGACTCAACC : 2064
Liu   : CCATCCATTCATTCATCCACGCATTCGTCCATCTTATCCTTCCTTCCATCTATAAATAAATCCTACCACCCATCCCCATCCAACTCAACC : 2029
        CCATCCATTCATTCATCCACGCATTCGTCCATCTTATCCTTCCTTCCATCTATAAATAAATCCTACCACCCATCCCCatcCaACTcAACC

            2080         *        2100         *        2120         *        2140         *        2160
Guan  : AATTTACA-CTATGATTCAGAAAACATAGTGGTGATTTCGTACCATTATTTATCAAGGGTGTTGAGTGTGAAGAGCACGAGTATTGTAAT : 2125
Zheng : AATTTAAAACAATGATTTAGAAAACATAGTGGTGAATTCGTACCATTATTTATCAAGGGTGTAGAGTGTGAAGAGCACGAGTATTGTAAT : 2154
Liu   : AATTTACA-CTATGATTCAGAAAACATGGTGGTGATTTCGTACCATTATTTATCAAGGGTGTTGAGTGTGAAGAGCACGAGTATTGTAAT : 2118
        AATTTAcA CtATGATTcAGAAAACATaGTGGTGAtTTCGTACCATTATTTATCAAGGGTGTtGAGTGTGAAGAGCACGAGTATTGTAAT

                 *        2180         *        2200         *        2220         *        2240         *
Guan  : ATTACTGAAGGATACTCCATCGGCACTTGTGAACGATGTAAGTTCAGAGAAAATTGCGAAATAAACAATAATATCATAATAGG------- : 2208
Zheng : ATTACTAAAGGATACTCCATCGGCACTTGTGAACAATGTAAGTTCAGAGAAAATTTAGAAATAAAAAATAATATCATAATTGGTTAATTT : 2244
Liu   : ATTACTAAAGGATACTCCATCGGCACTTGTGAACGATGTAAGTTTCGAGAAAATTTAGAAATAAAAAATAATATCATAATTGGTTAATTT : 2208
        ATTACTaAAGGATACTCCATCGGCACTTGTGAACgATGTAAGTTcaGAGAAAATTtaGAAATAAAaAATAATATCATAATtGGttaattt

            2260         *        2280         *        2300         *        2320         *        2340
Guan  : -ATGATTAATTTTGACATTTATTTTCTATTAATATTTAACATAATAAG-------------GTGAATATTTGAATTAATAGATTTATATC : 2284
Zheng : GATGATTAATTTTGACATTTAGTTTCTATTAATATTTAATATAATAAG-------------GTGAATATTTAAATTATTAGAATTATATC : 2321
Liu   : GATGATTAATTTTGACATTTAGTTTCTATTAATATTTAATATAATAAGTATAATAAATATAATAAATATTTGAATTAATAGAATTATATC : 2298
        gATGATTAATTTTGACATTTAgTTTCTATTAATATTTAAtATAATAAG             gTgAATATTTgAATTAaTAGAaTTATATC

                 *        2360         *        2380         *        2400         *        2420         *
Guan  : CTAAAAAAATCGAGAAATAAGTTAAATTTAAATGTCAGATTAAACTGGTTAACATTCAAACAAAAACGTTTTAAAATCAGTGTATTGTGC : 2374
Zheng : CTAAAA--ATAGAGAAATAAGTTAAATTTAAATATCAGATTAAACTGGTTAACATTAAAACATAAACGTTTTAAAATCAGTGTATTGTGC : 2409
Liu   : CTAAAA--ATAGAGAACTAAGTTAAATTTAAATGTCAGATTAAACTGGTTAACATTAAAATAAAAACGTTTCAAAATCAGTGTATTGTGC : 2386
        CTAAAA  ATaGAGAAaTAAGTTAAATTTAAATgTCAGATTAAACTGGTTAACATTaAAAcAaAAACGTTTtAAAATCAGTGTATTGTGC
```

```
               2440         *        2460         *        2480         *        2500         *        2520
Guan   : GGGTGAACCGGCTGTAGATTCTCCAGTGTGCATATGTAAAGTCAGTAACATTTTATCTAATGCTAGTAATTATCAATTATTAATAATAAT : 2464
Zheng  : GGGTAACCCGGCTGAAGATTCTCCAGTGTGCTTATGTAAAGTCAGTAACATTTTATCTAATGCTAATAATTATCAATTATTAATAATATT : 2499
Liu    : GGGTAACCCGGCTGAAGATTCTCCAGTGTGCTTATGTAAAGTCAGTAACATTTTATCTAATGCTAATAATTATCAATTATTAATAATATT : 2476
         GGGTaAcCCGGCTGaAGATTCTCCAGTGTGCtTATGTAAAGTCAGTAACATTTTATCTAATGCTAaTAATTATCAATTATTAATAATAtT

                 *        2540         *        2560         *        2580         *        2600         *
Guan   : AAGGATAATTATGAGGAGAAAATGTAGGAATGTCATTTGAATTTCAGGATAAGCGATGCGACCGTAATCAATACTGTAGGAACGTTGATG : 2554
Zheng  : AAAGATAATTATGAGGAGAAAATGTAGGAATGTCATTTGTCTTTCAGGATAAGCGATGCGACCGTAATCAATACTGTAGGAACGTTGATG : 2589
Liu    : AAAGATAATTATGAGGAGAAAATGTAGGAATGTCATTTGTCTTTCAGGATAAGCGATGCGACCGTAATCAATACTGTAGGAACGTTGATG : 2566
         AAaGATAATTATGAGGAGAAAATGTAGGAATGTCATTTGtcTTTCAGGATAAGCGATGCGACCGTAATCAATACTGTAGGAACGTTGATG

               2620         *        2640         *        2660         *        2680         *        2700
Guan   : GCAAGTTGGAATGTGATGAAAGTGAGTTTCTCTTACCTGTTTTTTTT-ATTGTGAGACAAATTATTT-AAAGCTGTTAATATCATGATTT : 2642
Zheng  : GCAAGTTGGAATGTGATGAAAGTGAGTTTCTCTTACCTGTTTTTTTTTATTGTGAGACAAGTTATTTGAAAGTTGCTAATATCAGGATTT : 2679
Liu    : GCAAGTTGGAATGTGATGAAAGTGAGTTTCTCTTACCTGTTTTTTTTTATTGTGAGACAAGTTATTTGAAAGTTGCTAATATCAGGATTT : 2656
         GCAAGTTGGAATGTGATGAAAGTGAGTTTCTCTTACCTGTTTTTTTTtATTGTGAGACAAgTTATTTgAAAGtTGcTAATATCAgGATTT

                 *        2720         *        2740         *        2760         *        2780         *
Guan   : AAAAATAT-TCTGTGTGTAATATTGAAGTAATAATAATAATAATTTAAATATATAATATGTGCATGTACCACTATTACTACT-----TAG : 2726
Zheng  : AAAAAAAAATCTACATGTAATATTG---------TAATAATAATATAAATATATATTATGTATATGTATTATTACTACTACTACTTCTAT : 2760
Liu    : AAAAAAAAATCTACATGTAATATTG---------TAATAATAATATAAATATATATTATGTGTATGTATTATTACTACTACTACTTCTAT : 2737
         AAAAAaAaaTCTacaTGTAATATTG         TAATAATAATaTAAATATATAtTATGTgtATGTAttAtTAcTACTACTacttcTAt

               2800         *        2820         *        2840         *        2860         *        2880
Guan   : CAATAATAATAATAATAATAATGTATTT-----CATCAAACTGAGCCTTAACAGAAGGTTATGACTAATGCTCTAATATTTTATGTAAGA : 2811
Zheng  : TAGTGGTAATAATAATAATAATGTATTTGAAATCATCAAACGGAGTCTTAAGAGAAGGTAATAACTAATGCTCTAATATTTTATGTAAAA : 2850
Liu    : TAGTGGTAATAATAATAATAATGTATTTGAAATCATCAAACGGAGTCTTAAGAGAAGGTAATAACTAATGCTCTAATATTTTATGTAAAA : 2827
         tAgTggTAATAATAATAATAATGTATTTgaaatCATCAAACgGAGtCTTAAgAGAAGGTaATaACTAATGCTCTAATATTTTATGTAAaA

                 *        2900         *        2920         *        2940         *        2960         *
Guan   : TTGATTGCACATTGCTCTTAGGTTACTGATTTAGTTAATTTGATTTATTATCAAATGAGGGACATTTTTTGCCAGCTCTGCCGATTATCA : 2901
Zheng  : TTAAGTGCGTATTGCTCTTTGGTTACTAATTTAA---------------TAACAAATGAGCTAATATTTTTGCCAGCTATGCCTATTATCA : 2926
Liu    : TTAAGTGCGTATTGCTCTTTGGTTACTAATTTAA---------------TAACAAATGAGCTAATATTTTTGCCAGCTATGCCTATTATCA : 2903
         TTaAgTGCgtATTGCTCTTtGGTTACTaATTTAa               TAaCAAATGAGctAataTTTTTGCCAGCTaTGCCtATTATCA

               2980         *        3000         *        3020         *        3040         *        3060
Guan   : GTTAATCCGCTGTTAAGTATTATCACGTTATTATTAAATACAGAACCGAAGAGTTGCGAAATTATGGAGAAGGACAAACTAGGTCGACCT : 2991
Zheng  : GTTAATCCGCTGTTAAGTATTATTATGTTATTATTAAATACAGAACCGAAGAGTTGCGAAAATATGGAGAAGGACAAACTAGGTCGACCT : 3016
Liu    : GTTAATCCGCTGTTAAGTATTATTACGTTATTATTAAATACAGAACCGAAGAGTTGCGAAAATATGGAGAAGGACAAACTAGGTCGACCT : 2993
         GTTAATCCGCTGTTAAGTATTATtAcGTTATTATTAAATACAGAACCGAAGAGTTGCGAAAaTATGGAGAAGGACAAACTAGGTCGACCT

                 *        3080         *        3100         *        3120         *        3140         *
Guan   : CTTGGGCCTTGCGTTTGTGGAACTGTTAGTCGTCGATAAATTTTGGTTATCGGAATACTTTTTAAAATCGTTCTTAAATTTAATTTATGA : 3081
Zheng  : ATTGGGCCTTGCGTTTGTGGAACTGTTAGTCGTCGATAAATTTTGGTTATCGGAATACTTTTTAAAATCGTATTCAAATTCAATTTATCA : 3106
Liu    : ATTGGGCCTTGCGTTTGTGGAACTGTTAGTCGTCGATAAATTTTGGTTATCGGAATACTTTTTAAAATCGTATTCAAATTCAATTTATGA : 3083
         aTTGGGCCTTGCGTTTGTGGAACTGTTAGTCGTCGATAAATTTTGGTTATCGGAATACTTTTTAAAATCGTatTcAAATTcAATTTATgA

               3160         *        3180         *        3200         *        3220         *        3240
Guan   : ATTTATTATACATATTTTAAACATTGTAAAGTTTAGAAGACATTTATGAAACCACAATCATTATTTACCCAGGATCGGGTGTGTGAAAGG : 3171
Zheng  : ATTTATTATAAAAATTTTAAACATTGTAAAGTTTGGAAGACATTTATGAAGTCACAATCATTATTTACCAAGGGTCGGGTGTGTGAAAGG : 3196
Liu    : ATTTATTATAAAAATTTTAAACATTGTAAAGTTTGGAAGACATTTATGAAGTCACAATCATTATTTACCAAGGGTCGGGTGTGTGAAAGG : 3173
         ATTTATTATAaAaATTTTAAACATTGTAAAGTTTgGAAGACATTTATGAAgtCACAATCATTATTTACCaAGGgTCGGGTGTGTGAAAGG

                 *        3260         *        3280         *        3300         *        3320         *
Guan   : CATGAATACTGTGATATTACACTTGAAGATAACACCAACAATTGCTTACCAAGTACGTTTCGTTTAGATTATGAAATAAACAATTATATC : 3261
Zheng  : CACGAATACTGTGATATTACACTTAAAGATGCCACCGACAATTGCTTACCAAGTACGTTTCGTTTAGATTATGAAATAAACAATTATACC : 3286
Liu    : CACGAATACTGTGATATTACACTTAAAGATGCCACCGACAATTGCTTACCAAGTACGTTTCGTTTAGATTATGAAATAAACAATTATACC : 3263
         CAcGAATACTGTGATATTACACTTaAAGATgcCACCgACAATTGCTTACCAAGTACGTTTCGTTTAGATTATGAAATAAACAATTATAcC

               3340         *        3360         *        3380         *        3400         *        3420
Guan   : AAGATTTAGAGATTAATTTTCGCTATCGATTTTTATTAATTCTTTGATTGTGCCCAGAGTACAATCCGTGCGAACAAGGGTCAATTGTAG : 3351
Zheng  : AAGATTTAGGGATTAATATTCACA-TTGATTTTTACTAATTCTTTGATTGTACACAGAGTACAATCCGTGCAAACAAGGGTCATTTGTAG : 3375
Liu    : AAGATTTAGGGATTAATTTTCACC-TTGATTTTTACTAATTCTTTGATTGTACACAGAGTACAATCCGTGC-AACAAGGGTCATTTGTAG : 3351
         AAGATTTAGgGATTAATtTTCaC  TtGATTTTTAcTAATTCTTTGATTGTaCaCAGAGTACAATCCGTGC AACAAGGGTCAtTTGTAG

                 *        3440         *        3460         *        3480         *        3500         *
Guan   : AGAAGAAATGTTTATGTGGTGTTAGTAACTATTACTGACAAACATTCTTCAATATTTCCAAC-AAAAGATAATTATTACATTAAAGTACC : 3440
Zheng  : AGAAGAAATGTTCATGTGGTGTTAGTAACTATTACTGACAAACATTCTTCAGTATTTCCAACTAAAAGATTATTATTACATTAAAATACC : 3465
Liu    : AGAAGAAATGTTCATGTGGTGTTAGTAACTATTACTGACAAACATTCTTCAGTATTTCCAACTAAAAGATTATTATTACATTAAAATACC : 3441
         AGAAGAAATGTTcATGTGGTGTTAGTAACTATTACTGACAAACATTCTTCAgTATTTCCAACtAAAAGATtATTATTACATTAAAaTACC

               3520         *        3540         *        3560         *        3580         *        3600
Guan   : TAAAAATAACATTAAATAAAGTTTGTCATTTGTTACATATCTAGAAAAAGTCTTGCAGCAAAGGGAAGAAATGCATATTGATTGACGGCG : 3530
Zheng  : TAAATATAACATTAAATAAAGTTTGTCATTTGTTACATATCTAGAAAAAGTCTTGCAGCAAAGGGAAGAAATGCGTATTGATTGACGGCG : 3555
Liu    : TAAATATAACATTAAATAAAGTTTGTCATTTGTTACATATCTAGAAAAAGTCTTGCAGCAAAGGGAAGAAATGCGTATTGATTGACGGCG : 3531
         TAAAtATAACATTAAATAAAGTTTGTCATTTGTTACATATCTAGAAAAAGTCTTGCAGCAAAGGGAAGAAATGCgTATTGATTGACGGCG

                 *        3620         *        3640         *        3660         *        3680         *
Guan   : AAGCAAATTGCACAAGGTTGTAAACGCATCGTCGTGATGCTATTAAAATAATTTAAGTTTTGGAATAATTATTGTTATCAATAATTCTAT : 3620
Zheng  : AAGCAAATTGCATAAGGTTGTAAACGCATCGTCGTGATGCTATTAAAATAATTTAAGTTTTGGAATAATTATTGTTATCAATAATTCTAT : 3645
Liu    : AAGCAAATTGCATAAGGTTGTAAACGCATCGTCGTGATGCTATTAAAATAATTTAAGTTTTGGAATAATTATTGTTATCAATAATTCTAT : 3621
         AAGCAAATTGCAtAAGGTTGTAAACGCATCGTCGTGATGCTATTAAAATAATTTAAGTTTTGGAATAATTATTGTTATCAATAATTCTAT
```

```
              3700         *        3720         *        3740         *        3760         *        3780
Guan  : CAATATTAGTAGATTAATATTATTAATAATTCTATTATTTTTAGTTCCCAACAAGAAGATGTTCAACATACCGAATAAGGTGGACCACCG : 3710
Zheng : CAATATTAGTAGATTAATATTATTAATAATTCTATTATTTTTAGTACCCAACAAGAAGATGTTCAACATACCGAATAAGGCGGACCACCG : 3735
Liu   : CAATATTAGTAGATTAATATTATTAATAATTCTATTATTTTTAGTACCCAACAAGAAGATGTTCAACATACCGAATAAGGCGGACCACCG : 3711
        CAATATTAGTAGATTAATATTATTAATAATTCTATTATTTTTAGTaCCCAACAAGAAGATGTTCAACATACCGAATAAGGcGGACCACCG

                 *        3800         *        3820         *        3840         *        3860         *
Guan  : AACCTGATAAAACATCTCATTTTATGAACATTTCATAACTTGTTGCTGTTCTGAATTTAACTAACCATACGTTAATAAATTACTTAATAG : 3800
Zheng : AACCTGATAAAACATCTCATTTTATGAACATTTCATAACTTGTTGCTGTTCTGAATTTAACTAACCATACGTTAATAAATTACTTAATAG : 3825
Liu   : AAC-TGATGAAACATCTCATTTTATGAACATTTCATAACTTGAAGCTGTTCTGAATTTAACTAACCATACGTTAATAAATTACTTAATAG : 3800
        AACcTGATaAAACATCTCATTTTATGAACATTTCATAACTTGttGCTGTTCTGAATTTAACTAACCATACGTTAATAAATTACTTAATAG

              3880         *        3900         *        3920         *        3940         *        3960
Guan  : AAATAAA----------AATCAAACATCGTTTCTAATAACACTTAAGTTTCACTTTCAAGTTTGTACGATTATCCGAGAATTGGATGGGC : 3880
Zheng : AAATAAACAAACCATCTAATCAAACATCGTTTCTAATAACACTTAAGTTTCACTTTCAAGTTTGTACGATTATCCGAGAATTGGATGGGC : 3915
Liu   : AAATAAACAAACCATCTAATCAAACATCGTTTCTAATAACACTTAAGTTTCACTTTCAAGTTTGTACGATTATCCGAGAATTGGATGGGC : 3890
        AAATAAAcaaaccatctAATCAAACATCGTTTCTAATAACACTTAAGTTTCACTTTCAAGTTTGTACGATTATCCGAGAATTGGATGGGC

                 *        3980         *        4000         *        4020         *        4040         *
Guan  : ACAAATTGGGGACAATTTTCTTTAGGAATATTGTAAAAGATATTTTTAGGTGAGATTGCATATAGGAAGAGACATGGGTTCTTTACATGA : 3970
Zheng : ACAAATTGGGGACAATTTTCTTTAGGAATATTGTAAAAGATATTTTTAGGTGAGATTGCATATAGGAAGAGACATGGGTTCTTTACATGA : 4005
Liu   : ACAAATTGGGGACAATTTTCTTTAGGAATATTGTAAAAGATATTTTTAGGTGAGATTGCATATAGGAAGAGACATGGGTTTTTTACATGA : 3980
        ACAAATTGGGGACAATTTTCTTTAGGAATATTGTAAAAGATATTTTTAGGTGAGATTGCATATAGGAAGAGACATGGGTTcTTTACATGA

              4060         *        4080         *        4100         *        4120         *        4140
Guan  : CAATCAACTTTTATTATTGGAAATTATTATTAATAATAAAGTATTTTCTGGTTTGTTAATAATATTAAGTAATATTCTCAGGCTTGTAAA : 4060
Zheng : CAATCAACTTTTATTATTGGAAATTATTATTAATAATAAAGTATTTTCTGGTTTGTTAATAATATTAAGTAATATTCTCAGGCTTGTAAA : 4095
Liu   : CAATCAACTTTTATTATTGGAAATTATTATTAATAATAAAGTATTTTCTGGTTTGTTAATAATATTAAGTAATATTCTCAGGCTTGTAAA : 4070
        CAATCAACTTTTATTATTGGAAATTATTATTAATAATAAAGTATTTTCTGGTTTGTTAATAATATTAAGTAATATTCTCAGGCTTGTAAA

                 *        4160         *        4180         *        4200         *        4220         *
Guan  : ATTAAACGAACCTAATATTTCGGTGACAGGCCAAGGGAAAGGTTAAGGATTCCCTAGTGTTTCACAAAAAAAAATTCGTAATACTAAATTG : 4150
Zheng : ATTAAACGAACCTAATATTTCGGTGACAGGCCAAGGGAAAGGTCTAGCATTCCCTAGAGTTTCAC--AAAAAAATTCGTAATACTAAATTG : 4183
Liu   : ATTAAACGAACCTAAAATTTCGGTGACAGGCCAAGGGAAAGGTTTAGGATTCCCTAGAGTTTCACCAAAAAAAATTCGTAATACTAAATTG : 4160
        ATTAAACGAACCTAAtATTTCGGTGACAGGCCAAGGGAAAGGTttAGgATTCCCTAGaGTTTCAC aAAAAAAATTCGTAATACTAAATTG

              4240         *        4260         *        4280         *        4300         *        4320
Guan  : GAATTCTAAAGTTATTAAGACATCATTTTACGATTGATCCTTTCGTATCTTAGAAAAAATGGGAAGACACTCATAATCGATAGCCATTTAT : 4240
Zheng : GAATTCTAAAGTTATTAAGAAATCATTTTACGATTGATCCTTTCGTATCTTAGAAAAAATGGGAAGACACTCATAATTGATAGCCATTTAT : 4273
Liu   : GAATTCTAAAGTTATTAAGAAATCATTTTACGATTGATCCTTTCGTATCTTAGAAAAAATGGGAAGACACTCATAATCGATAGCCATTTAT : 4250
        GAATTCTAAAGTTATTAAGAaATCATTTTACGATTGATCCTTTCGTATCTTAGAAAAAATGGGAAGACACTCATAATcGATAGCCATTTAT

                 *        4340         *        4360         *        4380         *        4400         *
Guan  : ATAGCACCCCCTGGTACCGTGATAAGAAAATTTCACTAGTTCTCATTTTCTCGTCTTTAATCTGAAAATAAAAATAATTGATGTAGTGGA : 4330
Zheng : ATAGCACCCCCTGGTACCGTGATAAGAAAATTTCACTAGTTCTCATTTTCTCGTCTTTAATCTGAAAATAAAAATAATTGATATAGTGGA : 4363
Liu   : ATAGCACCCCCTGGTACCGTGATAAGAAAATTTCACTAGTTCTCATTTTCTCGTCTTTAATCTGAAAATAAAAATAATTGATGTAGTGGA : 4340
        ATAGCACCCCCTGGTACCGTGATAAGAAAATTTCACTAGTTCTCATTTTCTCGTCTTTAATCTGAAAATAAAAATAATTGATgTAGTGGA

              4420         *        4440         *        4460         *        4480         *        4500
Guan  : TAGATTCTGAATTAATATTTGTATTTAGCTGATTTTTAGTTTTATGCATTTTAATCAGATTAAAATAATTTAGAAAAATAACTTGATATT : 4420
Zheng : TAGATTCTGAATTAATATTTGTATTTAGCTGATTTTTAGTTTTATGCATTTTAATCAGATTAAAATAATTTAGAAAAATAACTTGATATT : 4453
Liu   : TAGATTCTGAATTAATATTTGTATTTAGCTGATTTTTAGTTTTATGCATTTTAATCAGATTAAAATAATTTAGAAAAATAACTTGATATT : 4430
        TAGATTCTGAATTAATATTTGTATTTAGCTGATTTTTAGTTTTATGCATTTTAATCAGATTAAAATAATTTAGAAAAATAACTTGATATT

                 *        4520         *        4540         *        4560         *        4580         *
Guan  : TATTATTTTACTTTTTGAATTAAACGAAACGCTTTACTTTTGCTAAAACAGCATTTACAGTATTTATTTGCTTTGTAAGTCTTGCAACTC : 4510
Zheng : TATTATTTTACTTTTTGAATTAAACGAAACTCCTTACTTTTGCTAAAACAGCATTTACAGTATTTATTTGCTTTGTAAGTCTTGCAACTC : 4543
Liu   : TATTATTTTACTTTTTGAATTAAACGAAACTCCTTACTTTTGCTAAAACAGCATTTACAGTATTTATTTGCTTTGTAAGTCTTGCAACTC : 4520
        TATTATTTTACTTTTTGAATTAAACGAAACtCcTTACTTTTGCTAAAACAGCATTTACAGTATTTATTTGCTTTGTAAGTCTTGCAACTC

              4600         *        4620         *        4640         *        4660         *        4680
Guan  : TCTGGGTGTCGTCTTAAGTGTTCCGCACACCATAACGCTCTGTCCACGTATTCGCATCCTTTTCCCGATGAATAATATTTTTTAAAATCA : 4600
Zheng : TCTGGGTGTCGTCTTAAGTGTTCCGCACACCATAACGCTCTGTCCACGTATTCGCATCCTTTTCCCGATGAATAATATTTTTTAAAATCA : 4633
Liu   : TCTGGGTGTCGTCTTAAGTGTTCCGCACACCATGAGGCTCTGTCCACTTATTCGCATCCTTTTGCCGATGAATAATATTTTTTAAAATCA : 4610
        TCTGGGTGTCGTCTTAAGTGTTCCGCACACCATaAcGCTCTGTCCACgTATTCGCATCCTTTTcCCGATGAATAATATTTTTTAAAATCA

                 *        4700         *        4720         *        4740         *        4760         *
Guan  : AATTTAGTTGTCAAAATAAAAAAATTTAAATGAAAATGCAATTTAATAATTTGCATACGTACATGTATATTATTGCTAGTATTTAGTGCA : 4690
Zheng : AATTTAGTTGTCAAAATAAAAAAATTTAAATGAAAATGCAATTTAATAATTTGCATATGTACATGTATATTATTGCTAGTATTTAGTGCA : 4723
Liu   : AATCTAGTTGTCAAAATAAAAAAATTTAAATGAAAATGCAATTTAATAATTTGCATACGTACATGTATATTATTGCTAGTATTTAGTGCA : 4700
        AATtTAGTTGTCAAAATAAAAAAATTTAAATGAAAATGCAATTTAATAATTTGCATAcGTACATGTATATTATTGCTAGTATTTAGTGCA

              4780         *        4800         *        4820         *        4840         *        4860
Guan  : TATTATTGTCTATCTTTTCAACAACAATACAAATGAATAATTAATTACTGCTTTCTAACTGCTTGTTAAGTGAAAGGTTAACTCAATATT : 4780
Zheng : TATTATTGTCTATCTTTTCAACAACAATACAAATGAATAATTAATTACTGCTTTCTAACTGCTTGTTAAGTGAAAGGTTAACTCAATATT : 4813
Liu   : TATTATTGTCTATCTTTTCAACAACAATACAAATGAATAATTAATTACTGCTTTCTAACTGCTTGTTAAGTGAAAGGTTAACTCAATATT : 4790
        TATTATTGTCTATCTTTTCAACAACAATACAAATGAATAATTAATTACTGCTTTCTAACTGCTTGTTAAGTGAAAGGTTAACTCAATATT

                 *        4880         *        4900         *        4920         *        4940         *
Guan  : GGTAGAATTACGATCATCAACGGTATCGGGCTAAATTTTTTTTATTTTTTGTAATTATATTTATTATTTGAAATTATTTTTGTAATTAA : 4870
Zheng : GGTAGAATTACGATCATCAACGGTATCGGGCTAAATTTTTTTTATTTATTGTAATTATATTTATTATTTGAAATTATTTTTGTAATTAA : 4903
Liu   : GGTAGAATTACGATCATCAACGGTATCGGGCTAAATTTTTTTTATTTTTTGTAATTATATTTATTATTTGAAATTATTTTTGTAATTAA : 4880
        GGTAGAATTACGATCATCAACGGTATCGGGCTAAATTTTTTTTATTTtTTGTAATTATATTTATTATTTGAAATTATTTTTGTAATTAA
```

```
                4960         *       4980         *       5000         *       5020         *       5040
Guan  : AAATTTATTTGAGTAAATGGACTGCTTTTTTTATATTGGTAATATTCAATATTGCTATTCGAAATAATAAGTGTTTGACATCTTAAAGAT : 4960
Zheng : AAATTTATTTGAGTAAATGGACTGCTTTTTTTATATTGGTAATATTCAATATTGCTATTCGAAATAATAAGTGTTTGACATCTTAAAGAT : 4993
Liu   : AAATTTATTTGAGTAAATGGACTGCTTTTTTTATATTGGTAATATTCAATATTGCTATTCGAAATAATAAGTGTTTGACATCTTAAAGAT : 4970
        AAATTTATTTGAGTAAATGGACTGCTTTTTTTATATTGGTAATATTCAATATTGCTATTCGAAATAATAAGTGTTTGACATCTTAAAGAT

            *       5060         *       5080         *       5100         *       5120         *
Guan  : GAAAGGATTTTTGTTGGGTATTTTGTATAAAAAAAAGTGAATGAGTTATAGCTTTATCTGTATTTATTTTATTATTATATATCTATTAAAT : 5050
Zheng : GAAAGGATTTTTGTTGGGTATTTTGTATAAAAAAA-GTGAATGAGTTATAGCTTTATCTGTATTTATTTTATTATTATATATCTATTAAAT : 5082
Liu   : GAAAGGATTTTTGTTGGGTATTTTGTATAAAAAAAAGTGAATGAGTTATAGCTTTATCTGTATTTATTTTATTATTATATATCTATTAAAT : 5060
        GAAAGGATTTTTGTTGGGTATTTTGTATAAAAAAAaGTGAATGAGTTATAGCTTTATCTGTATTTATTTTATTATTATATATCTATTAAAT

                5140         *       5160         *       5180         *       5200         *       5220
Guan  : AAAATAAATCTCCCTCTTAATCTAAGTATTGTTTGTTATTCCCTTTAAAAGAAAAACAAGAAAA-TAATTTTTAAATGCTATTTACTCAT : 5139
Zheng : AAAATAAATCTCCCTCTTAATCTAAGTATTGTTTGTTATTCCCTTTAAAAGAAAAACAAGAAAA-TAATTTTTAAATGCTATTTACTCAT : 5171
Liu   : AAAATAAATCTCCCTCTTAATCTAAGTATTGTTTGTTATTCCCTTTAAAAGAAAAACAAGAAAAATAATTTTTAAATGCTATTTACTCAT : 5150
        AAAATAAATCTCCCTCTTAATCTAAGTATTGTTTGTTATTCCCTTTAAAAGAAAAACAAGAAAA TAATTTTTAAATGCTATTTACTCAT

            *       5240         *       5260         *       5280         *       5300         *
Guan  : TTTGCCAATATTATTATTAATATT------------------------------------------------------------------ : 5163
Zheng : TTTGCCAATATTATTATTAATATTGTAATAACCCGGGGTCCTCAAACTAATTATAGAAGGTCAGGTTTATTGAAAGTTGTCAGATGGCTT : 5261
Liu   : TTTGCCAATATTATTATTAATATT------------------------------------------------------------------ : 5174
        TTTGCCAATATTATTATTAATATT

                5320         *       5340         *       5360         *       5380         *       5400
Guan  : ------------------------------------------------------------------------------------------ : -
Zheng : GGAAGATGCAAAATGCTTAATTGTAATTATTAAGTCTTGAAATCTGCTGTTTCTATTACTCTATAACGATCTTGCCTTTCCGAGGTAAAT : 5351
Liu   : ------------------------------------------------------------------------------------------ : -

            *       5420         *       5440         *       5460         *       5480         *
Guan  : ------------------------------------------------------------------------------------------ : -
Zheng : CCATTTGTCAAGGTACATAACCAAAGAGTCTGAATAGCACTTTCAATTTTTCAAAATTCAAATTTATTCTCAAATTATATTGCCACTAAA : 5441
Liu   : ------------------------------------------------------------------------------------------ : -

                5500         *       5520         *       5540         *       5560         *       5580
Guan  : ----------------------------------------------------------------------------ATGATATCATTATC : 5177
Zheng : TTTAATTAATTATTAAATTAATTAATCGGCAATATAATTTTAAATATTATTCAATTTCTAGGGATCCAACAATATTATGATATCATTATC : 5531
Liu   : ----------------------------------------------------------------------------ATGATATCATTATC : 5188
                                                                                    ATGATATCATTATC

            *       5600         *       5620         *       5640         *       5660         *
Guan  : GTCTGGCTACAATCATATTAAATTTAAAATTTTAAATGTATTTTGAATAAGGGATTT--TTTTTAAAGACAATCGGTTTCTGGGATTAAT : 5265
Zheng : GTCTGGCTACAATCATATTAAATTTAAAATTTTAAATATATTTTGAACAAGGGATTTGTTTTTTAAAGACAATTGGTTTCTTGGATTAAT : 5621
Liu   : GTCTGGCTACAATCATATTAAATTTAAAATTTTAAATGTATTTTGAATAAGGGATTT--TTTTTAAAGACAATCGGTTTCTGGGATTAAT : 5276
        GTCTGGCTACAATCATATTAAATTTAAAATTTTAAATgTATTTTGAAtAAGGGATTT  TTTTTAAAGACAATcGGTTTCTgGGATTAAT

                5680         *       5700         *       5720         *       5740         *       5760
Guan  : ACATTTAAGTCGTCAGAAAGAAGAATGAGTTTTGCTAGATATTAAATATTTATCAA---------TTATAAAATAAATGCGGATAGTTAAA : 5346
Zheng : ACATTTAAGTCGTCAGAAAGAAGAATGAGTTTTGCTAGATATTAGTATTTATCAAAATCGAGAATTATGAAATAAATGCGGATAATCGGA : 5711
Liu   : ACATTTAAGTCGTCAGAAAGAAGAATGAGTTTTGCTAGATATTAAATATTTATCAA---------TTATAAAATAAATGCGGATAGTTAAA : 5357
        ACATTTAAGTCGTCAGAAAGAAGAATGAGTTTTGCTAGATATTAaTATTTATCAA         TTATaAAATAAATGCGGATAgTtaaA

            *       5780         *       5800         *       5820         *       5840         *
Guan  : ATAGAATTTAAGGTGTTT-TTTGAGTAGAAATTTTATATTTATTCATTATCAATTTAAATTTATTGATAGAAA--TTAATAAACATGCAA : 5433
Zheng : ATAGAATTTAAGGTGTTTGTTTAATTAGAAATTTTATAGTTATCCATTATCAATTTAAAGTTATTGATAGATAACTTAATATATATGGGG : 5801
Liu   : ATAGAATTTAAGGTGTTT-TTTGAGTAGAAATTTTATATTTATTCATTATCAATTTAAATTTATTGATAGAAA--TTAATAAACATGCAA : 5444
        ATAGAATTTAAGGTGTTT TTTgAgTAGAAATTTTATAtTTATtCATTATCAATTTAAAtTTATTGATAGAaA  TTAATAaAcATGcaa

                5860         *       5880         *       5900         *       5920         *       5940
Guan  : AACAGCTGTTAATATGGATCTGTTAATAATTGATTTTTTTTTGTCTTTGGCTAGAAAAGATATCAAATCGCATGTGAGTCCGTGCTTTTT : 5523
Zheng : AACAGTTGTTAATATGGATTTGTTAATAATTGATTTTTTTTTGTCTTTGGCCAGAAAAGATATCAAATTGTATGTGAGTCCGTGCTTTTT : 5891
Liu   : AACAGCTGTTAATATGGATCTGTTAATAATTGATTTTTTTTTGTCTTTGGCTAGACAAGATATCAAATCGCATGTGAGTCCGTGCTTTTT : 5534
        AACAGcTGTTAATATGGATcTGTTAATAATTGATTTTTTTTTGTCTTTGGCtAGAaAAGATATCAAATcGcATGTGAGTCCGTGCTTTTT

            *       5960         *       5980         *       6000         *       6020         *
Guan  : GAAACAAATTCACACGGAATACTTTTAAAATAACGATTATTTTCTATTGAATGTTTGTATTGAAATATATTAAGTTCATTATTTAGGGAG : 5613
Zheng : GAAACAAATTCACACGGAATACTTTTAAAATAACGATTATTTTCTATTGATTGTTTGTATT---------TAAGTTCATTATTTAGGGAG : 5972
Liu   : GAAACAAATTCACACGGAATACTTTTAAAATAACGATTATTTTCTATTGATTGTTTGTATTGAAATATATTAAGTTCATTATTTAGGGAG : 5624
        GAAACAAATTCACACGGAATACTTTTAAAATAACGATTATTTTCTATTGAtTGTTTGTATTgaaatatatTAAGTTCATTATTTAGGGAG

                6040         *       6060         *       6080         *       6100         *       6120
Guan  : TCTTTAATTTATTCAAGTGTCTTGTCCAAACAACGAACAAGAATTGCTTCTCTATTTAAGTGCATAACTTCCTGCTGTACACCGAACTAG : 5703
Zheng : TCTTTAATTTATTCAAGTGTCTTGTCCAAACAACGAACAAGAATTGCTTCTCTATTTAAGTGCATAACTTCCTGCTGTACACCGAACTAG : 6062
Liu   : TCTTTAATTTATTCAAGTGTCTTGTCCAAACAACGAACAAGAATTGCTTCTCTATTTAAGTGCATAACTTCCTGCTGTACACCGAACTAG : 5714
        TCTTTAATTTATTCAAGTGTCTTGTCCAAACAACGAACAAGAATTGCTTCTCTATTTAAGTGCATAACTTCCTGCTGTACACCGAACTAG

            *       6140         *       6160         *       6180         *       6200         *
Guan  : AATCAACTGAATTCATCATGTTAGTTCTGATAATTTGTCTTCTACTGTCCTTTTCATTGGAAATCGATGCAAATAGTTCAGATAGTGAGT : 5793
Zheng : AATCAACTGAATTCATCATGTTAGTTTTGATAATTTGTCTTCTACTGTCCTTTTCTTTGGTCATCGATGCAAATAGTTCAAATAGTGAGT : 6152
Liu   : AATCAACTGAATTCATCATGTTAGTTCTGATAATTTGTCTTCTACTGTCCTTTTCTTTGGTCATCGATGCAAATAGTTCAGATAGTGAGT : 5804
        AATCAACTGAATTCATCATGTTAGTTcTGATAATTTGTCTTCTACTGTCCTTTTCtTTGGtcATCGATGCAAATAGTTCAgATAGTGAGT
```

```
                6220         *        6240         *        6260         *        6280         *        6300
Guan  : TTACACGATTCATTTGAGAAGATATATCAAAACATATGTATTATTTTTTCGATTGATATTTTAAAAGATTTT----TTTA--CCTAAATT : 5877
Zheng : TTATACGATTAATTTGAGAATATATATCAAAACATATGTATTATTTTTTATATTACTTTTTATTTTAATTTTAAACTGTAATTTTTAATT : 6242
Liu   : TTACACGATTCATTTGAGAAGATATATCAAAACATATGTATTATTTTTTCGATTGATATTTTAAAAGATTTT----TTTA--CCTAAATT : 5888
        TTAcACGATTcATTTGAGAAgATATATCAAAACATATGTATTATTTTTTcgATTgaTaTTTtaaaagATTTT    TtTA  ccTaAATT

                    *        6320         *        6340         *        6360         *        6380         *
Guan  : CTTGTAAATAAAGATTAAAAATAAATAGTTATATTACAAATAAAATGCTATCGATATATTCGTTATTGTTATATTATTAATCTGTAGTAA : 5967
Zheng : CTTGTAAATAAAGATTAAAAATAAATAGTTATATTACAAATAAAATGCTATCGATATATTCGTTATTGTTATATTATTAATCTGTAGTAA : 6332
Liu   : CTTGTAAATAAAGATTAAAAATAAATAGTTATATTACAAATAAAATGCTATCGATATATTCGTTATTGTTATATTATTAATCTGTAGTAA : 5978
        CTTGTAAATAAAGATTAAAAATAAATAGTTATATTACAAATAAAATGCTATCGATATATTCGTTATTGTTATATTATTAATCTGTAGTAA

                6400         *        6420         *        6440         *        6460         *        6480
Guan  : CATTAATATTCAATTCAGCACCAAAGGATTGCCACTCAGTGGCGAAAGACAAATTTGGATTACCAACCGCTCCTTGTATTTGTGCTTGGG : 6057
Zheng : CATTAATATTCAATTCAGCACCGAAGGATTGCCAATTAGTGGCGAAAGACAAATTTGGATTACCAACCGCTCCTTGTATTTGTGCTTGGG : 6422
Liu   : CATTAATATTCAATTCAGCACCAAAGGATTGCCACTCAGTGGCGAAAGACAAATTTGGATTACCAACCGCTCCTTGTATTTGTGCTTGGG : 6068
        CATTAATATTCAATTCAGCACCaAAGGATTGCCAcTcAGTGGCGAAAGACAAATTTGGATTACCAACCGCTCCTTGTATTTGTGCTTGGG

                    *        6500         *        6520         *        6540         *        6560         *
Guan  : TTAGTCACTGTTTTGATCATTTTTGATAATATGTCTGTTCATTAAAAACATGCTTATATGCAATTTCTCAATTTGGTATCACTCACGACA : 6147
Zheng : TTAGTCACTG-----ATCAGTTTTGATAATA--TCTACTTATTAAAGATATGCTTATATGCATTTTCTCAATTTGGTATCACTCACGACA : 6505
Liu   : TTAGTCACTGTTTTGATCATTTTTGATAATATGTCTGTTCATTAAAAACATGCTTATATGCAATTTCTCAATTTGGTATCACTCACGACA : 6158
        TTAGTCACTGttttgATCAtTTTTGATAATAtgTCTgtTcATTAAAAaAcATGCTTATATGCAaTTTCTCAATTTGGTATCACTCACGACA

                6580         *        6600         *        6620         *        6640         *        6660
Guan  : TTTATATAAATTGTGAAGCTGATAAATAGTTTAGAAAATACTATGAGGCCATGCCATTATTTATCAAGGATAAGGAGTGTGCAGAACACG : 6237
Zheng : TTTATATAAATTCTGAAGCTGATAAATAGTTTAGAAAATACTATGAAGCCATGCCATTATTTATCAAGGATAAGGAGTGTGCAGAACACG : 6595
Liu   : TTTATATAAATTGTGAAGCTGATAAATAGTTTAGAAAATACTATGAGGCCATGCCATTATTTATCAAGGATAAGGAGTGTGCAGAACACG : 6248
        TTTATATAAATTgTGAAGCTGATAAATAGTTTAGAAAATACTATGAgGCCATGCCATTATTTATCAAGGATAAGGAGTGTGCAGAACACG

                    *        6680         *        6700         *        6720         *        6740         *
Guan  : AATATTGTCAACTTGCTAGAGGAGACAATATCAACCAATGCTTAGGAAGTAAGTTTTGAGTAAATTGCTGAATGATAATGAAGAATAAAG : 6327
Zheng : AATATTGTCAACTTGCTAGAGGAGACAATATCAACCAATGCTTAGGAAGTAAGTTTTGAGTAAATTGC-------------GAAATGAAG : 6672
Liu   : AATATTGTCAACTTGCTAGAGGAGACAATATCAACCAATGCTTAGGAAGTAAGTTTTGAGTAAATTGCTGAATGATAATGAAGAATAAAG : 6338
        AATATTGTCAACTTGCTAGAGGAGACAATATCAACCAATGCTTAGGAAGTAAGTTTTGAGTAAATTGCtgaatgataatgaagAATaAAG

                6760         *        6780         *        6800         *        6820         *        6840
Guan  : AATAATGTCATAATTTAATGTTGCCCTTTTAAAATCAGAATATTGCACTGGTGACAAGACTGATGCTCCAATGTGCATTTGTGGAGTAAG : 6417
Zheng : AATA--GTCATCATTTAATGTTGCCCTTTTAAAATCAGAATATTGCACTGGTGAAAAGACTGATGCTCCAATGTGCGTTTGTGGAGTAAG : 6760
Liu   : AATAATGTCATAATTTAATGTTGCCCTTTTAAAATCAGAATATTGCACTGGTGAAAAGACTGATGCTCCAATGTGCGTTTGTGGAGTAAG : 6428
        AATAatGTCATaATTTAATGTTGCCCTTTTAAAATCAGAATATTGCACTGGTGAaAAGACTGATGCTCCAATGTGCgTTTGTGGAGTAAG

                    *        6860         *        6880         *        6900         *        6920         *
Guan  : TGAAATTTTATAAAATATCTATTATTATTATTAATGTTATTGTTATTATTATGAGAAAATAATAACATATCTTTTGTGTTTCAGGATAAG : 6507
Zheng : TGAAATTTTATAAAATATCTATTATTAT---TAATGTTATTATTATTATTATGAGAAAATAATGACATATCTTTT-TGTTTCAGGATAAG : 6846
Liu   : TGAAATTTTATAAAATATCTATTATTATTATTAATGTTATTATTATTATTATGAGAAAATAATAACATATCTTTTGTGTTTCAGGATAAG : 6518
        TGAAATTTTATAAAATATCTATTATTATtatTAATGTTATTaTTATTATTATGAGAAAATAATaACATATCTTTTgTGTTTCAGGATAAG

                6940         *        6960         *        6980         *        7000         *        7020
Guan  : CGATGCGGGTATAACGAGGACTGTAAGAAAGTTGAGGATAAATTACAATGTGTCGAAAGTGAGTTTCTCTTAGCTGCTTTTGTTGTGCTT : 6597
Zheng : CGATGCGGGTATAACGAGGACTGCAAGAAAGTTG-GGATAAATTACAATGTGTCGAAAGTGAGTATCTCTTAGCTGTTTTT-TTGTGCTT : 6934
Liu   : CGATGCGGGTATAACGAGGACTGTAAGAAAGTTGAGGATAAATTACAATGTGTCGAAAGTGAGTTTCTCTTAGCTGCTTTTGTTGTGCTT : 6608
        CGATGCGGGTATAACGAGGACTGtAAGAAAGTTGaGGATAAATTACAATGTGTCGAAAGTGAGTtTCTCTTAGCTGcTTTTgTTGTGCTT

                    *        7040         *        7060         *        7080         *        7100         *
Guan  : TGAAATTGCTGCAACCACGATTTTAAAACAAAATATGATTGTATACAAGGATCATATTTCATAAACGAGTCCATCTCTTCTTTAAATCTT : 6687
Zheng : TGAAATTGCTGCAACCACGATTTTAAAACAAAATATGATTGTACAGAAGGATCATATTTCATACACGAGTCCATGCCTTTTTAAAATCTT : 7024
Liu   : TGAAATTGCTGCAACCACGATTTTAAAACAAAATATGATTGTATACAAGGATCATATTTCATACACGAGTCCATGCCTTTTTAAAATCTT : 6698
        TGAAATTGCTGCAACCACGATTTTAAAACAAAATATGATTGTAtAcAAGGATCATATTTCATAcACGAGTCCATgcCTTtTTaAAATCTT

                7120         *        7140         *        7160         *        7180         *        7200
Guan  : GTTCGTACAGGATAATAGCGACTCGTAATTTTTTCTGTTCTTTTTCGACTTTGGGAAAATTAATTTAGAATTAATATTAATTCAT-CGAT : 6776
Zheng : GTTCGTACGTGTTAATAGCGACTCGTGATTTTTTCTGTT-------GATTTTGTGAAAATTAATTTAGAATTAATGTTAATTCACTCGAT : 7107
Liu   : GGTCGTACAGGATAATAGCGACTCGTGATTTTTTCTGTT-------GACTTTGTGAAAATTAATATAGACTGAAAATTAATTCATTCGAT : 6781
        GtTCGTACagGaTAATAGCGACTCGTgATTTTTTCTGTT       GAcTTTGtGAAAATTAATtTAGAaTtAAtaTTAATTCAttCGAT

                    *        7220         *        7240         *        7260         *        7280         *
Guan  : AATTGCTTTGAGTTCTGCATTTACTCATGTACCTTGTTCAATCATCGGACGTGAGTGATCTCAACTCAAGGACTTTATGCAAAGTTTAGA : 6866
Zheng : AATTACATTGAGTTTTGTATTTACTCATGTACGT-GATCAATCATCGGACGTGAGTGATCTCAACTCAAAAG-TTTATACAAAGTTTAGA : 7195
Liu   : AATTGCTTTGAGTTCTGCATTTACTCATGTACCTTGTTCAATCATCGGACGTGAGTGATCTCAACTCAAGGACTTTATGCAAAGTTTAGA : 6871
        AATTgCtTTGAGTTcTGcATTTACTCATGTACcTtGtTCAATCATCGGACGTGAGTGATCTCAACTCAAggacTTTATgCAAAGTTTAGA

                7300         *        7320         *        7340         *        7360         *        7380
Guan  : TATGTCCCTTCACAATGATAGGCTTGACAATAATTTGGCACAGGATAACATTTTTTTTAAATGAGTGATTTGAAGATATCCTCAAAGTTG : 6956
Zheng : TATATCTCTTCACAATGATAGGCTTTACAATAATTTGCCACAGGATAACATTTTTTTTAAATGAGTGATTTGAATATATCCTCATAGTTG : 7285
Liu   : TATGTCCCTTCACAATGATAGGCTTGACAATAATTTGGCACAGGATAACATTTTTTTTAAATGAGTGATTTGAAGATATCCTCAAAGTTG : 6961
        TATgTCcCTTCACAATGATAGGCTTgACAATAATTTGgCACAGGATAACATTTTTTTTAAATGAGTGATTTGAAgATATCCTCAaAGTTG

                    *        7400         *        7420         *        7440         *        7460         *
Guan  : AGTTGAGATGCTTTAGGAGCATGTCTTTCAGAAAGGGTCTAAACACTTCACAATGACTGT-TTTTTTTTTATTATTTTTTTAAATTTAAA : 7045
Zheng : AATTAAGATGCGTTAGGAGCATGTCTTTCAGAAACGTTCTAAACACTTCACAATGACTGTGTTTTTTTTTATTATTTTTTTAAATTTAAA : 7375
Liu   : AGTTGAGATGCTTTAGGAGCATGTCTTTCAGAAAGGGTCTAAACACTTCACAATGACTGT-TTTTTTTTTATTATTTTTTTAAATTTAAA : 7050
        AgTTgAGATGCtTTAGGAGCATGTCTTTCAGAAAgGgTCTAAACACTTCACAATGACTGT TTTTTTTTTATTATTTTTTTAAATTTAAA
```

```
                7480          *       7500          *       7520          *       7540          *       7560
Guan  : AAATGATTTTTTAATAAATTCTTCACGAACTGGAGTACGAATTCTTTGGATATCGCAGGAAGACAGCATTGATTTATTTCAGAAGTAACA : 7135
Zheng : AAATGATTTTTTAATAAATTTTTCACGAACTGGAGTACGAATTCTTTGGATATCGCAGGAAGACAGCATTGATTTATTTCAGAAGTAACA : 7465
Liu   : AAATGATTTTTTAATAAATTCTTCACGAACTGGAGTACGAATTCTTTGGATATCGCAGGAAGACAGCATTGATTTATTTCAGAAGTAACA : 7140
        AAATGATTTTTTAATAAATTcTTCACGAACTGGAGTACGAATTCTTTGGATATCGCAGGAAGACAGCATTGATTTATTTCAGAAGTAACA

                 *       7580          *       7600          *       7620          *       7640          *
Guan  : TGAGGAAGTTGACTAAAGATTTAGTAGCATAGTAGTTTCTTTATCCTGAATGCTGAAAGATATATATCCGG----ATATAA-GATAAGCT : 7220
Zheng : TGAGGAAGTTGACTAAAGATTTAGTAGCATAGTAGTTTCTTTATCCTAAATGCTGAAAGATATATATCCGG----ATATAAAGATAAGCT : 7551
Liu   : TGAGGAAGTTGACTAAAGATTTAGTAGCATAGTAGTTTCTTTATCCTGAATGCTGAAAGATATATATCCGGTGATATATAA-GATAAGCT : 7229
        TGAGGAAGTTGACTAAAGATTTAGTAGCATAGTAGTTTCTTTATCCTgAATGCTGAAAGATATATATCCGG    ATATAA GATAAGCT

                7660          *       7680          *       7700          *       7720          *       7740
Guan  : AGTTCGTTATAATCGAAAAGATGAAA----------ATATATACATACATTTGGTAAAGGTGAGGTCACTTAACATTAATATTAATTGCA : 7300
Zheng : AGTTCGTTATAATCGAAAAGATGAAATTTCAACGAAATATATACATACATTTGGTAAAGGTGAGGTCACTTAACATTAATATTAATTGCA : 7641
Liu   : AGTTCGTTATAATCGAAAAGATGAAATTTCAACGAAATATATACATACATTTGGTAAAGGTGAGGTCACTTAACATTAATATTAATTGCA : 7319
        AGTTCGTTATAATCGAAAAGATGAAAtttcaacgaaATATATACATACATTTGGTAAAGGTGAGGTCACTTAACATTAATATTAATTGCA

                 *       7760          *       7780          *       7800          *       7820          *
Guan  : GAGCTGAAACAATGTTCAAGTGTGGAAAAAGATGAATATGGAATTCCAATTGGTCCTTGTAGTTGTGGCTGGGTTAGTCTTGATCAATTA : 7390
Zheng : GAGCTGAAGCAATGTTCAAGTGTGGAAAAAGATGAATATGGAATTCCAATTGGTCCTTGTAGTTGTGGTTGGGTTAGTCTTGATCAATTA : 7731
Liu   : GAGCTGAAACAATGTTCAAGTGTGGAAAAAGATGAATATGGAATTCCAATTGGTCCTTGTAGTTGTGGCTGGGTTAGTCTTGATCAATTA : 7409
        GAGCTGAAaCAATGTTCAAGTGTGGAAAAAGATGAATATGGAATTCCAATTGGTCCTTGTAGTTGTGGcTGGGTTAGTCTTGATCAATTA

                7840          *       7860          *       7880          *       7900          *       7920
Guan  : TTGATAATAACACATCCACCTACACACTCAACCAATTGACACTATGGTTTAGAAAACTTTATGCTGAAATCGTATCATTTTTTTACCAAG : 7480
Zheng : TTGATAATAACACATCCACCTACACACTCAACCAATTGATACTATGGTTTAGAAAACTTTGTGCTGAAATAGTACCATTTTTA-ACCAAG : 7820
Liu   : TTGATAATAACACATCCACCTACACACTCAACCAATTGACACTATGGTTTAGAAAACTTTATGCTGAAATCGTATCATTTTTTTACCAAG : 7499
        TTGATAATAACACATCCACCTACACACTCAACCAATTGAcACTATGGTTTAGAAAACTTTaTGCTGAAATcGTAtCATTTTTttACCAAG

                 *       7940          *       7960          *       7980          *       8000          *
Guan  : GATTTTATGGAGTGTGAAAAGAACGAATATTGTGATATTAGTGATGGATTCTTAGGCTTTTGTGAGCGATGTAAGTTCCGAGTATATTGC : 7570
Zheng : GATTTTCTGGAGTGTGAAAAGAACGAATATTGTGATATTAGTGATGGATTCCTAGGCTTTTGTGAGCGATGTAAGTTCCGAGTATATTGC : 7910
Liu   : GATTTTCTGGAGTGTGAAAAGAACGAATATTGTGATATTAGTGATGGATTCTTAGGCTTTTGTGAGCGATGTAAGTTCCGAGTATATTGC : 7589
        GATTTTcTGGAGTGTGAAAAGAACGAATATTGTGATATTAGTGATGGATTCtTAGGCTTTTGTGAGCGATGTAAGTTCCGAGTATATTGC

                8020          *       8040          *       8060          *       8080          *       8100
Guan  : GAAATAATAAT---GTTATCAATTGGTGATGAAGTTTGACATTTTTTTCTCACATAAACGTTTAAAAATCAGTGTATTGTTCCGGTGACC : 7657
Zheng : GAAATAATAATAATGTTATCAATTGGTGATGAAGTTTGACATTTTTTTCTCACATAAACGTTTAAAAATCAGTGTATTGTTCCGGTGACC : 8000
Liu   : GAAATAATAATAATGTTATCAATTGGTGATGAAGTTTGACATTTTTTTCTCACATAAACGTTTAAAAATCAGTGTATTGTTCCGGTGACC : 7679
        GAAATAATAATaatGTTATCAATTGGTGATGAAGTTTGACATTTTTTTCTCACATAAACGTTTAAAAATCAGTGTATTGTTCCGGTGACC

                 *       8120          *       8140          *       8160          *       8180          *
Guan  : CGGATAAAGATTCTCCAGTGTGCATATGTGAAGTCAGTAACATCTTATCTAATGCTAATAATTATTATTATAAATTAATAATAATAATAA : 7747
Zheng : CGGATAAAGATTCTCCAGTGTGCATATGTGAAGTCAGTAACATCTTATCTAATGCTAATAATTATTATTATAAATTAATAATAATAATAA : 8090
Liu   : CGGATAAAGATTCTCCAGTGTGCATATGTGAAGTCAGTAACATCTTATCTAATGCTAATAATTATTATTATAAATTAATAATAATAATAA : 7769
        CGGATAAAGATTCTCCAGTGTGCATATGTGAAGTCAGTAACATCTTATCTAATGCTAATAATTATTATTATAAATTAATAATAATAATAA

                8200          *       8220          *       8240          *       8260          *       8280
Guan  : TAAAGATAATTATGACGAGAAAATGAAGAAATGTCTTTTGTCTTTCAGGATAAGCGATGCAACCATGATCAATACTGTAGGAACGTTGAT : 7837
Zheng : TAAAGATAATTATGACGAGAAAATGAAGAAATGTCTTTTGTCTTTCAGGATAAGCGATGCAACCATGATCAATACTGTGCGAACGTTGAT : 8180
Liu   : TAAAGATAATTATGACGAGAAAATGAAGAAATGTCTTTTGTCTTTCAGGATAAGCGATGCAACCATGATCAATACTGTGCGAACGTTGAT : 7859
        TAAAGATAATTATGACGAGAAAATGAAGAAATGTCTTTTGTCTTTCAGGATAAGCGATGCAACCATGATCAATACTGTgcGAACGTTGAT

                 *       8300          *       8320          *       8340          *       8360          *
Guan  : GGCAAGTTGGTATGTCATGAATGTGAGTTTGTCTTAGCTGTTTT----TTTTATTGTGAGACACGTTATTTTAAAGTTGTTAATATCACG : 7923
Zheng : GGCAAGTTGGTATGTCATGAATGTGAGTTTGTCTTAATTGTTTTGTTTTTTTATTGTGAGACACGTTATTTTAAAGTTGTTAATATCACG : 8270
Liu   : GGCAAGTTGGTATGTCATGAATGTGAGTTTGTCTTAGTTGTTTT----TTTTATTGTGAGACACGTTATTTTAAAGTTGTTAATATCACG : 7945
        GGCAAGTTGGTATGTCATGAATGTGAGTTTGTCTTAgtTGTTTT    TTTTATTGTGAGACACGTTATTTTAAAGTTGTTAATATCACG

                8380          *       8400          *       8420          *       8440          *       8460
Guan  : ATTT-AAAAAAATATTATTATATAGTATTACATATAATAATTTATTTATATAATATTATTATTAACAACGTTATTATTATTAATATTAGAGA : 8012
Zheng : ATTTTAAAAAAATATTATTATATAGTATTACATATAATAATTTATTTATATAATATTATTATTAACAACGTTATTATTATTAATATTAGAGA : 8360
Liu   : ATTT-AAAAAAATATTATTATATAGTATTACATATAATAATTTATTTATATAATATTATTATTAACAACGTTATTATTATTAATATTAGAGA : 8034
        ATTT AAAAAAATATTATTATATAGTATTACATATAATAATTTATTTATATAATATTATTATTAACAACGTTATTATTATTAATATTAGAGA

                 *       8480          *       8500          *       8520          *       8540          *
Guan  : ATTTCTTGATATTAATATTGATATTATTTTATTATTAATATAAATATTATTTTTAATAAGAAGACGATATAAGCATATAATTGGAATATC : 8102
Zheng : ATTTCTTGATATTAATATTGATATTATTTTATTATTAATATAAATATTATTTTTAATAAGAAGACGATA-AAGCATATAATAG-AATATC : 8448
Liu   : ATTTCTTGATATTAATATTGATATTATTTTATTATTAATATAAATATTATTTTTAATAAGAAGACGATATAAGCATATAATAGGAATATC : 8124
        ATTTCTTGATATTAATATTGATATTATTTTATTATTAATATAAATATTATTTTTAATAAGAAGACGATAtAAGCATATAATaGgAATATC

                8560          *       8580          *       8600          *       8620          *       8640
Guan  : ATTTTTATTAATAAAATACTATAAGAATTCCTAAATCACAATATATTAGCAATGTTAATATTTTCTTTTAGCTTTTTTGTTTTTATCTCC : 8192
Zheng : ATTTTTATTATTAAA-TACTATAAGAATTCCTAAATCACAATATATTAGCAATGTTAATATTTTCTTTTAGCTTTTTTGTTTTTATCTCC : 8537
Liu   : ATTTTTATTAATAAAATACTATAAGAATTCCTAAATCACAATATATTAGCAATGTTAATATTTTCTTTTAGCTTTTTTGTTTTTATCTCC : 8214
        ATTTTTATTAaTAAAaTACTATAAGAATTCCTAAATCACAATATATTAGCAATGTTAATATTTTCTTTTAGCTTTTTTGTTTTTATCTCC

                 *       8660          *       8680          *       8700          *       8720          *
Guan  : AGGACTCTGAATGTAGTACCAAAGACAGATGCATTGTCACAAAAGGAGATTATAGTAACTACTGCGATTGACAATAAACTCCTTACTTTG : 8282
Zheng : AGAACTCTGAATGTAGTACCAAAGACAGATGCATTGTCACAAAAGGAGATTATAGTAACTACTGCGATTGACAATAAACTCCTTACTTTG : 8627
Liu   : AGGACTCTGAATGTAGTACCAAAGACAGATGCATTGTCACAAAAGGAGATTATAGTAACTACTGCGATTGACAATAAACTCCTTACTTTG : 8304
        AGgACTCTGAATGTAGTACCAAAGACAGATGCATTGTCACAAAAGGAGATTATAGTAACTACTGCGATTGACAATAAACTCCTTACTTTG
```

```
                 8740         *        8760         *        8780         *        8800         *        8820
Guan  : TTTGCTCATCTGTTATTAACCTGAATTATGGCAATAAAATCCGAAATTTAAAGGCAAATTATTTGTGATTTAATATAATTACATAAATTT : 8372
Zheng : TTTGCTCATCTGTTATTAACCTGAATTATGGCAATAAAATCCGAAATTTAAAGGCAAATTATTTGTGATTTAATATAATTAAATAAATTT : 8717
Liu   : TTTGCTCATCTGTTATTAACCTGAATTATGGCAATAAAATCCGAAATTTAAAGGCAAATTATTTGTGATTTAATATAATTACATAAATTT : 8394
        TTTGCTCATCTGTTATTAACCTGAATTATGGCAATAAAATCCGAAATTTAAAGGCAAATTATTTGTGATTTAATATAATTAcATAAATTT

                   *        8840         *        8860         *        8880         *        8900         *
Guan  : AATGCAATAAAAAACAATTTATTTGTGTATCGCTGTCTTTAGGTACCCTAACGAACTAATCGCTGCTAATAGGTATGCAAACCCTTGAGA : 8462
Zheng : AATGCAATAAAAAA--ATTTATTTGTGTATCGCTGTCTTTAGGTACCCTAACGAACTAATCGCTGCTAATAGATATGCAAACCCTTGAGA : 8805
Liu   : AATGCAATAAAAAACAATTTATTTGTGTATCGCTGTCTTTAGGTACCCTAACGAACTAATCGCTGCTAATAGGTATGCAAACCCTTGAGA : 8484
        AATGCAATAAAAAAcaATTTATTTGTGTATCGCTGTCTTTAGGTACCCTAACGAACTAATCGCTGCTAATAGgTATGCAAACCCTTGAGA

                 8920         *        8940         *        8960         *        8980         *        9000
Guan  : ATGCATAATTCTTACATTTTAATTCATACGATAATA-TTTTGTTTTGCATATCTTTGAAACTGTAGATTTATATTTACATTTTTAATATT : 8551
Zheng : TTGCATAATTCTTACATTTTAATTCATACGATAATAATTTTGTTTTGCATATCTTTGAAACTGTAGATTTATATTTGCATTTTAAATATT : 8895
Liu   : TTGCATAATTCTTACATTTTAATTCATACGATAATA-TTTTGTTTTGCATATCTTTGAAACTGTAGATTTATATTTACATTTTTAATATT : 8573
        tTGCATAATTCTTACATTTTAATTCATACGATAATA TTTTGTTTTGCATATCTTTGAAACTGTAGATTTATATTTaCATTTTtAATATT

                   *        9020         *        9040         *        9060         *        9080         *
Guan  : AAATACGCTAGATAAGGCAGATTTCGCAATGTTATAGTATAGGGATCTTATTTAATTTAACACCTTATTTTGTATTTTGAAATCTAATAC : 8641
Zheng : AAATACGCTAGATAAGGCAGATTTCGCAATGTTATAGTATAGGGATCTTATTTAATTTAACACCTTATTTTGTATTTTGAAATCTAATAC : 8985
Liu   : AAATACGCTAGATAAGGCAGATTTCGCAATGTTATAGTATAGGGATCTTATTTAATTTAACACCTTATTTTGTATTTTGAAATCTAATAC : 8663
        AAATACGCTAGATAAGGCAGATTTCGCAATGTTATAGTATAGGGATCTTATTTAATTTAACACCTTATTTTGTATTTTGAAATCTAATAC

                 9100         *        9120         *        9140         *        9160         *        9180
Guan  : GATCTGTCCTCATGGCACTCCCTAGTTTGTCTTCAGCACATATTTATAAATAAGGAGGAGGAAATTGAAATAACATCCGCATTTCTAATA : 8731
Zheng : GATCTGTCCTCATGGCACTCCCTAGTTTGTCTTCAGCACATATTTATAAATAAGGAAGAGGAAATTGAAATAACATCCGCATTTCTAATA : 9075
Liu   : GATCTGTCCTCATGGCACTCCCTAGTTTGTCTTCAGCACATATTTATAAATAAGGAAGAGGAAATTGAAATAACATCCGCATTTCTAATA : 8753
        GATCTGTCCTCATGGCACTCCCTAGTTTGTCTTCAGCACATATTTATAAATAAGGAaGAGGAAATTGAAATAACATCCGCATTTCTAATA

                   *        9200         *        9220         *        9240         *        9260         *
Guan  : ATTTTATATATTTAATTCTTTTAACAATTCAATATAATATATTTTCAGATTTTATTGATTTCCAATTTTTTTTT-GCTAATCTCAACAT : 8820
Zheng : ATTTTATATATTTAATTCTTTTAACAATTCAATATAATATATTTTCAGATTTTATTGATTTCCAATTTTTTTTT-GCTAATCTCAACAT : 9164
Liu   : ATTTTATATATTTAATTCTTTTAACAATTCAATATAATATATTTTCAGATTTTATTGATTTCCAATTTTTTTTTTGCTAATCTCAACAT : 8843
        ATTTTATATATTTAATTCTTTTAACAATTCAATATAATATATTTTCAGATTTTATTGATTTCCAATTTTTTTTT GCTAATCTCAACAT

                 9280         *        9300         *        9320         *        9340         *        9360
Guan  : GTCGAAACATCGTGTTTAAAACCATAGCGACCAAATGATTGAAGTTTAAAAGTCAATGCTTTAGCACAGATGAATTTTCAGAAGGTCGCT : 8910
Zheng : GTCGAAACATCGTGTTTAAAACCATAGCGACCAAATGATTAAAGTTTAAAAGTCAATGCTTTAGCACAGATGAATTTTCAGAAGGTCGCT : 9254
Liu   : GTCGAAACATCGTGTTTAAAACCATAGCGACCAAATGATTGAAGTTTAAAAGTCAATGCTTTAGCACAGATGAATTTTCAGAAGGTCGCT : 8933
        GTCGAAACATCGTGTTTAAAACCATAGCGACCAAATGATTgAAGTTTAAAAGTCAATGCTTTAGCACAGATGAATTTTCAGAAGGTCGCT

                   *        9380         *        9400         *        9420         *        9440         *
Guan  : AGACTAAACTAGAAAAACTAAGAGACAGGGTCGGCCTGATGACGAATTTATTATCACAAGATTAAACTAGATTAAACTTTATACATAATA : 9000
Zheng : AGACCAAACTAGAAAAACTAAGAGACAGGGTCGGCCTGATGACGAATTTATTATCACAAGATTAAACTAGATTAAACTTTATACATAATA : 9344
Liu   : AGACTAAACTAGAAAAACTAAGAGACAGGGTCGGCCTGATGACGAATTTATTATCACAAGATTAAACTAGATTAATCTTTATACATTATA : 9023
        AGACtAAACTAGAAAAACTAAGAGACAGGGTCGGCCTGATGACGAATTTATTATCACAAGATTAAACTAGATTAAaCTTTATACATaATA

                 9460         *        9480         *        9500         *        9520         *        9540
Guan  : A-TTGAAAACAAAGTCCCCCTAAATGATCTGAGTGAAGTAATATCTTTCTCGATTCGCTAACTAGCAGTATATGGTTTAACTAAAATTTT : 9089
Zheng : A-TTGAAAACAAAGTCCCCCTAAATGATCTGAGTGAAGTAAGATCTTTCTCGATTCGCTAACTAGCAGTATATGGTTTAACTAAAATTTT : 9433
Liu   : AATTGAAAACAAAGTCCACCTAAATGATCTGAGTGAAGTAAGATCTTTCTCGATTCGCTAACTAGCAGTATATGGTTTAACTAAAATTTT : 9113
        A TTGAAAACAAAGTCCcCCTAAATGATCTGAGTGAAGTAAgATCTTTCTCGATTCGCTAACTAGCAGTATATGGTTTAACTAAAATTTT

                   *        9560         *        9580         *        9600         *        9620         *
Guan  : AGAACAAGTTAATAATGAAATTCTTAAATAATATAATATCCAGAAATTCTAATTCTTAAATATTTTCTTTTTGCTTATTTAAGAATTAATA : 9179
Zheng : AGAACAAGTTAATAATGAAATTCTTAAATAATATAATATCCAGAAATTCTAATTCTTAAATATTTTCCTTTTGCTTATTTAAGAATTAATA : 9523
Liu   : AGAACAAGTTAATAATGAAATTCTTAAGTAATATAATATCCAGAAATTCTAATTCTTAAATATTTTCCTTTTGCTTATTTAAGAAAAATA : 9203
        AGAACAAGTTAATAATGAAATTCTTAAaTAATATAATATCCAGAAATTCTAATTCTTAAATATTTTCcTTTTGCTTATTTAAGAAtAATA

                 9640         *        9660         *        9680         *        9700         *        9720
Guan  : AAAACAACGTTAAAATGTATGCAAACAACATTTAAATTCACGACAA--------TCGAATCCATGAATGGATAAAAAGTCTTTCTTCGTG : 9261
Zheng : AAAACAACGTTAAAATGTATGCAAACAACATTTAAATTCACGACAAAAGATAAATCGAATCCATGAATGGATAAAAAGTCTTTCTTCGTG : 9613
Liu   : AAAACAACGTTAAAATGTATGCAAACAACATTTAAATTCACGACAAAAGATAAATCGAATCCATGAATGGATAAAAAGTCTTTCTTCGTG : 9293
        AAAACAACGTTAAAATGTATGCAAACAACATTTAAATTCACGACAAaagataaaTCGAATCCATGAATGGATAAAAAGTCTTTCTTCGTG

                   *        9740         *        9760         *        9780         *        9800         *
Guan  : GAGTTAAAATGGGCACATCGTTATAATATGGTTTTTCACACGAAATACACACAGACAAACTTCATTTCGTGACCATTCCTGTTGACATTG : 9351
Zheng : GAGTTAAAATGGGCACATCGTTATAATATGGTTTTTCACACGAAATACACACAGACAAACTTCATTTCGTGACCATTCCTGTTGACATTG : 9703
Liu   : GAGTTAAAATGGGCACATCGTTATAATATGGTTTTTCACACGAAATACACACAGACAAACTTCATTTCGTGACCATTCCTGTTGACATTG : 9383
        GAGTTAAAATGGGCACATCGTTATAATATGGTTTTTCACACGAAATACACACAGACAAACTTCATTTCGTGACCATTCCTGTTGACATTG

                 9820         *        9840         *        9860         *        9880         *        9900
Guan  : TGCTGCGATATTTTATCGATTGTTTACATTGACTTTTATTATTTTTAAAATTAATAATAAAATTAATAATTTTTGTTGAATGTTAAGCTT : 9441
Zheng : TGCTGCGATATTTTATCGATTGTTTACATTGACTTTTATTATTTTTAAAATTAATAATAAAATTAATAATTTTTGTTGAATGTTAAGCTT : 9793
Liu   : TGTTGCGATATTTTATCGATTGTTTACATTGACTTTTATTATTTTTAAAATTAATGATAAAATTAATAATTTTTGTTGAATGTTAAGCTT : 9473
        TGcTGCGATATTTTATCGATTGTTTACATTGACTTTTATTATTTTTAAAATTAATaATAAAATTAATAATTTTTGTTGAATGTTAAGCTT

                   *        9920         *        9940         *        9960         *        9980         *
Guan  : GCACGACTTTTCTATATAAAGGTGAATACAATTAGAAATGGATTACCAAGCCGCACCATGATAGTGTTGATGATTTGTCTTGCCCTGCCT : 9531
Zheng : GCACGACTTTTCTATATAAAGGTGAATACTATTAGAAATGGAATACCAAGCCGCACCATGATAGTGTTGATGATTTGTCTTGCCCTGCCT : 9883
Liu   : GCACGACTTTTCTATATAAAGGTGAATACTATTAGAAATGGAATACCAAGCCGCACCATGATAGTGTTGATGATTTGTCTTGCCCTGCCT : 9563
        GCACGACTTTTCTATATAAAGGTGAATACtATTAGAAATGGAaTACCAAGCCGCACCATGATAGTGTTGATGATTTGTCTTGCCCTGCCT
```

```
        10000         *       10020         *       10040         *       10060         *       10080
Guan  : GCGGGTTTTGCAAATGGTATGGTATATTATTTTCAATCGCATGTTAATGCTGATTAATGCCGTTATTTGATAATCAATTATCGTGTTTTT :  9621
Zheng : GTGGGTTTTGCAAATGGTATGGTATATTATTTTCAATCGCATGTTAATGCTGATTAATGCCGTTATTTGATAATTAATTATCGTGTTTTT :  9973
Liu   : GTGGGTTTTGCAAATGGTATGGTATATTATTTTCAATCGCATGTTAATGCTGATTAATGCCGTTATTTGATAATCAATTATCGTGTTTTT :  9653
        GtGGGTTTTGCAAATGGTATGGTATATTATTTTCAATCGCATGTTAATGCTGATTAATGCCGTTATTTGATAATcAATTATCGTGTTTTT

              *       10100         *       10120         *       10140         *       10160         *
Guan  : GTTTCCTATTAACCTTTTAAATGCCAAGAAATTAGAATTTACGGATGATTACGGCCACTACAATCATCCTTTAATCTTCAATATTGATCC :  9711
Zheng : GTTTCCTATTAACCTTTTAAATGCCAAGAAATTAGAATTTACGGATGATTACGGCCACTATAATCATCCTTTAATCTTCAATATTGATCC : 10063
Liu   : GTTTCCTATTAACCTTTTAAATGCCAAGAAATTATAATTTACGGATGATTACGGCCACTACAATCATCCTTTAATCTTCAATATTGATCC :  9743
        GTTTCCTATTAACCTTTTAAATGCCAAGAAATTAgAATTTACGGATGATTACGGCCACTAcAATCATCCTTTAATCTTCAATATTGATCC

        10180         *       10200         *       10220         *       10240         *       10260
Guan  : TTTAGGGTATTTTTTGAAATAATAAGTTTTGTTATTAATCCAGATTAACAATAACTAATTGTAAAGATGGTAAGCTTAAATCATTTTTCA :  9801
Zheng : TTTAGGGTATTTTTTGAAATAATAAGTTTTGTTATTAATCCAGATTAACAATAACTAATTG---------TAAGCTTAAATCATTTTTCA : 10144
Liu   : TTTAGGGTATTTTTTGAAATAATAAGTTTTGTTATTAATCCAGATTAACAATAACTAATTGTAAAGATGGTAAGCTTAAATCATTTTTAA :  9833
        TTTAGGGTATTTTTTGAAATAATAAGTTTTGTTATTAATCCAGATTAACAATAACTAATTGtaaagatggTAAGCTTAAATCATTTTTcA

              *       10280         *       10300         *       10320         *       10340         *
Guan  : ATCATCAGTATAATTATCTATGTGTCATATGAATGCTATAAATGACATTTTATGTTATACATACATGTATGTACTTCATATAAATATAGA :  9891
Zheng : ATCATCAGTATAATTATCTATGTGTCATATGAATGCTATAAATGACATTTTATGTTATACATACATGTATGTACTTCATATACATATAGA : 10234
Liu   : ATCATCAGTATAATTATCTATGTGTCATATGAATGCTATAAATGACATTTTATGTTATACATACATGTATGTACTTCATATACATATAGA :  9923
        ATCATCAGTATAATTATCTATGTGTCATATGAATGCTATAAATGACATTTTATGTTATACATACATGTATGTACTTCATATAcATATAGA

        10360         *       10380         *       10400         *       10420         *       10440
Guan  : ATAATAATCAATATTATTAGTATTATTAAATCGATGACTATGTTGTGTTTGTTTACATGAAGCCGATTTACAGTCTTGTCGCTCGGTGAA :  9981
Zheng : ATAATAATCAATATTATTAGTATTATTAAATCGATGACTATGTTGTGTTTGTTTACATGAAGCCGATTTACAGTCTTGTCGCTCGGTGAA : 10324
Liu   : ATAATAATCAATATTATTAGTACTATTAAATCGATGACTATGTTGTGTTTGTTTACATGAAGCCGATTTACAGTCTTGTCGCTCGGTGAA : 10013
        ATAATAATCAATATTATTAGTAtTATTAAATCGATGACTATGTTGTGTTTGTTTACATGAAGCCGATTTACAGTCTTGTCGCTCGGTGAA

              *       10460         *       10480         *       10500         *       10520         *
Guan  : ATTCGATTGGAGGAATCAACCTATTACTGCTTGCAAATGCGGGGTAATAATAAACTTAAATAATAATTAAAATTCAAAATAATATTTGGT : 10071
Zheng : ATTCGATTGGAGGAATCAACCTATTACTGCTTGCAAATGCGGGGTAATAATAAACTTAAATAATAATTAAAATTCAAAATAATATTTGGT : 10414
Liu   : ATTTGATTGGAGGAATCAACCTATTACTGCTTGCAAATGCGGGGTAATAATAAACTTAAATAATAATTAAAATTCAAAATAATATTTGGT : 10103
        ATTcGATTGGAGGAATCAACCTATTACTGCTTGCAAATGCGGGGTAATAATAAACTTAAATAATAATTAAAATTCAAAATAATATTTGGT

        10540         *       10560         *       10580         *       10600         *       10620
Guan  : CTTTTTTTTTAATGACAAAAGCAACTTGATTTTTTTCC-GATGTTCACAATTAATTAATAACATAATTTATAATAAGTATAAGTATCTAA : 10160
Zheng : CTTTTTTTTTAATGACAAAAGCAACTTGATTTTTTTCC-GATGTTCACAATTAATTAATAACATAATTTATAATAAGTATAAGTATCTAA : 10503
Liu   : CTTTTTTTTTAATGACAAA-GCAACTTGATTTTTTTCCCGATGTTCACAATTAAATAATAACATAATTTATAATAAGTATAAGTATCTAA : 10192
        CTTTTTTTTTAATGACAAAaGCAACTTGATTTTTTTCC GATGTTCACAATTAAtTAATAACATAATTTATAATAAGTATAAGTATCTAA

              *       10640         *       10660         *       10680         *       10700         *
Guan  : TAATAATAAGTATAAGTTTATCTAAGATCATGTCATAGATTTATAATCCTCTCACAGGAAAGGCAATGCCACGAGTACGAAGAGTGCGTG : 10250
Zheng : TAATAATAAGTATAAGTTTATCTAAGATCATGTCATAGATTTATAATCCTCTCACAGGAAAGGCAATGCCACGAGTACGAAGAGTGCGTG : 10593
Liu   : TAATAATAAGTATAAGTTTATCTAAGATCATGTCATTGATTTATAATCCTCTCACAGGAAAGGCAATGCCACGAGTACGAAGAGTGCGTG : 10282
        TAATAATAAGTATAAGTTTATCTAAGATCATGTCATaGATTTATAATCCTCTCACAGGAAAGGCAATGCCACGAGTACGAAGAGTGCGTG

        10720         *       10740         *       10760         *       10780         *       10800
Guan  : ATGCCGAGAGGCGATTCCTTTGATTACTGCAGACAGGGTATTACTAATTCCTAATTCCTAATTTTATCTTTCTGCTTTTTTAATTATTTG : 10340
Zheng : ATGCCGAGAGGCGATTCCTTTGATTACTGCAGACAGGGTATTACTAATTCCTAATTCCTAATTTTATCTTTCTGCTTTTTTAATTATTTG : 10683
Liu   : ATGCCGAGAGGCGATTCCTTTGATTACTGCAGACAGGGTATTACTAATTCCTAATTCCTAATTTTATCTTTCTGCTTTTTTAATTATTTG : 10372
        ATGCCGAGAGGCGATTCCTTTGATTACTGCAGACAGGGTATTACTAATTCCTAATTCCTAATTTTATCTTTCTGCTTTTTTAATTATTTG

              *       10820         *       10840         *       10860         *       10880         *
Guan  : GTGAAAGTAATTCATGTATTTGAAAAAAAA-TAACAACAAAGAAATAATTTAAATGTGAATATAACATTTTACTAACGAATATAGTCATT : 10429
Zheng : GTGAAAGTAATTCATGTATTTGAAAAAAAAATAACAACAAAGAAATAATTTAAATGTGAATATAACATTTTACTAACGAATATAGTCATT : 10773
Liu   : GTGAAAGTAATTCATGTATTTGAAAAAAAA-TAACAACAAAGAAATAATTTAAATGTGAATATAACATTTAACTGACGAATATAGTCATT : 10461
        GTGAAAGTAATTCATGTATTTGAAAAAAAA TAACAACAAAGAAATAATTTAAATGTGAATATAACATTTtACTaACGAATATAGTCATT

        10900         *       10920         *       10940         *       10960         *       10980
Guan  : CGGATATGATAGAGATTAAATGATAATACTATTTTATTTTAAAACCAGTTCATCCTTTTTGTACTGGCGATACGGTTAAGGACGACTGGG : 10519
Zheng : CGGATATGATAGAGATTAAATGATAATACTATTTTATTTTAAAACCAGTTCATCCTTTTTGTACTGGCGATACGGTTAAGGACGACTGGG : 10863
Liu   : CGGAGATGATAGAGATTAAATAATAATACTATTTTATTTCAAAAACAGTTCATCCTTTTTGTACTGGCGATACGGTTAAGGACGACTGGG : 10551
        CGGAtATGATAGAGATTAAATgATAATACTATTTTATTTtAAAAcCAGTTCATCCTTTTTGTACTGGCGATACGGTTAAGGACGACTGGG

              *       11000         *       11020         *       11040         *       11060         *
Guan  : ATTGCTTTTGTGGAGTAAGCCAAATTTAAGTACAATATATTTAAATATTACATATTTAATAATTAAATAAATCTAATAGTTATGCCAGCA : 10609
Zheng : ATTGCTTTTGTGGAGTAAGCCAAATTTAAGTA-AATATATTTAAATATTACATATTTAATAATTAAATAAATCTAATAGTTATGCCAGCA : 10952
Liu   : ACTGCTTTTGCGGAGTAAGCCAAATTTAAGTAAAATAT--TCAAATATTACATATTTAATAATTAAATAAATCTAATAGTTATGCCAGCA : 10639
        AtTGCTTTTGtGGAGTAAGCCAAATTTAAGTA AATATatTtAAATATTACATATTTAATAATTAAATAAATCTAATAGTTATGCCAGCA

        11080         *       11100         *       11120         *       11140         *       11160
Guan  : AACATGTACTCT--TCTTC-TTCTCATTATTACTATTAATATTATTATTATTATTATTATTATCATTATTATTACTATTATTATTATTAC : 10696
Zheng : AACATGTACTTTCTTCTTCATTATTACTATTATTATTAATATTATTATTATTATTATTATTATTATTATTATTATTATTATTATTATTAT : 11042
Liu   : AACATGTACTTT--TCTTC-TTCTCATTATTACTATTAATATAAATATTATTATTATTATTATCATTATTATTACTATTATTATTATTAC : 10726
        AACATGTACTtT  TCTTC TTcTcAtTATTAcTATTAATATtAtTATTATTATTATTATTATcATTATTATTAcTATTATTATTATTAc

              *       11180         *       11200         *       11220         *       11240         *
Guan  : TATTGT--------------------------------TATTATTTTTATTATCGATGTCATTAATTTTAAACAAAATGAAAAAATGGTA : 10754
Zheng : TATTATCATTATTATTACTATTATTATTATTACTATTTTATTATTTTTATTATCGATGTCATTAATTTTAAACAAAATGAAAAAATGGTA : 11132
Liu   : TATTGT--------------------------------TATTATTTTTATTATCGATGTCATTAATTTTAAACAAAATGAAAAAATGGTA : 10784
        TATTgT                                TATTATTTTTATTATCGATGTCATTAATTTTAAACAAAATGAAAAAATGGTA
```

```
              11260         *       11280         *       11300         *       11320         *       11340
Guan  : TTTGTCTTTCAGGATAGCCGATGCCGTCACACGGAAGACTGCAAGAAAGTTGAAGGCGTCTTACAATGCGTCGAAAGTGAGTCTCTCTAA : 10844
Zheng : TTTGTCTTTCAGGATAGCCGATGCCGTCACACGGAAGACTGCAAGAAAGTTGAAGGCGTCTTACAATGCGTCGAAAGTGAGTCTCTCTAA : 11222
Liu   : TTTGTCTTTCAGGATAGACGATGCCGTCACACGGAAGACTGCAAGAAAGTTGAAGGCGTCTTACAATGCGTCGAAAGTGAGTCTCTCTAA : 10874
        TTTGTCTTTCAGGATAGcCGATGCCGTCACACGGAAGACTGCAAGAAAGTTGAAGGCGTCTTACAATGCGTCGAAAGTGAGTCTCTCTAA

                *       11360         *       11380         *       11400         *       11420         *
Guan  : GTTGTTTCAATGTAAAATAAAAAAAATAATAAAATGAAAATAACAAAAAATTAAATATATTTATAATACACAAAATATGCATAATACAT : 10934
Zheng : GTTGTTTCAATGTAAAATTAAAAAAATAATAAAATGAAAATAACAAAAAATTAAATATATTTATAATACACAAAATATGCATAATACAT : 11312
Liu   : GTTGTTTCAATGTAAAATAAAAAA--ATTATAAAATAAAAATAACAAAAAATTAAATATATTTACAATACATAA--TATACATA-TACAT : 10959
        GTTGTTTCAATGTAAAATaAAAAAaaATaATAAAATgAAAATAACAAAAAATTAAATATATTTAtAATACAcAAaaTATgCATAaTACAT

              11440         *       11460         *       11480         *       11500         *       11520
Guan  : ATAGGTAATACAATATAATATATGTATGATGCTATCCATATATTAGTTATTATTATATATTATTAATCCGGTTAAGTAGTTAAGCGGTTAAG : 11024
Zheng : ATAGGTAATACAAAATAATATATATATGATGCTATCCATATATTAGTTATTATTATATATTATTAATCCGGTTAAGTAGTTAAGCGGTTAAG : 11402
Liu   : ATAGGTAATATAATATAATATATGTATGTTACTATCCATATATTAGTTATTATTATATATTATTAATCCGGTTACGTAGTTAAGCGGTTAAG : 11049
        ATAGGTAATAcAAtATAATATATgTATGaTgCTATCCATATATTAGTTATTATTATATATTATTAATCCGGTTAaGTAGTTAAGCGGTTAAG

                *       11540         *       11560         *       11580         *       11600         *
Guan  : TAGTAACATAGATATTCAATGCAGGACCAGAGGATTGCACAGTCGTGGAAAGAGACGAATTTGGATTACCAATCGGTCCTTGCATTTGTG : 11114
Zheng : TAGTAACATAGATATTCAATGCAGGACCAGAGGATTGCACAGTCGTGGAAAGAGACGAATTTGGATTACCAATCGGTCCTTGCATTTGTG : 11492
Liu   : TAGTAAGATAGATATTCAATGCAGGACCAGAGGATTGCACAGTCGTGGAGAGAGACGAATTTGGATTACCAATAGGTCCTTGCATTTGTG : 11139
        TAGTAAcATAGATATTCAATGCAGGACCAGAGGATTGCACAGTCGTGGAaAGAGACGAATTTGGATTACCAATcGGTCCTTGCATTTGTG

              11620         *       11640         *       11660         *       11680         *       11700
Guan  : CCTGGGTTAGTCACTGATCAGTTTTAATAATATGTATGCTCAGTAAAACGTGCTCATAAGCAATTTCTCAATTTGGTATCACTCACGACA : 11204
Zheng : CCTGGGTTAGTCACTGATCAGTTTTAATAATATGTATGCTCAGTAAAACGTGCTCATAAGCAATTTCTCAATTTGGTATCACTCACGACA : 11582
Liu   : CCTGGGTTAGTCACTGATCAGTTTTAATAATATGTATGCTCATTAAAACGTGCTCATAAGCAATTTCTCAATTTGGTATCACTCACGACA : 11229
        CCTGGGTTAGTCACTGATCAGTTTTAATAATATGTATGCTCAgTAAAACGTGCTCATAAGCAATTTCTCAATTTGGTATCACTCACGACA

                *       11720         *       11740         *       11760         *       11780         *
Guan  : TTTATATAAATTATGAAGCTGATAAATAGTTTAAAAAATACTATGAGGCCATGCTATTATTTATCAAGGGTAAGGAGTGTGCAGATAACG : 11294
Zheng : TTTATATAAATTATGAAGCTGATAAATAGTTTAAAAAATACTATGAGGCCATGCTATTATTTATCAAGGGTAAGGAGTGTGCAGATAACG : 11672
Liu   : TTTATATAAATTATGAAGCTGATAAATAGTTTAGAAAATACTACGAGGCCATGCTATTATTTATCAAGGGTAAGGAGTGTGCAGATAACG : 11319
        TTTATATAAATTATGAAGCTGATAAATAGTTTAaAAAATACTAtGAGGCCATGCTATTATTTATCAAGGGTAAGGAGTGTGCAGATAACG

              11800         *       11820         *       11840         *       11860         *       11880
Guan  : AATATTGTCAAATTGCTAGAGGAGACAATATCAACCAATGCTTAGGAAGTAAGTTTTGAGTAAATTGCGTAATGAAAAATAATGTCATCA : 11384
Zheng : AATATTGTCAAATTGCTAGAGGAGACAATATCAACCAATGCTTAGGAAGTAAGTTTTGAGTAAATTGCGTAATGAAGAATAATGTCATCA : 11762
Liu   : AATATTGTCAAATTGCTAGAGGAGACAATATCAACCAATGCTTAGGAAGTAAGTTTTGAGAAAATTGCGTAATGAAGAATAATGTCATCA : 11409
        AATATTGTCAAATTGCTAGAGGAGACAATATCAACCAATGCTTAGGAAGTAAGTTTTGAGtAAATTGCGTAATGAAgAATAATGTCATCA

                *       11900         *       11920         *       11940         *       11960         *
Guan  : TTTAATGTTGCCCTTTTAAAATCAGAATATTGCACTGGTAGGGCTAATGATCCACCAAGGTGCATTTGTGGAGTAAGTGAAATTTTATGT : 11474
Zheng : TTTAATGTTGCCCTTTTAAAATCAGAATATTGCACTGGTAGGGCTAATGATCCACCAAGGTGCATTTGTGGAGTAAGTGAAATTTTATGT : 11852
Liu   : TTTAATGTTGCCCTTTTAAAATCAGAATATTGCACTGGTAGGGCTAATGATCCACCAAGGTGCATTTGTGGAGTAAGTGAAATTTTATGT : 11499
        TTTAATGTTGCCCTTTTAAAATCAGAATATTGCACTGGTAGGGCTAATGATCCACCAAGGTGCATTTGTGGAGTAAGTGAAATTTTATGT

              11980         *       12000         *       12020         *       12040         *       12060
Guan  : AATATCTATTATTATAATTATTATTAATATAATAATATATAATAATAATATAATAATATAATATAATAAT-------------TATTA : 11550
Zheng : A-TATCTATTATTATAATTATTATTAATATAATAATA-ATAATAATA--ATAATAATA-TAATATAATATAA-------------TATTA : 11924
Liu   : AATATCTATTATTATAATTATTATTAATATAATAATA-ATAATAATATAATAATAATAATAATATAATAATATAATAATAATATTTATTA : 11588
        AaTATCTATTATTATAATTATTATTAATATAATAATA ATAATAATA  ATAATAATAaTAATATAATAata             TATTA

                *       12080         *       12100         *       12120         *       12140         *
Guan  : TTATTA--TTATGAGAAAATAATGAAATATCTTT-TGTATTTCAGGGTAAGCAATGCGACCATAATCAATACTGCAGGAACTTTAATGGC : 11637
Zheng : ATATAAAATAATATAAATATCTTTTATTATTATTATGTATTTCAGGGTAAGCAATGCGACCATAATCAATACTGCAGGAACTTTAATGGC : 12014
Liu   : TTATTA--TTATGAGAAAATAATGAAATATCTTT-TGTATTTCAGGGTAAGCAATGCGACCATAATCAATACTGCAGGAACTTTAATGGC : 11675
        tTATtA  TtATgagAAaATaaTgaAaTATctTT TGTATTTCAGGGTAAGCAATGCGACCATAATCAATACTGCAGGAACTTTAATGGC

              12160         *       12180         *       12200         *       12220         *       12240
Guan  : AAATTGCAATGTACTAAAAGTGAGTTTTTCTTAAGTGTTTTATATAGTGAGACACGTTATTTTAAAGTTGTTAGTACCACGATTTATAAA : 11727
Zheng : AATTTGCAATGTACTAAAAGTGAGTTTCTCTTAAGTGTTTTTTATTGTGAGACACGTTATTTTAAAGTT-TTAGTACCA---TTTATAAA : 12100
Liu   : AAATTGCAATGTACTAAAAGTGAGTTTCTCTTAAGTGTTTTTTATTGTGAGACACGTTATTTTAAAGTTGTTAGTACCACGATTTATAAA : 11765
        AAaTTGCAATGTACTAAAAGTGAGTTTcTCTTAAGTGTTTTtTATtGTGAGACACGTTATTTTAAAGTTgTTAGTACCAcgaTTTATAAA

                *       12260         *       12280         *       12300         *       12320         *
Guan  : CAAATTCTATGTATAATATTGTAATAATAATAATAATTATAATAGTAATTATTATTATACTATTACATTAGTAAAGTATTTCATCAAACG : 11817
Zheng : CAAATTCTATGTATAATATT------------------------------------ATACTATTACATTAGTAAAATATTTCATCAAACG : 12154
Liu   : CAAATTCTATGTATAATATG------------------------------------ATACTATTACATTAGTAACGTATTTCATCAAACG : 11819
        CAAATTCTATGTATAATATt                                    ATACTATTACATTAGTAAagTATTTCATCAAACG

              12340         *       12360         *       12380         *       12400         *       12420
Guan  : GACCTTCTGTTATGACGATAGCTCTAATATTTGTTATATAAAGTTATGTGCACATTGTTCTTAGGTTATTAATTTAGTTAAATTTAATTT : 11907
Zheng : GACCTTCTGTTATGACGATAGCTCTAATATTTGTTATATAAAGTTATGTGCACATTGTTCTTAGGTTATTAATTTAGTTAAATTTAATTT : 12244
Liu   : GACCTTCTGTTATGACGATAGCTCTAATATTTGTTATATAAAGTTATGTGCACATTGTTCTTAGGTTATTAATTTAGTTAAATTTAATTT : 11909
        GACCTTCTGTTATGACGATAGCTCTAATATTTGTTATATAAAGTTATGTGCACATTGTTCTTAGGTTATTAATTTAGTTAAATTTAATTT

                *       12440         *       12460         *       12480         *       12500         *
Guan  : GTAACAACTAAGTTAA-TTTGGCTATTAAAGGTTAATCCGCTGTAAACTATTATTTTATTATTATTAAATGCAGTACCGAATCGTTGCGA : 11996
Zheng : GTAACAACTAAGTTAAGTTTGGCTATTAAAGGTTAATCCGTTGTAAACTATTATTAATTATTATTAAATGCAGTACCGAATCGTTGCGA : 12334
Liu   : GTAACAACTAAGTTAA-TTTGGCTATTAAAGGTTAATCCGCTGTAAACTATTATTAATTATTATTAAATGCAGTACCGAATCGTTGCGA : 11998
        GTAACAACTAAGTTAA TTTGGCTATTAAAGGTTAATCCGcTGTAAACTATTATTaTATTATTATTAAATGCAGTACCGAATCGTTGCGA
```

```
             12520         *       12540         *       12560         *       12580         *       12600
Guan  : AAAGATGGAGAAGGACAAACTCGGTCGCCCCATTAGTCCTTGCGTTTGTGGAATGGTTAGTCGTGGATCAAATTTGGTCATCGGAATGCT : 12086
Zheng : AAAGATGGATAAGGACAAACTCGGTCGCCCCATTAGTCCTTGCGTTTGTGGAATGGTTAGTCGTGGATCAAATTTGGTCATCGGAATGCT : 12424
Liu   : AAAGATGGAGAAGGACAAACTCGGTCGCCCCATTAGTCCTTGCGTTTGTGGAATGGTTAGTCGTGGATCAAATTTGGTCATCGGAATGCT : 12088
        AAAGATGGAgAAGGACAAACTCGGTCGCCCCATTAGTCCTTGCGTTTGTGGAATGGTTAGTCGTGGATCAAATTTGGTCATCGGAATGCT

                *       12620         *       12640         *       12660         *       12680         *
Guan  : CTTACAAATCGTAATAATATTTTATTTAATTAGGCGATTTGATATTTAACATTCAAAACGGTGTAAAATTTAGAA-TTAATTTAGAAGAC : 12175
Zheng : CTTACAAATCGTAATAATATTTTATTTAATTAGGCGATTTGATATTTAACATTCAAAACGGTGTAAAATTTAGAAATTAATTTAGAAGAC : 12514
Liu   : CTTACAAATCGTAATAATATTTTATTTAATTAGGCGATTTGATATTTAACATTCAAAACGGTGTAAAATTTAGAA-TTAATTTAGAAGAC : 12177
        CTTACAAATCGTAATAATATTTTATTTAATTAGGCGATTTGATATTTAACATTCAAAACGGTGTAAAATTTAGAA TTAATTTAGAAGAC

             12700         *       12720         *       12740         *       12760         *       12780
Guan  : CTTTATGAATCGCAATCATTATTTACCAAGGGTCAGGTGTGTGAAAAGCACGAATACTGTGATATGATACATGAAGATGCCACCAACTT : 12265
Zheng : CTTTATGAATCGCAATCATTATTTACTAAGGGTCAGGTTTGTGAAAAGCACGAATACTGTAATATAACACATGAAGGTGCAACCAAAGT : 12604
Liu   : CTTTATGAATCGCAATCATTATTTACCAAGGGTCAGGTGTGTGAAAAGCACGAATACTGTGATATGATACATGAAGATGCCACCAACTT : 12267
        CTTTATGAATCGCAATCATTATTTACcAAGGGTCAGGTgTGTGAAAAGCACGAATACTGTgATATGAtACATGAAGaTGCcACCAActT

                *       12800         *       12820         *       12840         *       12860         *
Guan  : TTGCTTACCAAGTATGTTTCGTTTAGATTATGAAA-TAAACAATTATACGAAGATTTAGCGATTAATTCTCACATTGATTTATATTAACG : 12354
Zheng : TTGCTTACCAAGTATGTTTCGTTTAGATTATGAAAATAAACAATTATACCAAGATTTAGCGATTAATTCTCACATTGATTTATATTA--- : 12691
Liu   : TTGCTTACCAAGTATGTTTCGTTTAGATTATGAAA-TAAACAATTATACGAAGATTTAGCGATTAATTCTCACATTGATTTATATTAACG : 12356
        TTGCTTACCAAGTATGTTTCGTTTAGATTATGAAA TAAACAATTATACgAAGATTTAGCGATTAATTCTCACATTGATTTATATTAacg

             12880         *       12900         *       12920         *       12940         *       12960
Guan  : CTTTGATTGTCCCTAGAATATCTCCCGTGCAAACCGGGGTCAATTGTTCAGAAGAAATGTTTATGTGGTGTTAGTAATTA------TTAC : 12438
Zheng : -TTTGATTGTCCTTAGAATATCTCCCGTGCAAACTAAAGTCAATTGTAGAGAAGAAATGTTTATGTGGTGTTAGTAACTAATACTGTTAT : 12780
Liu   : CTTTGATTGTCCCTAGAATATCTCCCGTGCAAACCGGGGTCAATTGTTCAGAAGAAATGTTTATGTGGTGTTAGTAATTA------TTAC : 12440
        cTTTGATTGTCCcTAGAATATCTCCCGTGCAAACcgggGTCAATTGTtcAGAAGAAATGTTTATGTGGTGTTAGTAAtTA      TTAc

                *       12980         *       13000         *       13020         *       13040         *
Guan  : TAAC--AAACATTCTTTAGTATTGCCGATTAAGAGATAATTATTACATTAAAGTACCTAAAAATAACATTAAAATAAAGTTATCCATATC : 12526
Zheng : TAACTTAACTATTCTTTAGTATTTCCGATTAAGAGATTATTATTATATTAAAGTACCTAAAAATAATATTAAAATAAAGTTAGCCATATG : 12870
Liu   : TAAC--AAACATTCTTTAGTATTGCCGATTAAGAGATAATTATTACATTAAAGTACCTAAAAATAACATTAAAATAAAGTTATCCATATC : 12528
        TAAC  AAacATTCTTTAGTATTgCCGATTAAGAGATaATTATTAcATTAAAGTACCTAAAAATAAcATTAAAATAAAGTTAtCCATATc

             13060         *       13080         *       13100         *       13120         *       13140
Guan  : TTATACATGTAGAATAAGTCGTGCAACAAAGGAAAGAGATGCAAATCGATTAAAGGCGAAGAAAAAATGCTTGGAAGGTTGTATACACATC : 12616
Zheng : TTATACATGTAGAATAAGTCGTGCAACAAAGGAAAGAAATGCAAATTGGTTAAAGGCGAAGAAAAAATGCTTGGAAGGTTGTATGCACACC : 12960
Liu   : TTATACATGTAGAATAAGTCGTGCAACAAAGGAAAGAGATGCAAATCGATTAAAGGCGAAGAAAAAATGCTTGGAAGGTTGTATACACATC : 12618
        TTATACATGTAGAATAAGTCGTGCAACAAAGGAAAGAgATGCAAATcGaTTAAAGGCGAAGAAAAAATGCTTGGAAGGTTGTATaCACAtC

                *       13160         *       13180         *       13200         *       13220         *
Guan  : TTCGTTTCTACATTGATGTTGTTAAAACATGAATTTGAGTTTTGGAATGATTATTACTATTAATAATGCTATTAATATTATTAGATTAAT : 12706
Zheng : TTCGTTTCTACATTGACGTTTTTGGAAATATGAATTTGAGTTTTGGAATTATTATTACTATTAATTATGCTATTAATATTATTA------- : 13043
Liu   : TTCGTTTCTACATTGATGTTGTTGAAACATGAATTTGAGTTTTGGAATGATTATTACTATTAATAATGCTATTAATATTATTAGATTAAT : 12708
        TTCGTTTCTACATTGAtGTTgTTgAAAcATGAATTTGAGTTTTGGAATgATTATTACTATTAATaATGCTATTAATATTATTAgattaat

             13240         *       13260         *       13280         *       13300         *       13320
Guan  : ATTATTATTAATTCTATTATTTTCAGCTGGCAAAAAGAAGAAGGTATCCAAAAAATCCAGCAAAAATGCCTAATAAAGCGGACTATCGAA : 12796
Zheng : -------TTAATTCTATTATTTTCAGTTAGCAAAAAGAAGAAGATATCCAAAAAATCCAGCAAAAATTCCGAATAAAGAGGAACATCGAA : 13126
Liu   : ATTATTATTAATTCTATTATTTTCAGTTAGCAAAAAGAAGAAGATATCCAAAAAATCCAGCAAAACTTCCGAATAAAGAGGAACATCGAA : 12798
        attattaTTAATTCTATTATTTTCAGtTaGCAAAAAGAAGAAGaTATCCAAAAAATCCAGCAAAAaTtCCgAATAAAGaGGAacATCGAA

                *       13340         *       13360         *       13380         *       13400         *
Guan  : CCTCATGAAACATCTCATTTTATGAACATTTCATAACTTGTTGCTGTTCTGAATTTAATTAACCATACGTTAATAAATTACTTATGTTGT : 12886
Zheng : CCTGATGAAACATCTCATTTTATGAACATTTCATAACATGTTGCTGTTCTGAATTTAATTAACCATACGTTAATAAATTACTTATGTTGT : 13216
Liu   : CCTGATGAAACATCTCATTTTATGAACATTTCATAATTTGTTGCTGTTCTGAATTTGATTAACCATACGTTAATAAATTACTTATGTTGT : 12888
        CCTgATGAAACATCTCATTTTATGAACATTTCATAActTGTTGCTGTTCTGAATTTaATTAACCATACGTTAATAAATTACTTATGTTGT

             13420         *       13440         *       13460         *       13480         *       13500
Guan  : ATGGCTAACATCAAATTAATTTTGACATTT-ATATATATCTGAATGAATGATTTGTTTTTTAAAGACAGTCGGTTTCTTGGAATAATACA : 12975
Zheng : ATGACTAACATCAAATTAATTTTGACATTTTATATATATCTGAATGAATGATTTGTTTTTTAAAGACAGTCGGTTTCTTGGAATAATACA : 13306
Liu   : ATGGCTAACATCAAATTAATTTTGACATTTTATATATATCTGAATGAATGATTTGTTTTTTAAAGACAGTCGGTTTCTTGGAATAATACA : 12978
        ATGgCTAACATCAAATTAATTTTGACATTTtATATATATCTGAATGAATGATTTGTTTTTTAAAGACAGTCGGTTTCTTGGAATAATACA

                *       13520         *       13540         *       13560         *       13580         *
Guan  : TTTAAGTCGTCAGAGAGAAAGAAGAGTTTTGTTAGATATTAATATTTATCAA---------TTATAAAATAAATGCGGATAGTTGGAATA : 13056
Zheng : TTTAAGTCATTAGAGAGAA-GAATAGTTTTGTTAGATATTCTGATTTCTCAAAATCGAGAATTATAAAATAAATGCTGATAGTTGGAATA : 13395
Liu   : TTTAAGTCGTCAGAGAGAAGGAAGAGTTTTGTTAGATATTCTGATTTCTCAAAATCGAGAATTATAAAATAAATGCCGATAGTTGGAATA : 13068
        TTTAAGTCgTcAGAGAGAA GAAgAGTTTTGTTAGATATTctgATTTcTCAAaatcgagaaTTATAAAATAAATGC GATAGTTGGAATA

             13600         *       13620         *       13640         *       13660         *       13680
Guan  : GAATTTAAGGTGTTTTTTTGAGTAGAAATTTTATATTTATCCATTATCAATTTAAAGATATTGATAGATATTAATAAACATGGGGAGCAG : 13146
Zheng : GAATTTAAGGTGTTTTTTTGAGTAGAAATTTTATATTTATCCATTATCAATTTAAAGTTATTGATAGATATTAATAAACATGGGGAGCAG : 13485
Liu   : GAATTTAAGGTGTTTTTTTGAGTAGAAATTTTATATTTATCCATTATCAATTTAAAGATATTGATAGATATTAATAAACATGGGGAGCAG : 13158
        GAATTTAAGGTGTTTTTTTGAGTAGAAATTTTATATTTATCCATTATCAATTTAAAGaTATTGATAGATATTAATAAACATGGGGAGCAG

                *       13700         *       13720         *       13740         *       13760         *
Guan  : CTGTTAATATGGATCTGTTAATAAATGATTTTTCTGTCTTTGGCCAGACAAAGATATCAAAACGCATGTGAGTCCGTGCTTTTTGAAACA : 13236
Zheng : CTGTTAATATGGATCTGTTAATAAATGATTTTTCTGTCTTTGGCCAGA-AAAGATATCAAAACGC--GTGAGTCCGTGCTTTTTGAAACA : 13572
Liu   : CTGTTAATATGGATCTGTTAATAAATGATTTTTCTGTCTTTGGCCAGA-AAAGATATCAAAACGCATGTGAGTCCGTGCTTTTTGAAACA : 13247
        CTGTTAATATGGATCTGTTAATAAATGATTTTTCTGTCTTTGGCCAGA AAAGATATCAAAACGCatGTGAGTCCGTGCTTTTTGAAACA
```

```
                13780         *     13800         *     13820         *     13840         *     13860
Guan  : AATTCACACGGAATACTTTTAAAATAACGATTATTTTCTATTGAATGTTTGTATTGAAATACATTAAGTTCACTATTTAGGGAGTCTTTC : 13326
Zheng : AATTCACACGGAATACTTTTAAAATAACGATTATTTTCTATTGAATGTTTGTATTGAAATACATTAAGTTCACTATTTAGGGAGTCTTTC : 13662
Liu   : TATTCACACGGAATACTTTTAAAATAACGATTATTTTCTATTGAATGTTTGTATTGAAATACATTAAGTTCACAATTTAGGGAGTCTTTC : 13337
        aATTCACACGGAATACTTTTAAAATAACGATTATTTTCTATTGAATGTTTGTATTGAAATACATTAAGTTCACtATTTAGGGAGTCTTTC

                  *     13880         *     13900         *     13920         *     13940         *
Guan  : TTTAATTTATTCAAGTGTCTTGTCCAAACAATGAACAAGAATTGCTTCTCTATTTAAATGCATAACTTCCTGCTGTACACCGAACTAGAA : 13416
Zheng : TTTAATTTATTCAAGTGTCTTGTCCAAACAATGAACAAGAATTGCTTCTCTATTTAAATGCATAACTTCCTGCTTTACACCGAACTAGAA : 13752
Liu   : TTTAATTTATTCTAGTGTCTTGTCCAAACAATGAACAAGAATTGCTTCTCTATTTAAGTGCATAACTTCGGGCTTTACACCGAACTAGAA : 13427
        TTTAATTTATTCaAGTGTCTTGTCCAAACAATGAACAAGAATTGCTTCTCTATTTAAaTGCATAACTTCctGCTtTACACCGAACTAGAA

                13960         *     13980         *     14000         *     14020         *     14040
Guan  : TCAACTGAATTCATCATGTTAGTTCTGATAATTTGTCTTCTACTGTCCTTTTCATTGGACATCAATGCAAAGAGTGAGAAACGATTCATT : 13506
Zheng : TCAACTGAATTCATCATGTTAGTTCTGATAATTTGTCTTCTACTCTCCTTTTCATTGGACATCAATGCAAAGAGTGAGAAACGATTCATT : 13842
Liu   : TCAACTGAATTCATCATGTTAGTTCTGATAATTTGTCTTCTACTGTCCTTTTCATTGGACATCAATGCAAAGAGTGAGAAACGATTCATT : 13517
        TCAACTGAATTCATCATGTTAGTTCTGATAATTTGTCTTCTACTgTCCTTTTCATTGGACATCAATGCAAAGAGTGAGAAACGATTCATT

                  *     14060         *     14080         *     14100         *     14120         *
Guan  : TGAGATGATATATCAAAACATATGCATTATTTATTATATTAATATTTTAGGCGAATTTTCATTAACTAAATTCTTGCATAACAGATAAAT : 13596
Zheng : TGAGATGATATATCAAAACATATGCATTATTTATTATATTAATATTTTAGGCGAATTTTCATTAACTAAATTCTTGCATAACAGATAAAT : 13932
Liu   : TGAGATGATATATCAAAACATATGCATTATTTATTATATTAATATTTTAGGCGAATT------AACTAAATTCTTGCATAACAGATAAAT : 13601
        TGAGATGATATATCAAAACATATGCATTATTTATTATATTAATATTTTAGGCGAATTttcattAACTAAATTCTTGCATAACAGATAAAT

                14140         *     14160         *     14180         *     14200         *     14220
Guan  : TTAAGTAAATAAATGTAATTAAAAAA-GATTAAAGATAAATTCTTATTTTAGAAATGAAGTAAAATAC---------ATCATGTCTAA-- : 13674
Zheng : TTAAGTAAATAAATGTAATTAAAAAAAGATTAAAGATAAATTCTTATTTTAGAAATGAAGTAAAATATTATTTATGAATCATGTCTAAAA : 14022
Liu   : TTAAGTAAATAAATGTAATTAAAAAA-GATTAAAGATAAATTCTTATTTTAGAAATGAAGTAAAATAC---------ATCATGTCTAA-- : 13679
        TTAAGTAAATAAATGTAATTAAAAAA GATTAAAGATAAATTCTTATTTTAGAAATGAAGTAAAATAc         ATCATGTCTAA

                  *     14240         *     14260         *     14280         *     14300         *
Guan  : -------CCATATA--CAATTAACCAATCTTCAAAATACGTTGATTAGGAACTGGTTATTAATAATTACGCATATTTAATTAGCAATAAA : 13755
Zheng : ATAATGTCTATATATTCAATTAACCAATCTTCAAAATACGTTGATTAGGAACTGGTTATTAATAATTACGCATATTTAATTAGCAATAAA : 14112
Liu   : -------CCATATA--CAATTAACCAATCTTCAAAATACGTTGATTAGGAACTGGTTATTAATAATTACGCATATTTAATTAGCAATAAA : 13760
               CcATATA  CAATTAACCAATCTTCAAAATACGTTGATTAGGAACTGGTTATTAATAATTACGCATATTTAATTAGCAATAAA

                14320         *     14340         *     14360         *     14380         *     14400
Guan  : TATTAATTGAAATTACATCTAAATAGTTATTCGGTTATGAATGCGTTTATTTAAACTTTAGAAGATATACCAGGCTGTCATCCCGAGGTA : 13845
Zheng : TATTAATTGAAATTACATCTAAATAGTTATTCGGTTATGAATGCGTTTATTTAAACTTTAGAAGATATACCAGGCTGTCATCCCGAGGTA : 14202
Liu   : TATTAATTGAAATTACATCTAAATAGTTATTCGGTTATGAATGCGTTTATTTAAACTTTAGAAGATATACCAGGCTGTCATCCCGAGGTA : 13850
        TATTAATTGAAATTACATCTAAATAGTTATTCGGTTATGAATGCGTTTATTTAAACTTTAGAAGATATACCAGGCTGTCATCCCGAGGTA

                  *     14420         *     14440         *     14460         *     14480         *
Guan  : GGATTGCCACCCCCATCTGGAGTTACCTGCATTTGTGGAGTAGGAATATCTTTAAAATAATAATAATAATAATAAT---------TTATC : 13926
Zheng : GGATTGCCACC-CCATCTGGAGTTACCTGCATTTGTGGAGTAGGAATATCTTTAAAATAATAATAATAATAATAAT---------TTATC : 14282
Liu   : GGATTGCCACCTCCATCTGGAGTTACCTGCATTTGTGGAGTAGGAATATCTTTAAAATAATAATAATAATAATAATAATAATAATTTATC : 13940
        GGATTGCCACC CCATCTGGAGTTACCTGCATTTGTGGAGTAGGAATATCTTTAAAATAATAATAATAATAATAAT         TTATC

                14500         *     14520         *     14540         *     14560         *     14580
Guan  : ATGGCTATCTTATTTGAAAAGAAATACGTTTGTTTACAAACAATATAAACCCAAGTTTTATTATTGCGTTTATGAAACATGAAAGGTAAT : 14016
Zheng : ATGGCTATCTTATTTGAAAAGAAATACGTTTGTTTACAAACAACATAAACCCAAGTTTTATTATTGCGTTTATGAAACATGAAAGGTAAT : 14372
Liu   : ATGGCTATCTTATTTGAAAAGAAATACGTTTGTTTACAAACAATATAAACCCAAGTTTTATTATTGCGTTTATGAAACATGAAAGGTAAT : 14030
        ATGGCTATCTTATTTGAAAAGAAATACGTTTGTTTACAAACAAtATAAACCCAAGTTTTATTATTGCGTTTATGAAACATGAAAGGTAAT

                  *     14600         *     14620         *     14640         *     14660         *
Guan  : CTATTGAAATTTAGAAAAGTGTTTTCTATCCATATATTTTATGGTAGGGAATTCATTGATATTAATATTGATATTATTTTATGTATTATT : 14106
Zheng : CTATTGAAATTTAGAAAATTGTTTTCTATCCATATATTTTATGGTAGGGAATTCATTGATATTAATATTGATATTATTTTATGTATTATT : 14462
Liu   : CTATTGAAATTTAGAAAAGTGTTTTCTATCCATACATTTTATGGTAGGGAATTCATTGATATTAATATTGATATTATTTTATGTATTATT : 14120
        CTATTGAAATTTAGAAAAgTGTTTTCTATCCATAtATTTTATGGTAGGGAATTCATTGATATTAATATTGATATTATTTTATGTATTATT

                14680         *     14700         *     14720         *     14740         *     14760
Guan  : ATTAATATAAATATCATTTTAAAAAAATTATATAAATATACATTTGAATAATAAAAAATTATATAAATATAAATTTGAATAATAAAAAATT : 14196
Zheng : ATTAATATAAATATCATTTTAAAAAAATTATATAAATATA-------------------------------AATTTGAATAATAAAAAATT : 14521
Liu   : ATTAATATAAATATCATTTTAAAAAAATTATATAAATATACATTTGAATAATAAAAAATTATATAAATATAAATTTGAATAATAAAAAATT : 14210
        ATTAATATAAATATCATTTTAAAAAAATTATATAAATATAcatttgaataataaaaaattatataaatataAATTTGAATAATAAAAAATT

                  *     14780         *     14800         *     14820         *     14840         *
Guan  : AAAAGAATTCACAATATATTAAGTTTTGCTGTTGTTTTT-GTATCCAGAAAACAGAATGTAGTACCAAAGACAGATGCATTATCGCAAGA : 14285
Zheng : AAAAGAATTCACAATATATTAAGTTTTGCTGTTGTTTTT-GTATCCAGAAAACAGAATGTAGTACCAAAGACAGATGCATTATCGCAAGA : 14610
Liu   : AAAAGAATTCACAATATATTAAGTTTTGCTGTTGTTTTTTGTATCCAGAAAACAGAATGTAGTACCAAAGACAGATGCATTATCGCAAGA : 14300
        AAAAGAATTCACAATATATTAAGTTTTGCTGTTGTTTTT GTATCCAGAAAACAGAATGTAGTACCAAAGACAGATGCATTATCGCAAGA

                14860         *     14880         *     14900         *     14920         *     14940
Guan  : GGTGATAGTAGAAACTACTGCGCTTGATAATAACACTTTCGTATGATTATCGGTTATCAACCTGAATTATGACAATAAAATCCGGAATTT : 14375
Zheng : GGTGATAGTAGAAACTACTGCGCTTGATAATAACACTTTCGTATGATTATCGGTTATCAACCTGAATTATGACAATAAAATCCGGAATTT : 14700
Liu   : GGTGATAGTAGAAACTACTGCGCTTGATAATAACACTTTCGTATGATTATCGGTTATCAACCTGAATTATGACAATAAAATCCGGAATTT : 14390
        GGTGATAGTAGAAACTACTGCGCTTGATAATAACACTTTCGTATGATTATCGGTTATCAACCTGAATTATGACAATAAAATCCGGAATTT

                  *     14960         *     14980         *     15000         *     15020         *
Guan  : AAAGGCAAATTACTTGTGATTTAATATAATATAATAAATTTAATGGAATAAAAAACAATTTATTTCAGTATCGATCTCTTTAGGTACCCT : 14465
Zheng : AAAGGCAAATTACTTGTGATTTAATATAATATAATAAATTTAATGGAATAAAAAACAATTTATTTCAGTATCGATCTCTTTAGGTACCCT : 14790
Liu   : AAAGGCAAATTACTTGTGATTTAATATAATATAATAAATTTAATGGAATAAAAAACAATTTATTTCAGTATCGATCTCTTTAGGTACCCT : 14480
        AAAGGCAAATTACTTGTGATTTAATATAATATAATAAATTTAATGGAATAAAAAACAATTTATTTCAGTATCGATCTCTTTAGGTACCCT
```

```
                15040         *       15060         *       15080         *       15100         *       15120
Guan   : AACGAACTCATCTTAAAAATAAAATTAATTTTAGCCAGGGCTATTCCGTGGACATAATTTTAATTATAAGTGGAAATGATAATTGCTTGA : 14555
Zheng  : AACGAACTCATCTTAAAAATAAAATTCATTTTAGCCAGGGCTATTCCTTGGACATAATTTTAATTATAAGTGGAAATGATAATTGCTTGA : 14880
Liu    : AACGAACTCATCTTAAAAATAAAATTAATTTTAGCCAGGGCTATTCCTTGGACATAATTTTAATTATAAGTGGAAATGATAATTACTTGA : 14570
         AACGAACTCATCTTAAAAATAAAATTaATTTTAGCCAGGGCTATTCCtTGGACATAATTTTAATTATAAGTGGAAATGATAATTgCTTGA

                   *       15140         *       15160         *       15180         *       15200         *
Guan   : AATCACTGCTTATTGATATGCAACCCTTGAGATTGTATAATTATTAAATTTTAACTCATACGACAATCTTTTGTTTTGCATACATTTGAA : 14645
Zheng  : ATTACTTTGATAT-------CAACCCTTGAGATTGTATAATTATTAAATTTTAACTCATACGACAATCTTTTGTTTTGCATACATTTGAA : 14963
Liu    : AATCA-----------------ACCCTTGAGATTGTATAATTATTAAATTTTAACTCATACGACAATCTTTTGTTTTGCATACATTTGAA : 14643
         AaTca t   tat       caACCCTTGAGATTGTATAATTATTAAATTTTAACTCATACGACAATCTTTTGTTTTGCATACATTTGAA

                15220         *       15240         *       15260         *       15280         *       15300
Guan   : ACTGTAGATTTATAAACATTTTTAATATCAAATACGCTAGATAAGGCAGATTTCGCAATGTTATAGAAAAGGGATTTGATTTTATTTAAA : 14735
Zheng  : ACTGTAGATTTATAAACATTTTTAATATCAAATACGCTAGATAAGGCAGATTTCGCAATGTTATAGAAAAGGGATTTGATTTTATTTAAC : 15053
Liu    : ACTGTAGATTTATAAACATTTTTAATGTCAAATACGCTAGATAAGGCAGATTTCGCAATGTTATAGAAAAGGGATTTGATTTTATTTGAC : 14733
         ACTGTAGATTTATAAACATTTTTAATAtCAAATACGCTAGATAAGGCAGATTTCGCAATGTTATAGAAAAGGGATTTGATTTTATTTaAc

                   *       15320         *       15340         *       15360         *       15380         *
Guan   : GCCTTATTTTGTATTTTCAAATCTAAAATACGGTAATTCCATAGAATTTCCACGTGAAAGGTTAATTTGATTCCATTCTGTAGCTAGATG : 14825
Zheng  : GCCTTATTTAGTATTTTCAAATCTAAAATACGGTAATTCCATA-AATTTCCACGTGAAAGGTTAATTTGATTCCATTCTGTAGCTGGATG : 15142
Liu    : GCCTTATTTTGTATTTTCAAATCTAAAATACGGTAATTCCATAGAATTTCCACGTGAAAGTTTAATTTGATTCCATTCTGTAGCTAGATG : 14823
         GCCTTATTTtGTATTTTCAAATCTAAAATACGGTAATTCCATAgAATTTCCACGTGAAAGgTTAATTTGATTCCATTCTGTAGCTaGATG

                15400         *       15420         *       15440         *       15460         *       15480
Guan   : ACCTGTCATCAAGTCACACCCTAGTTTGACCTAGTTTGAGCATATATTTATAAAAAGGAGGAGGTAATGGTAATAACATCCGTATTTCTA : 14915
Zheng  : ACCTGTCATCAAGTCACTCCCTAGTTTGACCT-----GAGCATACATTTATAAAAAGGAGGAGGTAATGGTAATAACATCCGTATTTCTA : 15227
Liu    : ACCTGTCATTAAGTCAATCCCTAGTTTGACCT-----GAGCATACATTTATAAAAAGGAGGAGGTAATGGTAATAACATCCGTATTTCTA : 14908
         ACCTGTCATcAAGTCActCCCTAGTTTGACCT     GAGCATAcATTTATAAAAAGGAGGAGGTAATGGTAATAACATCCGTATTTCTA

                   *       15500         *       15520         *       15540         *       15560         *
Guan   : ATAATTTTACAAAATATATTTAATACTTTTAACAATTCGATATAATACATTTTCATATTTTATTTGATTTCCAGTTTTTTTTTTTTTTGCT : 15005
Zheng  : ATAATTTTACAAAATATATTTAATACTTTTAACAATTCGATATAATACATTTTCATATTTTATTTGATTTCCAGTTTTTTTGTTGTT-GTT : 15316
Liu    : ATAATTTTATAAAATATATTTAATACTTTTAACAATTCGATATAATACATTTTCAGATTTTATTTGATTTCCAGCTTTTTTTT-----GCT : 14993
         ATAATTTTAcAAAATATATTTAATACTTTTAACAATTCGATATAATACATTTTCAtATTTTATTTGATTTCCAGtTTTTTTtTt tt GcT

                15580         *       15600         *       15620         *       15640         *       15660
Guan   : AATCTCAACATGTCGAAACATCGTGTTGAAAAACCAAATGATTAATGATAAATCGAATCCGTGAATCGATAAAAATTCTTCTTTCGTGGA : 15095
Zheng  : AATCTCAACATGTCGAAACATCGTGTTGAAAAACCAAATGATTAATGATAAATCGAATCCGTGAATCGATAAAAATTCTTCTTTCGTGGA : 15406
Liu    : AATCTCTACGTGTCGAAACATCGTGTTGAAAAACCAAATGATTAATGATAAATCGAATCCGTGAATCGATAAAAATTCTTCTTTCGTGGA : 15083
         AATCTCaACaTGTCGAAACATCGTGTTGAAAAACCAAATGATTAATGATAAATCGAATCCGTGAATCGATAAAAATTCTTCTTTCGTGGA

                   *       15680         *       15700         *       15720         *       15740         *
Guan   : GTTAAAATGGGCAGATCGTTATAATATGTTTTTTCACACGAAATACACACAGACAAACTTCATTTCGAGACCATTCCTGTTGACATTGTG : 15185
Zheng  : GTTAAAATGGGCAGATCGTTATAATATGTTTTTTCACACGAAATACACACAGACAAACTTCATTTCGAGACCATTCCTGTTGACATTGTG : 15496
Liu    : GTTAAAATGGGCAGATCGTTATAATATGGTTTTTCACACGAAATACACACAGACAAACTTCATTTCGAGACCATTCCTGTTGACATTGTG : 15173
         GTTAAAATGGGCAGATCGTTATAATATGtTTTTTCACACGAAATACACACAGACAAACTTCATTTCGAGACCATTCCTGTTGACATTGTG

                15760         *       15780         *       15800         *       15820         *       15840
Guan   : CTGCGATATTTTACCGATTGTTTACATTGACTTTTATTATTTTTTTAAAA-TTAATAATAAAATGAATAATCTTCGTCGAATGTTAAGTT : 15274
Zheng  : CTGCGATATTTTACCGATTATTTACATTGACTTTTATTATTTTTTTAAAAATTAATAATAAAATGAATAATCTTCGTCGAATGTTGAGCT : 15586
Liu    : CTGCGATATTTTACCGATTGTTTACATTGACTTTTATTATTTTTTTTAAA-TTAATAATAAAATGAATAATCTTCGTCGAATGTTAAGCT : 15262
         CTGCGATATTTTACCGATTgTTTACATTGACTTTTATTATTTTTTTaAAA TTAATAATAAAATGAATAATCTTCGTCGAATGTTaAGcT

                   *       15860         *       15880         *       15900         *       15920         *
Guan   : CGCATGACTTTTCTATATAAAGGTGAATTCTATTAAGAATGGAATACCAAGCCGCACCATGATAGTGTTGATGATTTGTCTTGCCCTGCC : 15364
Zheng  : CGCATGACTTTTCTATATAAAGGTGAATTCTATTAAGAATGGAATACCAAGCCGCACCATGATAGTGTTGATGATTTGTCTTGCCCTGCC : 15676
Liu    : CGCATGACTTTTCTATATAAAGGTGAATTCTATTAAGAATGGAATACCAAGCCGCACCATGATAGTGTTGATGATTTGTCTTGCCCTGCC : 15352
         CGCATGACTTTTCTATATAAAGGTGAATTCTATTAAGAATGGAATACCAAGCCGCACCATGATAGTGTTGATGATTTGTCTTGCCCTGCC

                15940         *       15960         *       15980         *       16000         *       16020
Guan   : TGTGGGCTTTGCAAGTGGTATGGCATATTATTCAATCGCATGTTAATGCTGATTAATGCCGATAATTGTTATTCAATTAGTGTGTTTTTT : 15454
Zheng  : TGTGGGCTTTGCAAGTGGTATGGCATATTATTCAATCGCATGTTAATGCTGATTAATGCCGATAATTGTTATTCAATTAGTGTGTTTTTT : 15766
Liu    : TGTGGGCTTTGCAAGTGGTATGGCATATTATTCAATCGCATGTTAATGCTGATTAATGCCGATAATTGTTATTCAATTAGTGTGTTTTTT : 15442
         TGTGGGCTTTGCAAGTGGTATGGCATATTATTCAATCGCATGTTAATGCTGATTAATGCCGATAATTGTTATTCAATTAGTGTGTTTTTT

                   *       16040         *       16060         *       16080         *       16100         *
Guan   : GTTTTCTATTAAACTTTTGAATGTCAGGAAATTAGAATTTCAGTGTGACATACATTATATATATAATATATAAATATTAGTTTTGTTACA : 15544
Zheng  : GTTTTCTATTAAACTTTTGAATGTCAGGAAATTAGAATTTCAGTGTGACATACATTATATATATAATATATAAATATTAGTTTTGTTACA : 15856
Liu    : GTTTTCTATTAAACTTTTGAATGTCAGGAAATTAGAATTTCAGTGTGACATACATT---------------------------------- : 15498
         GTTTTCTATTAAACTTTTGAATGTCAGGAAATTAGAATTTCAGTGTGACATACATTatatatataatatataaatattagttttgttaca

                16120         *       16140         *       16160         *       16180         *       16200
Guan   : TTACATTTGTTACATTGTTACATTATTAATTCGCGGTTAAGTAGTAACATTATTAGTCAATGCAGGATCGAAGGATTGCTCAGTCGTGGA : 15634
Zheng  : TTACATTTGTTACATTGTTACATTATTAATTCGCGGTTAAGTAGTAACATTATTAGTCAATGCAGGATCGAAGGATTGCTCAGTCGTGGA : 15946
Liu    : ------------------------------------------------------------------------------------------ :     -
         ttacatttgttacattgttacattattaattcgcggttaagtagtaacattattagtcaatgcaggatcgaaggattgctcagtcgtgga

                   *       16220         *       16240         *       16260         *       16280         *
Guan   : GAGAGACTACTTTGGATTTCCAATTGGTCCTTGCATTTGTGCCTGGGTTAGTCACTGATCAGTTTTGATAATATATCTGCTCATTAAAAA : 15724
Zheng  : GAG--ACTACTTTGGATTTCCAATTGGTCCTTGCATATGTGCCTGGGTTAGTCACTGATCAGTTTTGATAATATGTCTGCTCATTAAAAA : 16034
Liu    : ------------------------------------------------------------------------------------------ :     -
         gag  actactttggatttccaattggtccttgcat tgtgcctgggttagtcactgatcagttttgataatat tctgctcattaaaaa
```

```
             16300         *      16320         *      16340         *      16360         *      16380
Guan  : TGTGCTTATTTGTAAATTTATTATCAGTCACGACATTTATATCATTTTGGAGCTGATAAATAATTTAGAAAATACTATGAGGCCATGCCA : 15814
Zheng : TGTGCTTATGTGTAAATTTATTATCAGTCACGACATTTATATCATTTTGGAGCTGATAAATAATTTAGAAAATACTATGAGGCCATGCCA : 16124
Liu   : ------------------------------------------------------------------------------------------ :     -
        tgtgcttat tgtaaatttattatcagtcacgacatttatatcattttggagctgataaataatttagaaaatactatgaggccatgcca

                 *      16400         *      16420         *      16440         *      16460         *
Guan  : TCATTTATCAAGGGTAAGGAGTGTGGACAAGACGAATATTGTCAAATTGCTAGAGGAGACAACATCAACTATTGTGTGAAAAGTAAGAAC : 15904
Zheng : TTATTTATCAAGGGTAAGGAGTGTGGACAAGACGAATATTGTCAAATTGCTAGAGGAGACAACATCAACTATTGTGTGAAAAGTAAGAAC : 16214
Liu   : ------------------------------------------------------------------------------------------ :     -
        t atttatcaagggtaaggagtgtggacaagacgaatattgtcaaattgctagaggagacaacatcaactattgtgtgaaaagtaagaac

             16480         *      16500         *      16520         *      16540         *      16560
Guan  : CGGATAAATTACGAAATAAGGAATAATGTCATCATTTAATGTTGTCCTTTTATAATCAGAATATTGCACTGGTGACAGGGCTAATGATTC : 15994
Zheng : CGGATAAATTACGGAATAAGGAATAATGTCATCATTTAATGTTGTCCTTTTATAATCAGAATATTGCACTGGTGACAGGGCTAATGATTC : 16304
Liu   : ------------------------------------------------------------------------------------------ :     -
        cggataaattacg aataaggaataatgtcatcatttaatgttgtccttttataatcagaatattgcactggtgacagggctaatgattc

                 *      16580         *      16600         *      16620         *      16640         *
Guan  : AGCAAGGTGCATTTGTGGAGTAAGTGAAACTTTATGTAATATCTATTATTATTATTAT------------------------------GA : 16054
Zheng : AGCAAGGTGCATTTGTGGAGTAAGTGAAATTTTATGTAATATCTATTATTATTATTATTATTATTATTATTATTATTATTATTATTATGA : 16394
Liu   : ------------------------------------------------------------------------------------------ :     -
        agcaaggtgcatttgtggagtaagtgaaa tttatgtaatatctattattattattat                              ga

             16660         *      16680         *      16700         *      16720         *      16740
Guan  : GAAAATAATGAAATATCTTTTGTATTTCAGGATAAGCGATGCGGGTATAACGAGGACTGCAAGAAAAATGGAAATAAATTGCAATGTGTC : 16144
Zheng : GAAAATAATGAAATATCTTTTGTATTTCAGGATAAGCGATGCGGGTATAACGAGGACTGCAAGAAAAATGGAAACAAATTGCAATGTGTC : 16484
Liu   : ------------------------------------------------------------------------------------------ :     -
        gaaaataatgaaatatcttttgtatttcaggataagcgatgcgggtataacgaggactgcaagaaaaatggaaa aaattgcaatgtgtc

                 *      16760         *      16780         *      16800         *      16820         *
Guan  : GAATGTCAGTATCATTTAGATGTTTTTATTGTGTTTCAAAATTGTTGCTAACACGATTGTAAAACAATATGTTATTATATGTACACAAGG : 16234
Zheng : GAAAGTCAGTATCATTTAGATGTTTTTATTGTGTTTCAAAATTGTTGCTAACACGATTGTAAAACAATATGTTATTATATGTACACAAGG : 16574
Liu   : ------------------------------------------------------------------------------------------ :     -
        gaa gtcagtatcatttagatgtttttattgtgtttcaaaattgttgctaacacgattgtaaaacaatatgttattatatgtacacaagg

             16840         *      16860         *      16880         *      16900         *      16920
Guan  : ATCATATTTCACAGACGAGTCCGTGTCTTTTATTAAATCTCGTTCGTACGGGATAATAGCGAGTCGTGATTCTTTCTGTTGACTTTGTGA : 16324
Zheng : ATCATATTTCACAGACGAGTCCGTGTCTTTTTTTAACTCTCGGTCGTACGGGATAATAGCGAGTCGTGATTCTTTCTGTTGACTTTGTGA : 16664
Liu   : ------------------------------------------------------------------------------------------ :     -
        atcatatttcacagacgagtccgtgtctttt ttaa tctcg tcgtacgggataatagcgagtcgtgattctttctgttgactttgtga

                 *      16940         *      16960         *      16980         *      17000         *
Guan  : AAATTAATATAGACTGAAAATTAATTCATTCGATAATTGCTTTGAGCTTTGTATTTACTCATGTACCTTGATCAATCAACGGACGTGAGT : 16414
Zheng : AAATTAATATAGACTGAAAATTAATTCATTCGATAATTGCTTTGAGCTTTGTATTTACTCATGTACCTTGATCAATCAACGGACGTGAGT : 16754
Liu   : ------------------------------------------------------------------------------------------ :     -
        aaattaatatagactgaaaattaattcattcgataattgctttgagctttgtatttactcatgtaccttgatcaatcaacggacgtgagt

             17020         *      17040         *      17060         *      17080         *      17100
Guan  : GATCTCAACTCGAAGAAAGTTTAAACAAAGTTTAGACATGTTTCTTCCCAATGATAGGCTTTACAAAAAATTTGTATTAGTATAACATTTT : 16504
Zheng : GATCTCAACTCGAAGAAAGTTTAAACAAAGTTTAGACATGTTTCTTCCCAATGATAGGCTTTACAAAAAATTTGTATTAGTATAACATTTT : 16844
Liu   : ------------------------------------------------------------------------------------------ :     -
        gatctcaactcgaagaaagtttaaacaaagtttagacatgtttcttcccaatgataggctttacaaaaaatttgtattagtataacatttt

                 *      17120         *      17140         *      17160         *      17180         *
Guan  : TT-AAAATGTTTATTTTTGAAAACATTCTCGCAGATGATTTAAGATGCTTTAGGAGCATGTCTTTTAGAAAATGTGAAACACGTCACATG : 16593
Zheng : TTTAAAATGTTTATTTTTGAAAACATTCTCGCAGATGATTTAAGATGCTTTAGGAGCATGTCTTTTAGAAAATGTGAAACACGTCACATG : 16934
Liu   : ------------------------------------------------------------------------------------------ :     -
        tt aaaatgtttatttttgaaaacattctcgcagatgatttaagatgctttaggagcatgtcttttagaaaatgtgaaacacgtcacatg

             17200         *      17220         *      17240         *      17260         *      17280
Guan  : GACTGTCGATTTTTATCATTTTATTTACCAAACATTTCCTTATAAATTCTTTACTAACTGGTGTACGAATTCTTTGGATGTCGCAGGAAG : 16683
Zheng : GACTGTCGATTTTTATCATTTTATTTACCAAACATTTCCTTATAAATTCTTTACTAACTGGTGTACGAATTCTTTGGATGTCGCAGGAAG : 17024
Liu   : ------------------------------------------------------------------------------------------ :     -
        gactgtcgatttttatcattttatttaccaaacatttccttataaattctttactaactggtgtacgaattctttggatgtcgcaggaag

                 *      17300         *      17320         *      17340         *      17360         *
Guan  : ACATCATTGATTTATTTCAGAATGGACAGGTGGGAAAGGTGGCTAAAGTTTTAATAACATATTAGATTTTACTGAAAGCTACAAGATATC : 16773
Zheng : ACATCATTGATTTATTTCAGAATGGACAGGTGGGAAAGGTGGCTAAAGTTTTAATAACATATTAGATTTTACTGAAAGCTACAAGATATC : 17114
Liu   : ------------------------------------------------------------------------------------------ :     -
        acatcattgatttatttcagaatggacaggtgggaaaggtggctaaagttttaataacatattagattttactgaaagctacaagatatc

             17380         *      17400         *      17420         *      17440         *      17460
Guan  : TAGGTATAAAGATATGCTAATTTGTTATGGTCGAAAAGATGAAATTTTGACAAAATACACATAAATAGATAAAGTTGAGATCACATAACA : 16863
Zheng : TAGGTATAAAGATATGCTAATTTGTTATGGTCGAAAAGATGAAATTTTGACAAAATACACATAAATAGATAAAGTTGAGATCACATAACA : 17204
Liu   : ------------------------------------------------------------------------------------------ :     -
        taggtataaagatatgctaatttgttatggtcgaaaagatgaaattttgacaaaatacacataaatagataaagttgagatcacataaca

                 *      17480         *      17500         *      17520         *      17540         *
Guan  : TTAATATTAATTGCAGAACCTCAGCATTGTTCACATGTGGAGAAAGATGAATTCGGAATACCCATTGGTCCTTGTATTTGTTATGTTAGT : 16953
Zheng : TTAATATTAATTGCAGAACCTCAGCATTGTTCACATGTGGAGAAAGATGAATTCGGAATACCCATTGGTCCTTGTATTTGTTATGTTAGT : 17294
Liu   : ------------------------------------------------------------------------------------------ :     -
        ttaatattaattgcagaacctcagcattgttcacatgtggagaaagatgaattcggaatacccattggtccttgtatttgttatgttagt
```

```
           17560         *      17580         *      17600         *      17620         *      17640
Guan  : CTTGATCAAGTACTTTACTCGTTAAAAACGTGCTTGTTTGTAATTTGTCAATTTGTTAACATTCATGACATTTGTATAACTGTAAAGTTT : 17043
Zheng : CTTGATCAAGTACTTTACTCGTTAAAAACGTGCTTGTT-GTAATTTGTCAATTTGTTAACATTCATGACATTTGTATAACTGTAAAGTTT : 17383
Liu   : ------------------------------------------------------------------------------------------ :     -
        cttgatcaagtactttactcgttaaaaacgtgcttgtt gtaatttgtcaatttgttaacattcatgacatttgtataactgtaaagttt

             *      17660         *      17680         *      17700         *      17720         *
Guan  : AATTAATTAAAAGGAGGCGCTTCGTTCTTTATCCGCTCATCCATCCATCCATCCATCCATCCATTAATTCATCTACGCATTCATCCATCT : 17133
Zheng : AATTAATTAAAAGGAGGCGCTTCGTTCTTTATCCGCTCAT----------------CCAACCATTAATTCATCTACGCATTCATCCATCT : 17457
Liu   : ------------------------------------------------------------------------------------------ :     -
        aattaattaaaaggaggcgcttcgttctttatccgctcat                cca ccattaattcatctacgcattcatccatct

           17740         *      17760         *      17780         *      17800         *      17820
Guan  : TATCCTTCCTTCCATCTATACATCCATTCAACCAACCAACCACCCATCCACCTACCTAGTAAACCAATTTACACAATGGTTTACAAAACA : 17223
Zheng : TATCCTTCCTTCCATCTATACATCCATTCAACCAACCAACCACCCATCCACCTACCTAGTAAACCAATTTACACAATGGTTTACAAAACA : 17547
Liu   : ------------------------------------------------------------------------------------------ :     -
        tatccttccttccatctatacatccattcaaccaaccaaccacccatccacctacctagtaaaccaatttacacaatggtttacaaaaca

             *      17840         *      17860         *      17880         *      17900         *
Guan  : TTGTGTTGAAATCGTACTATTATTTACCAAGCATCTCGAGTGTGAAGAGAACGAATATTGTAATGTTACCGGAGGAATGTACTTCAGCTT : 17313
Zheng : TTGTGTTGAAATCGTACTATTATTTACCAAGCATCTCGAGTGTGAAGAGAACGAATATTGTAATGTTACCGGAGGAATGTACTTCAGCTT : 17637
Liu   : ------------------------TACCAAGTATCTCGAGTGTGAAGAGAACGAATATTGTAATGTTACCGGAGGAATGTACTTCAGCTT : 15564
        ttgtgttgaaatcgtactattattTACCAAGcATCTCGAGTGTGAAGAGAACGAATATTGTAATGTTACCGGAGGAATGTACTTCAGCTT

           17920         *      17940         *      17960         *      17980         *      18000
Guan  : TTGTGAACGATGTAAGTTCCAAGTAAATTTCGAAATAAACATCATTTAATGAATAATTTTCACATTTATTCCTCCGCTAATATTTAATAT : 17403
Zheng : TTGTGAACGATGTAAGTTCCAAGTAAATTTCGAAATAAACATCATTTAATGAATAATTTTCACATTTATTCCTCCGCTAATATTTAATAT : 17727
Liu   : TTGCGAACGATGTAAGTTCCAAGTAAATTTCGAAATAAACATCATTTAATGAATAATTTTCACATTTATTCCTCCGCTAATATTTAATAT : 15654
        TTGtGAACGATGTAAGTTCCAAGTAAATTTCGAAATAAACATCATTTAATGAATAATTTTCACATTTATTCCTCCGCTAATATTTAATAT

             *      18020         *      18040         *      18060         *      18080         *
Guan  : AATTAAGTGAATATTTAATTTAATGCAATTATTCAATTTTCGTAAAAAAATAGAGAAATAACTTAAATGTAAATGTCAGATTAAAATGATT : 17493
Zheng : AATTAAGTGAATATTTAATTTAATGCAATTATTCAATTTTCGTAAAAAAATAGAGAAATAACTTAAATGTAAATGTCAGATTAAAATGATT : 17817
Liu   : AATTAAGTGAATATTTAATTTAATGCAATTATTCAATTTTCGTAAAAAAATAGAGAAATAACTTAAATTTAAATGTCAGATTAAAATGATT : 15744
        AATTAAGTGAATATTTAATTTAATGCAATTATTCAATTTTCGTAAAAAAATAGAGAAATAACTTAAATgTAAATGTCAGATTAAAATGATT

           18100         *      18120         *      18140         *      18160         *      18180
Guan  : AACCTTAAAACACAAACGTTTTCAAATCAGTGTATTGTACGGGTAACCCGGCTGAAGATTCTCCATTGTGCATATGTAAAGTCAGTAACA : 17583
Zheng : AACCTTAAAACACAAACGTTTTCAAATCAGTGTATTGTACGGGTAACCCGGCTGAAGATTCTCCATTGTGCATATGTAAAGTCAGTAACA : 17907
Liu   : AACCTTAAAACACAAACGTTTTCAAATCAGTGTATTGTGCGGGTAACCCGGCTGAAGATTCTCCATTGTGCATATGTAAAGTCAGTAACA : 15834
        AACCTTAAAACACAAACGTTTTCAAATCAGTGTATTGTaCGGGTAACCCGGCTGAAGATTCTCCATTGTGCATATGTAAAGTCAGTAACA

             *      18200         *      18220         *      18240         *      18260         *
Guan  : TTTTATAATAACACTAATAATTACTGTTATAAATTATACAATTCTAAATTAATAATAATATTTATGAGGCTAATTATGACGAAAAAAATGAA : 17673
Zheng : TTTTATCTAACACTAATAATTACTGTTATAAATTATACAATTCTAAATTAATAATAATATTTATAAGGATAATTATGACGAAAAAAATGAA : 17997
Liu   : TTTTATCTAATACTAATAATTACTGTTATAAATTATACAATTCTAAATTAATAATAATATTTATAAGGATAATTATGACGAAAAAAATGAA : 15924
        TTTTATcTAAcACTAATAATTACTGTTATAAATTATACAATTCTAAATTAATAATAATATTTATaAGGaTAATTATGACGAAAAAAATGAA

           18280         *      18300         *      18320         *      18340         *      18360
Guan  : GAAATATCTTTTTGTCTTACAGGATAAGCGATGCAAGCATAATCAATACTGCAGGAACTTTAATGGCAAGATGCAATGTACTAAAAGTGA : 17763
Zheng : GAAATATCTTTT-GTCTTGCAGGATAAGCGATGCAAGCATAATCAATACTGCAGGAACTTTAATGGCAAGATGCAATGTACTAAAAGTGA : 18086
Liu   : GAAATATCTTTTTGTCTTGCAGGATAAGCGATGCGACCATAATCAATACTGCAGGAACTTTAATGGCAAGATGCAATGTACTAAAAGTGA : 16014
        GAAATATCTTTTtGTCTTgCAGGATAAGCGATGCaAgCATAATCAATACTGCAGGAACTTTAATGGCAAGATGCAATGTACTAAAAGTGA

             *      18380         *      18400         *      18420         *      18440         *
Guan  : GTTTCTCTTAAGTGTTTTTTATTGTGAGGAACATTATTTTAAGGTTGTTAATATCATTGATAGCTTTAAAATAATATATATTTCTATGTA : 17853
Zheng : GTTTCTCTTAG-TGTTTTTTATTGTGAGGAACATTATTTTAAGGTTGTTAATATCATTGATAGCTTTAAAATAATATATATTTCTATGTA : 18175
Liu   : GTTTCTCTTAGCTGTTTTTTATTGTGAGGAACATTATTTTAAGGTTGTTAATATCATTGATAGCTTTAAAATAATATATATTTCTATGTA : 16104
        GTTTCTCTTAg TGTTTTTTATTGTGAGGAACATTATTTTAAGGTTGTTAATATCATTGATAGCTTTAAAATAATATATATTTCTATGTA

           18460         *      18480         *      18500         *      18520         *      18540
Guan  : TTGTAATAATAATAAATATCATATGTAGGTATTAATAATAATAACAATAATATTATAATAACAATAATGCTAATAATAATGATGATGTAT : 17943
Zheng : TTGTAATAATAATAAATATCATATGTAAGTATTAATAATAATAACAATAATATTATAATAACAATGATGCTAATAATAATGATGATGTAT : 18265
Liu   : TTGTAATAATAATAAATATCATATGTAGGTATTAATAATAATAACAATAATATTATAATAACAATAATGCTAATAATAATGATGATGTAT : 16194
        TTGTAATAATAATAAATATCATATGTAgGTATTAATAATAATAACAATAATATTATAATAACAATaATGCTAATAATAATGATGATGTAT

             *      18560         *      18580         *      18600         *      18620         *
Guan  : TTTATCAAGCGCATTAATAGAAGGTATGACTAATGCTCTAATATGTTATGTAAAATTAAGTTCATATTGCTATTAGATTATCAGTTTAGT : 18033
Zheng : TTTATCAAGCGGATTAATAGAAGGTATGACTAATGCTCTA---TGTTATGTAAAATTAAGTTCATATCGCTATTAGATTATCAGTTTAGT : 18352
Liu   : TTTATCAAGCGGATTAATAGAAGGTATGACTAATGCTCTA---TGTTATGTAAAATTAAGTTCACATCGCTATTAGATTATCAGTTTAGT : 16281
        TTTATCAAGCGgATTAATAGAAGGTATGACTAATGCTCTA   TGTTATGTAAAATTAAGTTCAtATcGCTATTAGATTATCAGTTTAGT

           18640         *      18660         *      18680         *      18700         *      18720
Guan  : CAAACCGATGTTGTACAGCCATGTTAATTTTTTAGCCTGCTAACAGTTAATATGCTGTTAAGTATTATTATGTTATTATTATTAAATGCA : 18123
Zheng : CAAACCGATGTTGTACAACCATGTTAATTTTTTAGCCTGCTAACAGTTAATATGCTGTTAAGTATTATTATGTTATTATTATTAAATGCA : 18442
Liu   : CAAACCGATGTTGTACAACCATGTTAATTTTTTAGCCTGCTAACAGTTAATATGCTGTTAAGTATTATTATGTTATTATTATTAAATGCA : 16371
        CAAACCGATGTTGTACAaCCATGTTAATTTTTTAGCCTGCTAACAGTTAATATGCTGTTAAGTATTATTATGTTATTATTATTAAATGCA

             *      18740         *      18760         *      18780         *      18800         *
Guan  : GGACCGGAGAGTTGCGAAAATGTGCGAAAGGACAAACTCAATCGCCCCATTAGTCCTTGCGTTTGTGGAATGGTTAGTCGTCGATAAATT : 18213
Zheng : GGACCGGAGAGTTGCGAAAATGTGCGAAAGGACAAACTCAATCGCCCCATTAGTCCTTGCGTTTGTGGAATGGTTAGTCGTTGATAAATT : 18532
Liu   : GGACCGGAGAGTTGCGAAAATGTGCGAAAGGACAAACTCAATCGCCCCATTAGTCCTTGCGTTTGTGGAATGGTTAGTCGTCGATAAATT : 16461
        GGACCGGAGAGTTGCGAAAATGTGCGAAAGGACAAACTCAATCGCCCCATTAGTCCTTGCGTTTGTGGAATGGTTAGTCGTcGATAAATT
```

```
                18820         *       18840         *       18860         *       18880         *       18900
Guan  : TTGGTCATTTTTAAAATTGTACTCAAATTTTAATTAATTTCTCAATTTATTATAATTTTAAAGGCTGTAAAGTTCAGAATTGGTTTAGAA : 18303
Zheng : T-GGTCATTTTTAAAATTGTACTCAAATTTTAATTAATTTCTCAATTTATTATAATTTTAAACGCTGTAAAGTTCAGAATTGGTTTAGAA : 18621
Liu   : TTGGTCATTTTTAAAATTGTACTCAAATTTTAATTAATTTCTCAATTTATTATAATTTTAAACGCTGTAAAGTTCAGAATTGGTTTAGAA : 16551
        TtGGTCATTTTTAAAATTGTACTCAAATTTTAATTAATTTCTCAATTTATTATAATTTTAAAcGCTGTAAAGTTCAGAATTGGTTTAGAA

                  *       18920         *       18940         *       18960         *       18980         *
Guan  : GATATTTATGATTACACAATCATTATTTACCAAGGGTCGGGTTTGTGAAAAGCACGAATACTGTAATATGACACATGAAGATGCAACCAA : 18393
Zheng : GATATTTATGATTTCACAATCATTATTTACCAAGGGTCGGGTTTGTGAAAAGCACGAATACTGTAATATGACACATGAAGATGCAACCAA : 18711
Liu   : GATATTTATGATTTCACAATCATTATTTACCAAGGGTCGGGTTTGTGAAAAGCACGAATACTGTAATATGACACATGAAGATGCAACCAA : 16641
        GATATTTATGATTtCACAATCATTATTTACCAAGGGTCGGGTTTGTGAAAAGCACGAATACTGTAATATGACACATGAAGATGCAACCAA

                19000         *       19020         *       19040         *       19060         *       19080
Guan  : CGTTTGCTTACCAAGTACGTTTCGTTTAGATTATGAAATAAACAATTACATAAAGATTTATGGATTAATTTTCACATTGATTCATATTAA : 18483
Zheng : CGTTTGCTTACCAAGTACGTTTCGTTTAGATTATGAAATAAACAATTACATAAAGATTTATGGATTAATTTTCACATTGATTCATATTAA : 18801
Liu   : CGTTTGCTTACCAAGTACGTTTCGTTTAGATTATGAAATAAACAATTACATAAAGATTTATGGATTAATTTTCACATTGATTCATATTAA : 16731
        CGTTTGCTTACCAAGTACGTTTCGTTTAGATTATGAAATAAACAATTACATAAAGATTTATGGATTAATTTTCACATTGATTCATATTAA

                  *       19100         *       19120         *       19140         *       19160         *
Guan  : CGCTTTGATTTTACTTAGAATATCTCCCGTGCAAACTGGGACCAATTGTTCAGAAGAAATGTTTATGTGGTGTTAGTAACTATTACTGAC : 18573
Zheng : CGCTTTGATTTTACCTAGAATATCTCCCGTGCAAACTGGGACCAATTGTTCAGAAGAAATGTTTATGTGGTGTTAGTAATTATTACTGAC : 18891
Liu   : CGCTTTGATTTTACCTAGAATATCTCCCGTGCAAACTGGGACCAATTGTTCAGAAGAAATGTTTATGTGGTGTTAGTAACTATTACTGAC : 16821
        CGCTTTGATTTTACcTAGAATATCTCCCGTGCAAACTGGGACCAATTGTTCAGAAGAAATGTTTATGTGGTGTTAGTAAcTATTACTGAC

                19180         *       19200         *       19220         *       19240         *       19260
Guan  : AGACATTCTTTTGTATTTCAGATTAAGAGATATTTATTACATTAAAGTACCTAAAAATAACATTAAAATAAAGTTAGCCATATGTTATAC : 18663
Zheng : AGACATTCTTTTGTATTTCAGATTAAGAGATATTTATTACATTAAAGTACCTAAAAATAACATTAAAATAAAGTTAGCCATATGTTATAC : 18981
Liu   : AGACATTCTTTTGTATTTCAGATTAAGAGATATTTATTACATTCAAGTACCTATAAATAACATTAAAATAAAGTTATCCATATCTTATAC : 16911
        AGACATTCTTTTGTATTTCAGATTAAGAGATATTTATTACATTaAAGTACCTAaAAATAACATTAAAATAAAGTTAgCCATATgTTATAC

                  *       19280         *       19300         *       19320         *       19340         *
Guan  : ATGTAGAATAAGTCGTGCAACAAAGGAAAGAAATGCAAATCGATTAAAGGCGAAGCAAAATGCTTGAAAGGTTGTATACACATCTTCGTT : 18753
Zheng : ATGTAGAATAAGTCGTGCAACAAAGGAAAGAAATGCAAATCGATTAAAGGCGAAGCAAAATGCTTGAAAGGTTGTATACACATCTTCGTT : 19071
Liu   : ATGTAGAATAAGTCGTGCAACAAAGGAAAGAAATGCAAATCGATTAAAGGCGAAGCAAAATGCTTGAAAGGTTGTATGCACATCTTCGTT : 17001
        ATGTAGAATAAGTCGTGCAACAAAGGAAAGAAATGCAAATCGATTAAAGGCGAAGCAAAATGCTTGAAAGGTTGTATaCACATCTTCGTT

                19360         *       19380         *       19400         *       19420         *       19440
Guan  : TCTACATTGATATTGTTGAAATATGAATTTGAGTTTTGGAATGATTATCACTATTAATAATGCTACTAATATTATTAGATTAATATTATT : 18843
Zheng : TCTACATTGATATTGTTGAAATATGAATTAGAGTTTTGGATTGATTATCACTATTAATAATGCTACTAATATTATTAGATTAATATTATT : 19161
Liu   : TCTACATTGATATTGTTGAAATATGAATTTGAGTTTTGGAATGATTATCACTATTAATAATGCTACTAATATTATTAGATTAATATTATT : 17091
        TCTACATTGATATTGTTGAAATATGAATTtGAGTTTTGGAaTGATTATCACTATTAATAATGCTACTAATATTATTAGATTAATATTATT

                  *       19460         *       19480         *
Guan  : ATTAATTCTATTATTTTCAGTTAGCAAAAAGAAGAAGATATACAAAAATGCCGAATAA : 18901
Zheng : ATTAATTCTATTATTTTCAGTTAGCAAAAAGAAGAAGATATACAAAAATGCCGAATAA : 19219
Liu   : ATTAATTCTATTATTTTCAGTTAGCAAAAAGAAGAAGATATACAAAAATGCCGAATAA : 17149
        ATTAATTCTATTATTTTCAGTTAGCAAAAAGAAGAAGATATACAAAAATGCCGAATAA
```
