# Supplementary material for: Monomeric and Oligomeric Decorsins of the Asian Medicinal Leech Hirudinaria manillensis
Source: Int J Mol Sci. 2025 Nov 14;26(22):11017. doi: 10.3390/ijms262211017 (PMC12651989; doi:10.3390/ijms262211017)
Supplement: Supplementary file 1 [file ijms-26-11017-s001.zip › File S2.pdf]

Figure S2A. Multiple sequence alignments of putative decorsin Hman_DV1 genes derived from the genome data of *H. manillensis* provided by Guan et al. (2020), Zheng et al. (2023) and Liu et al. (2023), respectively. The exons are labeled in green and the introns are labeled in red. Start and stop codons are marked in bold, the cysteine codons are marked in bold and yellow and the KGD motif encoding codons are marked in cyan and bold

```
                       *        20         *        40         *        60         *        80         *
Guan_DV1   : ATGTTAGTTCTGATAATTTGTCTTCTACTGTCCTTTTCATTGGACGTCGATGCAAATAGTTCAAAGAGTGAGTTAATACGATACATTTTG :   90
Zheng_DV1  : ATGTTAGTTCTGATAATTTGTCTTCTACTGTCATTTTCATTGGACGTCGATGCAAATAGTTCAAAGAGTGAGTTAATACGATTCATTTTG :   90
Liu_DV1    : ATGTTAGTTCTGATAATTTGTCTTCTACTGTCCTTTTCATTGGACGTCGATGCAAATAGTTCAAAGAGTGAGTTAATACGATTCATTTTG :   90
             ATGTTAGTTCTGATAATTTGTCTTCTACTGTCcTTTTCATTGGACGTCGATGCAAATAGTTCAAAGAGTGAGTTAATACGATtCATTTTG

                  100         *       120         *       140         *       160         *       180
Guan_DV1   : AGAAGATATATCAAAACATGTGTATTATATTTGTAATAGTTTTCAATTGATAATTTAAACGAAGTTCTTGTATAACAGAGAAATTTAGA :  180
Zheng_DV1  : AGAAGATATATCAAAACATGTGTATTATATTTGTAATAGTTTTCAATTGATAATTTAAACGAAGTTCTTGTATAACAGAGAAATTTAGA :  180
Liu_DV1    : AGATGATATATCAAAACATGTGTATTATATTTGTAATAGTTTTCAATTGATAATTTAAACGAAGTTCTTGTATAACAGAGAAATTTAGA :  180
             AGAaGATATATCAAAACATGTGTATTATATTTGTAATAGTTTTCAATTGATAATTTAAACGAAGTTCTTGTATAACAGAGAAATTTAGA

                       *       200         *       220         *       240         *       260         *
Guan_DV1   : TAAACATACTTAAATAAAGATTAAAAATAAATACTTATTTTTCAAATAAAATTCTTTCAATATATTAGTTATTGTTATATAATTAAGCAG :  270
Zheng_DV1  : TAAACATACTTAAATAAAGATTAAAAATAAATACTTATTTTTCAAATAAAGTGCTTTCAATATATTAGTTATTGTTATATAATTAAGCAG :  270
Liu_DV1    : TAAACATACTTAAATAAAGATTAAAAATAAATACTTATTTTTCAAATAAAATGCTTTCAATATATTAGTTATTGTTATATAATTAAGCAG :  270
             TAAACATACTTAAATAAAGATTAAAAATAAATACTTATTTTTCAAATAAAaTgCTTTCAATATATTAGTTATTGTTATATAATTAAGCAG

                  280         *       300         *       320         *       340         *       360
Guan_DV1   : TAGTAGCATTAATATTCAATGTAGGATCACAGCCATGTGAATTAGTGGATAGTGACGAATTTGGATTACCAGTCCCTCCTTGTATTTGTG :  360
Zheng_DV1  : TAGTATCATTAATATTCAATGTAGGATCACAGCCATGTGGATTAGTGGATAGTGACGAATTTGGATTACCAGTCCCTCCTTGTATTTGTG :  360
Liu_DV1    : TAGTAGCATTAATATTCAATGTAGGATCACAGCCATGTGGATTAGTGGATAGTGACGAATTTGGATTACCAGTCCCTCCTTGTATTTGTG :  360
             TAGTAgCATTAATATTCAATGTAGGATCACAGCCATGTGgATTAGTGGATAGTGACGAATTTGGATTACCAGTCCCTCCTTGTATTTGTG

                       *       380         *       400         *       420         *       440         *
Guan_DV1   : CTTGGGTTAGTCACTGATCAGCTTTCATAATATCTGCTTATTAAAGATATGCTTATACGCAATTTCTCAATTTGGTATCACTCACGATAT :  450
Zheng_DV1  : CTTGGGTTAGTCACTGATCAGCTTTGATAATATCTACTTATTAAAGATATGCTTATATGCAATTTCTCAATTTGGTATCACTCACGATAT :  450
Liu_DV1    : CTTGGGTTAGTCACTGATCAGCTTTCATAATATCTACTTATTAAAGATATGCTTATATGCAATTTCTCAATTTGGTATCACTCACGATAT :  450
             CTTGGGTTAGTCACTGATCAGCTTTcATAATATCTaCTTATTAAAGATATGCTTATAtGCAATTTCTCAATTTGGTATCACTCACGATAT

                  460         *       480         *       500         *       520         *       540
Guan_DV1   : TTATGTGAATTGTGAAGCTGATAAATAGTTTAGAAAATACTATGAAGCCATGCCATTATTTATGAAGGATAAGGAGTGTGCACAAGACGA :  540
Zheng_DV1  : TTATGTAAATTGTGAAGCTGATAAATAGTTTAGAAAATACTATGAAGCCATGCCATTATTTATGAAGGATAAGGAGTGTGCACAAGACGA :  540
Liu_DV1    : TTATGTGAATTGTGAAGCTGATAAATAGTTTAGAAAATACTATGAAGCCATGCCATTATTTATGAAGGATAAGGAGTGTGCACAAGACGA :  540
             TTATGTgAATTGTGAAGCTGATAAATAGTTTAGAAAATACTATGAAGCCATGCCATTATTTATGAAGGATAAGGAGTGTGCACAAGACGA

                       *       560         *       580         *       600         *       620         *
Guan_DV1   : ATATTGTCAAATTGCTAAAGGAGACGATATCAACTATTGTTTAAAAAGTAGGTTCCGAGTAAATTACGAAATAAAGAATAATCTCATCAT :  630
Zheng_DV1  : ATATTGTCAAATTGCTAAAGGAGACGATATCAACTATTGTTTAAAAAGTAAGTTCCGAGTAAATTACGAAATAAAGAATAATCTCATCAT :  630
Liu_DV1    : ATATTGTCAAATTGCTAAAGGAGACGATATCAACTATTGTTTAAAAAGTAGGTTCCGAGTAAATTACGAAATAAAGAATAATCTCATCAT :  630
             ATATTGTCAAATTGCTAAAGGAGACGATATCAACTATTGTTTAAAAAGTAgGTTCCGAGTAAATTACGAAATAAAGAATAATCTCATCAT

                  640         *       660         *       680         *       700         *       720
Guan_DV1   : TTAATGTTGCTCTTTTAAAATCAGGAAATTGCGCTGGTGATAAGACTGATGCTCCAATGTGCATTTGTGGAGTAAGTAAAATTGTATCCA :  720
Zheng_DV1  : TTAATGTTGCTCTTTTAAAATCAGGAAATTGCGCTGGTGATAAGACTGATGCTCCAATGTGCATTTGTGGAGTAAGTAAAATTTTATCCA :  720
Liu_DV1    : TTAATGTTGCTCTTTTAAAATCAGGAAATTGCGCTGGTGATAAGACTGATGCTCCAATGTGCATTTGTGGAGTAAGTAAAATTTTATCCA :  720
             TTAATGTTGCTCTTTTAAAATCAGGAAATTGCGCTGGTGATAAGACTGATGCTCCAATGTGCATTTGTGGAGTAAGTAAAATTtTATCCA

                       *       740         *       760         *       780         *       800         *
Guan_DV1   : ATATCTATTTTTGTTATTATTATTATTATTATTAT-----------------------------TGTTATAAATGAACTATCTGTTGTAT :  781
Zheng_DV1  : ATATCTATTTTTGTTATTATTATTATTATTATTATATTATTATTATTATTATTATTATTATTATTGTTATAAATGAACTATCTGTTGTAT :  810
Liu_DV1    : ATATCTATTTTTGTTATTATTATTATTATT-----------------------------------GTTATAAATGAACTATCTGTTGTAT :  775
             ATATCTATTTTTGTTATTATTATTATTATTattat                             tGTTATAAATGAACTATCTGTTGTAT

                  820         *       840         *       860         *       880         *       900
Guan_DV1   : TTCAGGATAAGCGATGCAGCCATAAGGAAGACTGCAAGAAAGTTGGGGACAAATTACAATGTGTTGAAAGTGAGTTTCTCTTAGTTGCAT :  871
Zheng_DV1  : TTCAGGATAAGCGATGCAGCCATAAGGAAGACTGCAAGAAAGTTGGGGACAAATTACAATGTGTTGAAAGTGAGTTTCTCTTAGTTGCAT :  900
Liu_DV1    : TTCAGGATAAGCGATGCAGCCATAAGGAAGACTGCAAGAAAGTTGGGGACAAATTACAATGTGTTGAAAGTGAGTTTCTCTTAGTTGCAT :  865
             TTCAGGATAAGCGATGCAGCCATAAGGAAGACTGCAAGAAAGTTGGGGACAAATTACAATGTGTTGAAAGTGAGTTTCTCTTAGTTGCAT

                       *       920         *       940         *       960         *       980         *
Guan_DV1   : TTGTTGCTACCATGATTTTAAAACATTATATGATTGTATGCACACAAGGATCACTCGATAATTGCATTGAGCTTTGTAATTACTCCTGTA :  961
Zheng_DV1  : TTGTTGCTACCATGATTTTAAAACATTATATGATTGTATGTACACAAGGATCACTCGATAATTGCATTGAGCTTTGTAATTACTCCTGTA :  990
Liu_DV1    : TTGTTGCTACCATGATTTTAAAACATTATATGATTGTATGCACACAAGGATCACTCGATAATTGCATTGAGCTTTGTAATTACTCCTGTA :  955
             TTGTTGCTACCATGATTTTAAAACATTATATGATTGTATGcACACAAGGATCACTCGATAATTGCATTGAGCTTTGTAATTACTCCTGTA

                 1000         *      1020         *      1040         *      1060         *      1080
Guan_DV1   : TCTTGATCAATCATCGGATGTGAGTGATCTCAACTCAAAGAAAGTTTAAACAAAGCTCAGACATGTCTCTTCACAATGATATAGGCTTT- : 1050
Zheng_DV1  : TCTTGATCAATCATCGGATGTGAGTGATCTCAACTCAAAGAAAGTTTAAACAAAGCTCAGACATGTCTCTTCACAATGATATAGGCTTT- : 1079
Liu_DV1    : CCTTGATCAATCATCGGATGTGAGTGATCTCAACTCAAAGAAAGTTTAAACAAAGCTCAGACATGTCTCTTCACAATGATATAGGCTTTA : 1045
             tCTTGATCAATCATCGGATGTGAGTGATCTCAACTCAAAGAAAGTTTAAACAAAGCTCAGACATGTCTCTTCACAATGATATAGGCTTT

                       *      1100         *      1120         *      1140         *      1160         *
Guan_DV1   : --ATATTTTGCCATAGGATAACAATTTTGTTTAATGAGTGATTTGTAGAAATTCTTATAGATGATTTAAGATGCATTAGGAGCACGTTTT : 1138
Zheng_DV1  : --ATATTTTGCCATAGGATAACAATTTTGTTTAATGAGTGATTTGTAGAAATTCTTATAGATGATTTAAGATGCATTAGGAGCACGTTTT : 1167
Liu_DV1    : CAATATTTTGCCATAGGATAACAATTTTGTTTAATGAGTGATTTGTAGAAATTCTTATAGATGATTTAAGATGCATTAGGAGCACGTTTT : 1135
               ATATTTTGCCATAGGATAACAATTTTGTTTAATGAGTGATTTGTAGAAATTCTTATAGATGATTTAAGATGCATTAGGAGCACGTTTT
```

```
                     1180         *      1200         *      1220         *      1240         *      1260
Guan_DV1   : TCAGAAAAGTGCTAAACAAAGCCACAAAGATTTTAAGATATAACATCAATAATAAAATATTGCAGCAGGTTTCGGTTCCGTTTCTCAGCA : 1228
Zheng_DV1  : TCAGAAAAGTGCTAAACAAAGCCACAAAGATTTTAAGATATAACATCAATAATAAAATATTGCAGCAGGTTTCGGTTCCGTTTCTCAGCA : 1257
Liu_DV1    : TCAGAAAAGTGCTAAACAAAGCCACAAAGATTTTAAGATATAACATCAATAATAAAATATTGCAGCAGGTTTCGGTTCCCTTTCTCAGCA : 1225
             TCAGAAAAGTGCTAAACAAAGCCACAAAGATTTTAAGATATAACATCAATAATAAAATATTGCAGCAGGTTTCGGTTCCgTTTCTCAGCA

                        *      1280         *      1300         *      1320         *      1340         *
Guan_DV1   : AATGCATGTTTAAGCTGCAGCCCCTTTGATTTCATTAAAATTTTGCTACTAACTAATTTTTAACCAAAATTAAATAAAGGTCTATTTGAAA : 1318
Zheng_DV1  : AATGCATGTTTAAGCTGCAGCCCCTTTGATTTCATTAAAATTTTGCTACTAACTAATTTTTAACCAAAATTAAATAAAAGTCTATTTGAAA : 1347
Liu_DV1    : AATGCTTGTTTAAGCTGCAGCCCCTTTGATTTCATTAAAATTTTGCTACTAACTAATTTTTAACCAAAATTAAATAAAGGTCTATTTGAAA : 1315
             AATGCaTGTTTAAGCTGCAGCCCCTTTGATTTCATTAAAATTTTGCTACTAACTAATTTTTAACCAAAATTAAATAAAgGTCTATTTGAAA

                     1360         *      1380         *      1400         *      1420         *      1440
Guan_DV1   : GCTTCATGATTTATGAAAGATCCAGAAAGCTATGGGATATGTTAACTCAGCATTTATAAATGTTACTTACTAAATGTAGCTGAATTCTTT : 1408
Zheng_DV1  : GCTTCAAGATTTATGAAAGATCCATTCAGCTATGGAATATGTTAACTCAGCATTTATAAATGTTACTTACTAAATGTAGCTGAATTCTTT : 1437
Liu_DV1    : GCTTCATGATTTATGAAAGATCCAGAAAGCTATGGGATATGTTAACTCAGCATTTATAAATGTTACTTACTAAATGTAGCTGAATTCTTT : 1405
             GCTTCAtGATTTATGAAAGATCCAgaaAGCTATGGgATATGTTAACTCAGCATTTATAAATGTTACTTACTAAATGTAGCTGAATTCTTT

                        *      1460         *      1480         *      1500         *      1520         *
Guan_DV1   : TCCATTATATGTTGTGTTAATTGGCTGTTGCTTTTTATCACGTTTTTTTTTT--AATTTTAAAAATAATTTTTTAATAAATTCTTTACGA : 1496
Zheng_DV1  : TCCATTATATGTTGTGTTAATTGGCTGTTGCTTTTTATCACGTTTTTTTTTTTTAATTTTAAAAATAATTTTTTAATAAATTCTTTACGA : 1527
Liu_DV1    : TCCATTATATGTTGTGTTAATTGGCTGTTGCTTTTTATCACGTTTTTTTTT---AATTTTAAAAATAATTTTTTAATAAATTCTTTACGA : 1492
             TCCATTATATGTTGTGTTAATTGGCTGTTGCTTTTTATCACGTTTTTTTTTt  AATTTTAAAAATAATTTTTTAATAAATTCTTTACGA

                     1540         *      1560         *      1580         *      1600         *      1620
Guan_DV1   : ACTGGTTTACGAATGCTTTGGATGTCACAGGAATACAGTGTTGGTTTAATGCAGAGGTAACATGAGGTAGTTGACTAAAGTTTTAGTAGT : 1586
Zheng_DV1  : ACTGGTTTACGAATTCTTTGGATGTCACAGGAATACAGTGTTGGTTTAATGCAGAGGTAACATGAGGTAGTTGACTAAAGTTTTAGTA-- : 1615
Liu_DV1    : ACTGGTTTACGAATTCTTCGGATGTCACAGGAATACAGTGTTGGTTTAATGCAGAGGTAACATGAGGTAGTTGACTAAAGTTTTAGTA-- : 1580
             ACTGGTTTACGAATtCTTtGGATGTCACAGGAATACAGTGTTGGTTTAATGCAGAGGTAACATGAGGTAGTTGACTAAAGTTTTAGTA

                        *      1640         *      1660         *      1680         *      1700         *
Guan_DV1   : ATATGATTTTATTTATCCTGAATGCTAAAATATATCTAAATATATATAAAGATACACTTGTTGTTTATGGACGAAAAGAAGAAATTTCAA : 1676
Zheng_DV1  : -TATGATTTTATTTATCCTGAGTGCTAAAATATATCTAGATATATATAAAGATACACTTGTTGTTTATGGACGAAAAGAAGAAATTTCAA : 1704
Liu_DV1    : -TATGATTTTATTTATCCTGAGTGCTAAAATATATCTAGATATATATAAAGATACACTTGTTGTTTATGGACGAAAAGAAGAAATTTCAA : 1669
              TATGATTTTATTTATCCTGAgTGCTAAAATATATCTAgATATATATAAAGATACACTTGTTGTTTATGGACGAAAAGAAGAAATTTCAA

                     1720         *      1740         *      1760         *      1780         *      1800
Guan_DV1   : CGAATTATACATGAATGTACAAAAGTGACATTAACGTTGATATTAATATTAAATACAGAACCGAAGCTTTGTTCAAAAGTGAAGAAAGAT : 1766
Zheng_DV1  : CGAATTATACATGAATGTACAAAAGTGACATTAACGTTGATATTAATATTAAATACAGAACCGAAGCTTTGTTCAAAAGTGAAGAAAGAT : 1794
Liu_DV1    : CGAATTATACATGAATGTACAAAAGTGACATTAACGTTGATATTAATATTAAATACAGAACCGAAGCTTTGTTCAAAAGTGAAGAAAGAT : 1759
             CGAATTATACATGAATGTACAAAAGTGACATTAACGTTGATATTAATATTAAATACAGAACCGAAGCTTTGTTCAAAAGTGAAGAAAGAT

                        *      1820         *      1840         *      1860         *      1880         *
Guan_DV1   : GAATTTGGAATACCGATTCGTCCCTGTGCTTGTCAATGGGTTAGTATTGATCAATTACTGGTAATTTATCTACTCACTAAAAAACGAGCT : 1856
Zheng_DV1  : GAATTTGGAATACCGATTCGTCCCTGTGCTTGTCAATGGGTTAGTATCGATCAATTACTGGTAATTTATCTACTCACTAAAAAACGAGCT : 1884
Liu_DV1    : GAATTTGGAATACCGATTCGTCCCTGTGCTTGTCAATGGGTTAGTATCGATCAATTACTGGTAATTTATCTACTCACTAAAAAACGAGCT : 1849
             GAATTTGGAATACCGATTCGTCCCTGTGCTTGTCAATGGGTTAGTATcGATCAATTACTGGTAATTTATCTACTCACTAAAAAACGAGCT

                     1900         *      1920         *      1940         *      1960         *      1980
Guan_DV1   : TGTATGTAATTTCTCAATTACTCATGACATTTATGTCACTGTAAAGTTTTAATTAATTAAAATGAGGCGCTTCATTCTTTATACGCTCAT : 1946
Zheng_DV1  : TGTATGTAATTTCTCAATTACTCATGACATTTATGTCACTGTAAAGTTTTAATTAATTAAAATGAGGCGCTTCATTCTTTATACGCTCAT : 1974
Liu_DV1    : TGTATGTAATTTCTCAATTACTCATGACATTTATGTCACTGTAAAGTTTTAATTAATTAAAATGAGGCGCTTCATTCTTTATACGCTCAT : 1939
             TGTATGTAATTTCTCAATTACTCATGACATTTATGTCACTGTAAAGTTTTAATTAATTAAAATGAGGCGCTTCATTCTTTATACGCTCAT

                        *      2000         *      2020         *      2040         *      2060         *
Guan_DV1   : CCATCCATTCATTCATCCACGCATTCGTCCATCTTATCCTTCCTTCCATCTATAAATAAATCCTACCACCCATCCCCATCCAACTTAACC : 2036
Zheng_DV1  : CCATCCATTCATTCATCCACGCATTCGTCCATCTTATCCTTCCTTCCATCTATAAATAAATCCTACCACCCATCCCCCAGCGACTCAACC : 2064
Liu_DV1    : CCATCCATTCATTCATCCACGCATTCGTCCATCTTATCCTTCCTTCCATCTATAAATAAATCCTACCACCCATCCCCATCCAACTCAACC : 2029
             CCATCCATTCATTCATCCACGCATTCGTCCATCTTATCCTTCCTTCCATCTATAAATAAATCCTACCACCCATCCCCatcCaACTcAACC

                     2080         *      2100         *      2120         *      2140         *      2160
Guan_DV1   : AATTTACA-CTATGATTCAGAAAACATAGTGGTGATTTCGTACCATTATTTATCAAGGGTGTTGAGTGTGAAGAGCACGAGTATTGTAAT : 2125
Zheng_DV1  : AATTTAAAACAATGATTTAGAAAACATAGTGGTGAATTCGTACCATTATTTATCAAGGGTGTAGAGTGTGAAGAGCACGAGTATTGTAAT : 2154
Liu_DV1    : AATTTACA-CTATGATTCAGAAAACATGGTGGTGATTTCGTACCATTATTTATCAAGGGTGTTGAGTGTGAAGAGCACGAGTATTGTAAT : 2118
             AATTTACA CtATGATTcAGAAAACATaGTGGTGAtTTCGTACCATTATTTATCAAGGGTGTtGAGTGTGAAGAGCACGAGTATTGTAAT

                        *      2180         *      2200         *      2220         *      2240         *
Guan_DV1   : ATTACTGAAGGATACTCCATCGGCACTTGTGAACGATGTAAGTTCAGAGAAAATTGCGAAATAAACAATAATATCATAATAGG------- : 2208
Zheng_DV1  : ATTACTAAAGGATACTCCATCGGCACTTGTGAACAATGTAAGTTCAGAGAAAATTTAGAAATAAAAAATAATATCATAATTGGTTAATTT : 2244
Liu_DV1    : ATTACTAAAGGATACTCCATCGGCACTTGTGAACGATGTAAGTTTCGAGAAAATTTAGAAATAAAAAATAATATCATAATTGGTTAATTT : 2208
             ATTACTaAAGGATACTCCATCGGCACTTGTGAACgATGTAAGTTcaGAGAAAATTtaGAAATAAAaAATAATATCATAATtGGttaattt

                     2260         *      2280         *      2300         *      2320         *      2340
Guan_DV1   : -ATGATTAATTTTGACATTTATTTTCTATTAATATTTAACATAATAAG-------------GTGAATATTTGAATTAATAGATTTATATC : 2284
Zheng_DV1  : GATGATTAATTTTGACATTTAGTTTCTATTAATATTTAATATAATAAG-------------GTGAATATTTAAATTATTAGAATTATATC : 2321
Liu_DV1    : GATGATTAATTTTGACATTTAGTTTCTATTAATATTTAATATAATAAGTATAATAAATATAATAAATATTTGAATTAATAGAATTATATC : 2298
             gATGATTAATTTTGACATTTAgTTTCTATTAATATTTAAtATAATAAG             gTgAATATTTgAATTAaTAGAaTTATATC

                        *      2360         *      2380         *      2400         *      2420         *
Guan_DV1   : CTAAAAAAATCGAGAAATAAGTTAAATTTAAATGTCAGATTAAACTGGTTAACATTCAAACAAAAACGTTTTAAAATCAGTGTATTGTGC : 2374
Zheng_DV1  : CTAAAAATA--GAGAAATAAGTTAAATTTAAATATCAGATTAAACTGGTTAACATTAAAACATAAACGTTTTAAAATCAGTGTATTGTGC : 2409
Liu_DV1    : CTAAAAATA--GAGAACTAAGTTAAATTTAAATGTCAGATTAAACTGGTTAACATTAAAATAAAAACGTTTCAAAATCAGTGTATTGTGC : 2386
             CTAAAAAtA  GAGAAaTAAGTTAAATTTAAATgTCAGATTAAACTGGTTAACATTaAAAcAaAAACGTTTtAAAATCAGTGTATTGTGC
```

```
                  2440         *      2460         *      2480         *      2500         *      2520
Guan_DV1  : GGGTGAACCGGCTGTAGATTCTCCAGTGTGCATATGTAAAGTCAGTAACATTTTATCTAATGCTAGTAATTATCAATTATTAATAATAAT : 2464
Zheng_DV1 : GGGTAACCCGGCTGAAGATTCTCCAGTGTGCTTATGTAAAGTCAGTAACATTTTATCTAATGCTAATAATTATCAATTATTAATAATATT : 2499
Liu_DV1   : GGGTAACCCGGCTGAAGATTCTCCAGTGTGCTTATGTAAAGTCAGTAACATTTTATCTAATGCTAATAATTATCAATTATTAATAATATT : 2476
            GGGTaAcCCGGCTGaAGATTCTCCAGTGTGCtTATGTAAAGTCAGTAACATTTTATCTAATGCTAaTAATTATCAATTATTAATAATAtT

                     *      2540         *      2560         *      2580         *      2600         *
Guan_DV1  : AAGGATAATTATGAGGAGAAAATGTAGGAATGTCATTTGAATTTCAGGATAAGCGATGCGACCGTAATCAATACTGTAGGAACGTTGATG : 2554
Zheng_DV1 : AAAGATAATTATGAGGAGAAAATGTAGGAATGTCATTTGTCTTTCAGGATAAGCGATGCGACCGTAATCAATACTGTAGGAACGTTGATG : 2589
Liu_DV1   : AAAGATAATTATGAGGAGAAAATGTAGGAATGTCATTTGTCTTTCAGGATAAGCGATGCGACCGTAATCAATACTGTAGGAACGTTGATG : 2566
            AAaGATAATTATGAGGAGAAAATGTAGGAATGTCATTTGtcTTTCAGGATAAGCGATGCGACCGTAATCAATACTGTAGGAACGTTGATG

                  2620         *      2640         *      2660         *      2680         *      2700
Guan_DV1  : GCAAGTTGGAATGTGATGAAAGTGAGTTTCTCTTACCTGTTTTTTTT-ATTGTGAGACAAATTATTT-AAAGCTGTTAATATCATGATTT : 2642
Zheng_DV1 : GCAAGTTGGAATGTGATGAAAGTGAGTTTCTCTTACCTGTTTTTTTTTATTGTGAGACAAGTTATTTGAAAGTTGCTAATATCAGGATTT : 2679
Liu_DV1   : GCAAGTTGGAATGTGATGAAAGTGAGTTTCTCTTACCTGTTTTTTTTTATTGTGAGACAAGTTATTTGAAAGTTGCTAATATCAGGATTT : 2656
            GCAAGTTGGAATGTGATGAAAGTGAGTTTCTCTTACCTGTTTTTTTTtATTGTGAGACAAgTTATTTgAAAGtTGcTAATATCAgGATTT

                     *      2720         *      2740         *      2760         *      2780         *
Guan_DV1  : AAAAATAT-TCTGTGTGTAATATTGAAGTAATAATAATAATAATTTAAATATATAATATGTGCATGTACCACTATTACTACT-----TAG : 2726
Zheng_DV1 : AAAAAAAAATCTACATGTAATATTG---------TAATAATAATATAAATATATATTATGTATATGTATTATTACTACTACTACTTCTAT : 2760
Liu_DV1   : AAAAAAAAATCTACATGTAATATTG---------TAATAATAATATAAATATATATTATGTGTATGTATTATTACTACTACTACTTCTAT : 2737
            AAAAAaAaaTCTacaTGTAATATTG         TAATAATAATaTAAATATATAtTATGTgtATGTAttAtTAcTACTACTacttcTAt

                  2800         *      2820         *      2840         *      2860         *      2880
Guan_DV1  : CAATAATAATAATAATAATAATGTATTT-----CATCAAACTGAGCCTTAACAGAAGGTTATGACTAATGCTCTAATATTTTATGTAAGA : 2811
Zheng_DV1 : TAGTGGTAATAATAATAATAATGTATTTGAAATCATCAAACGGAGTCTTAAGAGAAGGTAATAACTAATGCTCTAATATTTTATGTAAAA : 2850
Liu_DV1   : TAGTGGTAATAATAATAATAATGTATTTGAAATCATCAAACGGAGTCTTAAGAGAAGGTAATAACTAATGCTCTAATATTTTATGTAAAA : 2827
            tAgTggTAATAATAATAATAATGTATTTgaaatCATCAAACgGAGtCTTAAgAGAAGGTaATaACTAATGCTCTAATATTTTATGTAAaA

                     *      2900         *      2920         *      2940         *      2960         *
Guan_DV1  : TTGATTGCACATTGCTCTTAGGTTACTGATTTAGTTAATTTGATTTATTATCAAATGAGGGACATTTTTTGCCAGCTCTGCCGATTATCA : 2901
Zheng_DV1 : TTAAGTGCGTATTGCTCTTTGGTTACTAATTTAATAA--------------CAAATGAGCTAATATTTTTGCCAGCTATGCCTATTATCA : 2926
Liu_DV1   : TTAAGTGCGTATTGCTCTTTGGTTACTAATTTAATAA--------------CAAATGAGCTAATATTTTTGCCAGCTATGCCTATTATCA : 2903
            TTaAgTGCgtATTGCTCTTtGGTTACTaATTTAaTaA              CAAATGAGctAataTTTTTGCCAGCTaTGCCtATTATCA

                  2980         *      3000         *      3020         *      3040         *      3060
Guan_DV1  : GTTAATCCGCTGTTAAGTATTATCACGTTATTATTAAATACAGAACCGAAGAGTTGCGAAATTATGGAGAAGGACAAACTAGGTCGACCT : 2991
Zheng_DV1 : GTTAATCCGCTGTTAAGTATTATTATGTTATTATTAAATACAGAACCGAAGAGTTGCGAAAATATGGAGAAGGACAAACTAGGTCGACCT : 3016
Liu_DV1   : GTTAATCCGCTGTTAAGTATTATTACGTTATTATTAAATACAGAACCGAAGAGTTGCGAAAATATGGAGAAGGACAAACTAGGTCGACCT : 2993
            GTTAATCCGCTGTTAAGTATTATtAcGTTATTATTAAATACAGAACCGAAGAGTTGCGAAAaTATGGAGAAGGACAAACTAGGTCGACCT

                     *      3080         *      3100         *      3120         *      3140         *
Guan_DV1  : CTTGGGCCTTGCGTTTGTGGAACTGTTAGTCGTCGATAAATTTTGGTTATCGGAATACTTTTTAAAATCGTTCTTAAATTTAATTTATGA : 3081
Zheng_DV1 : ATTGGGCCTTGCGTTTGTGGAACTGTTAGTCGTCGATAAATTTTGGTTATCGGAATACTTTTTAAAATCGTATTCAAATTCAATTTATCA : 3106
Liu_DV1   : ATTGGGCCTTGCGTTTGTGGAACTGTTAGTCGTCGATAAATTTTGGTTATCGGAATACTTTTTAAAATCGTATTCAAATTCAATTTATGA : 3083
            aTTGGGCCTTGCGTTTGTGGAACTGTTAGTCGTCGATAAATTTTGGTTATCGGAATACTTTTTAAAATCGTatTcAAATTcAATTTATgA

                  3160         *      3180         *      3200         *      3220         *      3240
Guan_DV1  : ATTTATTATACATATTTTAAACATTGTAAAGTTTAGAAGACATTTATGAAACCACAATCATTATTTACCCAGGATCGGGTGTGTGAAAGG : 3171
Zheng_DV1 : ATTTATTATAAAAATTTTAAACATTGTAAAGTTTGGAAGACATTTATGAAGTCACAATCATTATTTACCAAGGGTCGGGTGTGTGAAAGG : 3196
Liu_DV1   : ATTTATTATAAAAATTTTAAACATTGTAAAGTTTGGAAGACATTTATGAAGTCACAATCATTATTTACCAAGGGTCGGGTGTGTGAAAGG : 3173
            ATTTATTATAaAaATTTTAAACATTGTAAAGTTTgGAAGACATTTATGAAgtCACAATCATTATTTACCaAGGgTCGGGTGTGTGAAAGG

                     *      3260         *      3280         *      3300         *      3320         *
Guan_DV1  : CATGAATACTGTGATATTACACTTGAAGATAACACCAACAATTGCTTACCAAGTACGTTTCGTTTAGATTATGAAATAAACAATTATATC : 3261
Zheng_DV1 : CACGAATACTGTGATATTACACTTAAAGATGCCACCGACAATTGCTTACCAAGTACGTTTCGTTTAGATTATGAAATAAACAATTATACC : 3286
Liu_DV1   : CACGAATACTGTGATATTACACTTAAAGATGCCACCGACAATTGCTTACCAAGTACGTTTCGTTTAGATTATGAAATAAACAATTATACC : 3263
            CAcGAATACTGTGATATTACACTTaAAGATgcCACCgACAATTGCTTACCAAGTACGTTTCGTTTAGATTATGAAATAAACAATTATAcC

                  3340         *      3360         *      3380         *      3400         *      3420
Guan_DV1  : AAGATTTAGAGATTAATTTTCGCTATCGATTTTTATTAATTCTTTGATTGTGCCCAGAGTACAATCCGTGCGAACAAGGGTCAATTGTAG : 3351
Zheng_DV1 : AAGATTTAGGGATTAATATTCAC-ATTGATTTTTACTAATTCTTTGATTGTACACAGAGTACAATCCGTGCAAACAAGGGTCATTTGTAG : 3375
Liu_DV1   : AAGATTTAGGGATTAATTTTCAC-CTTGATTTTTACTAATTCTTTGATTGTACACAGAGTACAATCCGTGCAA-CAAGGGTCATTTGTAG : 3351
            AAGATTTAGgGATTAATtTTCaC aTtGATTTTTAcTAATTCTTTGATTGTaCaCAGAGTACAATCCGTGCaAaCAAGGGTCAtTTGTAG

                     *      3440         *      3460         *      3480         *      3500         *
Guan_DV1  : AGAAGAAATGTTTATGTGGTGTTAGTAACTATTACTGACAAACATTCTTCAATATTTCCAAC-AAAAGATAATTATTACATTAAAGTACC : 3440
Zheng_DV1 : AGAAGAAATGTTCATGTGGTGTTAGTAACTATTACTGACAAACATTCTTCAGTATTTCCAACTAAAAGATTATTATTACATTAAAATACC : 3465
Liu_DV1   : AGAAGAAATGTTCATGTGGTGTTAGTAACTATTACTGACAAACATTCTTCAGTATTTCCAACTAAAAGATTATTATTACATTAAAATACC : 3441
            AGAAGAAATGTTcATGTGGTGTTAGTAACTATTACTGACAAACATTCTTCAgTATTTCCAACtAAAAGATtATTATTACATTAAAaTACC

                  3520         *      3540         *      3560         *      3580         *      3600
Guan_DV1  : TAAAAATAACATTAAATAAAGTTTGTCATTTGTTACATATCTAGAAAAAGTCTTGCAGCAAAGGGAAGAAATGCATATTGATTGACGGCG : 3530
Zheng_DV1 : TAAATATAACATTAAATAAAGTTTGTCATTTGTTACATATCTAGAAAAAGTCTTGCAGCAAAGGGAAGAAATGCGTATTGATTGACGGCG : 3555
Liu_DV1   : TAAATATAACATTAAATAAAGTTTGTCATTTGTTACATATCTAGAAAAAGTCTTGCAGCAAAGGGAAGAAATGCGTATTGATTGACGGCG : 3531
            TAAAtATAACATTAAATAAAGTTTGTCATTTGTTACATATCTAGAAAAAGTCTTGCAGCAAAGGGAAGAAATGCgTATTGATTGACGGCG

                     *      3620
Guan_DV1  : AAGCAAATTGCACAAGGTTGTAA : 3553
Zheng_DV1 : AAGCAAATTGCATAAGGTTGTAA : 3578
Liu_DV1   : AAGCAAATTGCATAAGGTTGTAA : 3554
            AAGCAAATTGCAtAAGGTTGTAA
```

Figure S2B. Multiple sequence alignments of putative decorsin Hman_DV1 proteins derived from the genome data of *H. manillesis* provided by Guan et al. (2020), Zheng et al. (2023) and Liu et al. (2023), respectively. The cysteine residues are marked in bold and yellow and the KGD motif is marked in cyan and bold. The signal peptide is underlined.

```
                            *        20         *        40         *        60         *        80         *
Guan_DV1   : MLVLIICLLLSFSLDVDANRSQPCELVDSDEFGLPVPPCICAWDKECAQDEYCQIAKGDDINYCLKRNCAGDKTDAPMCICGDKRCSHKE :  90
Zheng_DV1  : MLVLIICLLLSFSLDVDANRSQPCGLVDSDEFGLPVPPCICAWDKECAQDEYCQIAKGDDINYCLKRNCAGDKTDAPMCICGDKRCSHKE :  90
Liu_DV1    : MLVLIICLLLSFSLDVDANRSQPCGLVDSDEFGLPVPPCICAWDKECAQDEYCQIAKGDDINYCLKRNCAGDKTDAPMCICGDKRCSHKE :  90
             MLVLIICLLLSFSLDVDANRSQPCgLVDSDEFGLPVPPCICAWDKECAQDEYCQIAKGDDINYCLKRNCAGDKTDAPMCICGDKRCSHKE

                    100         *       120         *       140         *       160         *       180
Guan_DV1   : DCKKVGDKLQCVEKPKLCSKVKKDEFGIPIRPCACQWGVECEEHEYCNITEGYSIGTCERLYCAGEPAVDSPVCICKDKRCDRNQYCRNV : 180
Zheng_DV1  : DCKKVGDKLQCVEKPKLCSKVKKDEFGIPIRPCACQWGVECEEHEYCNITKGYSIGTCEQLYCAGNPAEDSPVCLCKDKRCDRNQYCRNV : 180
Liu_DV1    : DCKKVGDKLQCVEKPKLCSKVKKDEFGIPIRPCACQWGVECEEHEYCNITKGYSIGTCERLYCAGNPAEDSPVCLCKDKRCDRNQYCRNV : 180
             DCKKVGDKLQCVEKPKLCSKVKKDEFGIPIRPCACQWGVECEEHEYCNITkGYSIGTCErLYCAGnPAeDSPVC6CKDKRCDRNQYCRNV

                            *       200         *       220         *       240         *       260         *
Guan_DV1   : DGKLECDEKPKSCEIMEKDKLGRPLGPCVCGTDRVCERHEYCDITLEDNTNNCLPKYNPCEQGSIVEKKCLCGKKSCSKGKKCILIDGEA : 270
Zheng_DV1  : DGKLECDEKPKSCENMEKDKLGRPIGPCVCGTGRVCERHEYCDITLKDATDNCLPKYNPCKQGSFVEKKCSCGKKSCSKGKKCVLIDGEA : 270
Liu_DV1    : DGKLECDEKPKSCENMEKDKLGRPIGPCVCGTGRVCERHEYCDITLKDATDNCLPKYNPCEQGSFVEKKCSCGKKSCSKGKKCVLIDGEA : 270
             DGKLECDEKPKSCEnMEKDKLGRP6GPCVCGTgRVCERHEYCDITLkDaT1NCLPKYNPCeQGSfVEKKCsCGKKSCSKGKKC6LIDGEA


Guan_DV1   : NCTRL-  : 275
Zheng_DV1  : NCIRL-  : 275
Liu_DV1    : NCIRL-  : 275
             NCiRL
```
