# Supplementary material for: Monomeric and Oligomeric Decorsins of the Asian Medicinal Leech Hirudinaria manillensis
Source: Int J Mol Sci. 2025 Nov 14;26(22):11017. doi: 10.3390/ijms262211017 (PMC12651989; doi:10.3390/ijms262211017)
Supplement: Supplementary file 1 [file ijms-26-11017-s001.zip › File S3.pdf]

Figure S3A. Multiple sequence alignments of putative decorsin Hman_DV2 genes derived from the genome data of *H. manillensis* provided by Guan et al. (2020), Zheng et al. (2023) and Liu et al. (2023), respectively. The exons are labeled in green and the introns are labeled in red. Start and stop codons are marked in bold, the cysteine codons are marked in bold and yellow and the RGD/KGD motif encoding codons are marked in cyan and bold.

```
                         *        20         *        40         *        60         *        80         *
Guan_DV2   : ATGTTAGTTCTGATAATTTGTCTTCTACTGTCCTTTTCATTGGAAATCGATGCAAATAGTTCAGATAGTGAGTTTACACGATTCATTTGA :   90
Zheng_DV2  : ATGTTAGTTTTGATAATTTGTCTTCTACTGTCCTTTTCTTTGGTCATCGATGCAAATAGTTCAAATAGTGAGTTTATACGATTAATTTGA :   90
Liu_DV2    : ATGTTAGTTCTGATAATTTGTCTTCTACTGTCCTTTTCTTTGGTCATCGATGCAAATAGTTCAGATAGTGAGTTTACACGATTCATTTGA :   90
             ATGTTAGTTcTGATAATTTGTCTTCTACTGTCCTTTTCtTTGGtcATCGATGCAAATAGTTCAgATAGTGAGTTTAcACGATTcATTTGA

                  100         *       120         *       140         *       160         *       180
Guan_DV2   : GAAGATATATCAAAACATATGTATTATTTTTTCGATTGATATTTTAAAAGATTTT----TTTA--CCTAAATTCTTGTAAATAAAGATTA :  174
Zheng_DV2  : GAATATATATCAAAACATATGTATTATTTTTTATATTACTTTTTATTTTAATTTTAAACTGTAATTTTTAATTCTTGTAAATAAAGATTA :  180
Liu_DV2    : GAAGATATATCAAAACATATGTATTATTTTTTCGATTGATATTTTAAAAGATTTT----TTTA--CCTAAATTCTTGTAAATAAAGATTA :  174
             GAAgATATATCAAAACATATGTATTATTTTTTcgATTgaTaTTTtaaaagATTTT    TtTA  ccTaAATTCTTGTAAATAAAGATTA

                         *       200         *       220         *       240         *       260         *
Guan_DV2   : AAAATAAATAGTTATATTACAAATAAAATGCTATCGATATATTCGTTATTGTTATATTATTAATCTGTAGTAACATTAATATTCAATTCA :  264
Zheng_DV2  : AAAATAAATAGTTATATTACAAATAAAATGCTATCGATATATTCGTTATTGTTATATTATTAATCTGTAGTAACATTAATATTCAATTCA :  270
Liu_DV2    : AAAATAAATAGTTATATTACAAATAAAATGCTATCGATATATTCGTTATTGTTATATTATTAATCTGTAGTAACATTAATATTCAATTCA :  264
             AAAATAAATAGTTATATTACAAATAAAATGCTATCGATATATTCGTTATTGTTATATTATTAATCTGTAGTAACATTAATATTCAATTCA

                  280         *       300         *       320         *       340         *       360
Guan_DV2   : GCACCAAAGGATTGCCACTCAGTGGCGAAAGACAAATTTGGATTACCAACCGCTCCTTGTATTTGTGCTTGGGTTAGTCACTGTTTTGAT :  354
Zheng_DV2  : GCACCGAAGGATTGCCAATTAGTGGCGAAAGACAAATTTGGATTACCAACCGCTCCTTGTATTTGTGCTTGGGTTAGTCAC-----TGAT :  355
Liu_DV2    : GCACCAAAGGATTGCCACTCAGTGGCGAAAGACAAATTTGGATTACCAACCGCTCCTTGTATTTGTGCTTGGGTTAGTCACTGTTTTGAT :  354
             GCACCaAAGGATTGCCAcTcAGTGGCGAAAGACAAATTTGGATTACCAACCGCTCCTTGTATTTGTGCTTGGGTTAGTCACtgtttTGAT

                         *       380         *       400         *       420         *       440         *
Guan_DV2   : CATTTTTGATAATATGTCTGTTCATTAAAAACATGCTTATATGCAATTTCTCAATTTGGTATCACTCACGACATTTATATAAATTGTGAA :  444
Zheng_DV2  : CAGTTTTGATAATA--TCTACTTATTAAAGATATGCTTATATGCATTTTCTCAATTTGGTATCACTCACGACATTTATATAAATTCTGAA :  443
Liu_DV2    : CATTTTTGATAATATGTCTGTTCATTAAAAACATGCTTATATGCAATTTCTCAATTTGGTATCACTCACGACATTTATATAAATTGTGAA :  444
             CAtTTTTGATAATAtgTCTgtTcATTAAAaAcATGCTTATATGCAaTTTCTCAATTTGGTATCACTCACGACATTTATATAAATTgTGAA

                  460         *       480         *       500         *       520         *       540
Guan_DV2   : GCTGATAAATAGTTTAGAAAATACTATGAGGCCATGCCATTATTTATCAAGGATAAGGAGTGTGCAGAACACGAATATTGTCAACTTGCT :  534
Zheng_DV2  : GCTGATAAATAGTTTAGAAAATACTATGAAGCCATGCCATTATTTATCAAGGATAAGGAGTGTGCAGAACACGAATATTGTCAACTTGCT :  533
Liu_DV2    : GCTGATAAATAGTTTAGAAAATACTATGAGGCCATGCCATTATTTATCAAGGATAAGGAGTGTGCAGAACACGAATATTGTCAACTTGCT :  534
             GCTGATAAATAGTTTAGAAAATACTATGAgGCCATGCCATTATTTATCAAGGATAAGGAGTGTGCAGAACACGAATATTGTCAACTTGCT

                         *       560         *       580         *       600         *       620         *
Guan_DV2   : AGAGGAGACAATATCAACCAATGCTTAGGAAGTAAGTTTTGAGTAAATTGCTGAATGATAATGAAGAATAAAGAATAATGTCATAATTTA :  624
Zheng_DV2  : AGAGGAGACAATATCAACCAATGCTTAGGAAGTAAGTTTTGAGTAAATTGC-------------GAAATGAAGAATA--GTCATCATTTA :  608
Liu_DV2    : AGAGGAGACAATATCAACCAATGCTTAGGAAGTAAGTTTTGAGTAAATTGCTGAATGATAATGAAGAATAAAGAATAATGTCATAATTTA :  624
             AGAGGAGACAATATCAACCAATGCTTAGGAAGTAAGTTTTGAGTAAATTGCtgaatgataatgaagAATaAAGAATAatGTCATaATTTA

                  640         *       660         *       680         *       700         *       720
Guan_DV2   : ATGTTGCCCTTTTAAAATCAGAATATTGCACTGGTGACAAGACTGATGCTCCAATGTGGATTTGTGGAGTAAGTGAAATTTTATAAAATA :  714
Zheng_DV2  : ATGTTGCCCTTTTAAAATCAGAATATTGCACTGGTGAAAAGACTGATGCTCCAATGTGCGTTTGTGGAGTAAGTGAAATTTTATAAAATA :  698
Liu_DV2    : ATGTTGCCCTTTTAAAATCAGAATATTGCACTGGTGAAAAGACTGATGCTCCAATGTGCGTTTGTGGAGTAAGTGAAATTTTATAAAATA :  714
             ATGTTGCCCTTTTAAAATCAGAATATTGCACTGGTGAaAAGACTGATGCTCCAATGTGCgTTTGTGGAGTAAGTGAAATTTTATAAAATA

                         *       740         *       760         *       780         *       800         *
Guan_DV2   : TCTATTATTATTATTAAATGTTATTGTTATTATTATGAGAAAATAATAACATATCTTTTGTGTTTCAGGATAAGCGATGCGGGTATAACGA :  804
Zheng_DV2  : TCTATTATTATTA---ATGTTATTATTATTATTATGAGAAAATAATGACATATCTTTT-TGTTTCAGGATAAGCGATGCGGGTATAACGA :  784
Liu_DV2    : TCTATTATTATTATTAAATGTTATTATTATTATTATGAGAAAATAATAACATATCTTTTGTGTTTCAGGATAAGCGATGCGGGTATAACGA :  804
             TCTATTATTATTAttaATGTTATTaTTATTATTATGAGAAAATAATaACATATCTTTTgTGTTTCAGGATAAGCGATGCGGGTATAACGA

                  820         *       840         *       860         *       880         *       900
Guan_DV2   : GGACTGTAAGAAAGTTGAGGATAAATTACAATGTGTCGAAAGTGAGTTTCTCTTAGCTGCTTTTGTTGTGCTTTGAAATTGCTGCAACCA :  894
Zheng_DV2  : GGACTGCAAGAAAGTTGAGGATAAATTACAATGTGTCGAAAGTGAGTATCTCTTAGCTGTTTTT-TTGTGCTTTGAAATTGCTGCAACCA :  873
Liu_DV2    : GGACTGTAAGAAAGTTGAGGATAAATTACAATGTGTCGAAAGTGAGTTTCTCTTAGCTGCTTTTGTTGTGCTTTGAAATTGCTGCAACCA :  894
             GGACTGtAAGAAAGTTGAGGATAAATTACAATGTGTCGAAAGTGAGTtTCTCTTAGCTGcTTTTgTTGTGCTTTGAAATTGCTGCAACCA

                         *       920         *       940         *       960         *       980         *
Guan_DV2   : CGATTTTAAAACAAAATATGATTGTATACAAGGATCATATTTCATAAACGAGTCCATCTCTTCTTTAAATCTTGTTCGTACAGGATAATA :  984
Zheng_DV2  : CGATTTTAAAACAAAATATGATTGTACAGAAGGATCATATTTCATACACGAGTCCATGCCTTTTTAAAATCTTGTTCGTACGTGTTAATA :  963
Liu_DV2    : CGATTTTAAAACAAAATATGATTGTATACAAGGATCATATTTCATACACGAGTCCATGCCTTTTTAAAATCTTGGTCGTACAGGATAATA :  984
             CGATTTTAAAACAAAATATGATTGTAtAcAAGGATCATATTTCATAcACGAGTCCATgcCTTtTTaAAATCTTGtTCGTACagGaTAATA

               1000         *      1020         *      1040         *      1060         *      1080
Guan_DV2   : GCGACTCGTAATTTTTTCTGTTCTTTTTCGACTTTGGGAAAATTAATTTAGAATTAATATTAATTCAT-CGATAATTGCTTTGAGTTCTG : 1073
Zheng_DV2  : GCGACTCGTGATTTTTTCTGTT-------GATTTTGTGAAAATTAATTTAGAATTAATGTTAATTCACTCGATAATTACATTGAGTTTTG : 1046
Liu_DV2    : GCGACTCGTGATTTTTTCTGTT-------GACTTTGTGAAAATTAATATAGACTGAAAATTAATTCATTCGATAATTGCTTTGAGTTCTG : 1067
             GCGACTCGTgATTTTTTCTGTT       GAcTTTGtGAAAATTAATtTAGAaTtAAtaTTAATTCAttCGATAATTgCtTTGAGTTcTG

                         *      1100         *      1120         *      1140         *      1160         *
Guan_DV2   : CATTTACTCATGTACCTTGTTCAATCATCGGACGTGAGTGATCTCAACTCAAGGACTTTATGCAAAGTTTAGATATGTCCCTTCACAATG : 1163
Zheng_DV2  : TATTTACTCATGTACGT-GATCAATCATCGGACGTGAGTGATCTCAACTCAAA-AGTTTATACAAAGTTTAGATATATCTCTTCACAATG : 1134
Liu_DV2    : CATTTACTCATGTACCTTGTTCAATCATCGGACGTGAGTGATCTCAACTCAAGGACTTTATGCAAAGTTTAGATATGTCCCTTCACAATG : 1157
             cATTTACTCATGTACcTtGtTCAATCATCGGACGTGAGTGATCTCAACTCAAggAcTTTATgCAAAGTTTAGATATgTCcCTTCACAATG
```

```
                   1180         *      1200         *      1220         *      1240         *      1260
Guan_DV2  : ATAGGCTTGACAATAATTTGGCACAGGATAACATTTTTTTTAAATGAGTGATTTGAAGATATCCTCAAAGTTGAGTTGAGATGCTTTAGG : 1253
Zheng_DV2 : ATAGGCTTTACAATAATTTGCCACAGGATAACATTTTTTTTAAATGAGTGATTTGAATATATCCTCATAGTTGAATTAAGATGCGTTAGG : 1224
Liu_DV2   : ATAGGCTTGACAATAATTTGGCACAGGATAACATTTTTTTTAAATGAGTGATTTGAAGATATCCTCAAAGTTGAGTTGAGATGCTTTAGG : 1247
            ATAGGCTTgACAATAATTTGgCACAGGATAACATTTTTTTTAAATGAGTGATTTGAAgATATCCTCAaAGTTGAgTTgAGATGCtTTAGG

                *      1280         *      1300         *      1320         *      1340         *
Guan_DV2  : AGCATGTCTTTCAGAAAGGGTCTAAACACTTCACAATGACTGT-TTTTTTTTTATTATTTTTTTAAATTTAAAAAATGATTTTTTAATAA : 1342
Zheng_DV2 : AGCATGTCTTTCAGAAACGTTCTAAACACTTCACAATGACTGTGTTTTTTTTTATTATTTTTTTAAATTTAAAAAATGATTTTTTAATAA : 1314
Liu_DV2   : AGCATGTCTTTCAGAAAGGGTCTAAACACTTCACAATGACTGT-TTTTTTTTTATTATTTTTTTAAATTTAAAAAATGATTTTTTAATAA : 1336
            AGCATGTCTTTCAGAAAgGgTCTAAACACTTCACAATGACTGT TTTTTTTTTATTATTTTTTTAAATTTAAAAAATGATTTTTTAATAA

                   1360         *      1380         *      1400         *      1420         *      1440
Guan_DV2  : ATTCTTCACGAACTGGAGTACGAATTCTTTGGATATCGCAGGAAGACAGCATTGATTTATTTCAGAAGTAACATGAGGAAGTTGACTAAA : 1432
Zheng_DV2 : ATTTTTCACGAACTGGAGTACGAATTCTTTGGATATCGCAGGAAGACAGCATTGATTTATTTCAGAAGTAACATGAGGAAGTTGACTAAA : 1404
Liu_DV2   : ATTCTTCACGAACTGGAGTACGAATTCTTTGGATATCGCAGGAAGACAGCATTGATTTATTTCAGAAGTAACATGAGGAAGTTGACTAAA : 1426
            ATTcTTCACGAACTGGAGTACGAATTCTTTGGATATCGCAGGAAGACAGCATTGATTTATTTCAGAAGTAACATGAGGAAGTTGACTAAA

                *      1460         *      1480         *      1500         *      1520         *
Guan_DV2  : GATTTAGTAGCATAGTAGTTTCTTTATCCTGAATGCTGAAAGATATATATCCGG----ATATAA-GATAAGCTAGTTCGTTATAATCGAA : 1517
Zheng_DV2 : GATTTAGTAGCATAGTAGTTTCTTTATCCTAAATGCTGAAAGATATATATCCGG----ATATAAAGATAAGCTAGTTCGTTATAATCGAA : 1490
Liu_DV2   : GATTTAGTAGCATAGTAGTTTCTTTATCCTGAATGCTGAAAGATATATATCCGGTGATATATAA-GATAAGCTAGTTCGTTATAATCGAA : 1515
            GATTTAGTAGCATAGTAGTTTCTTTATCCTgAATGCTGAAAGATATATATCCGG    ATATAA GATAAGCTAGTTCGTTATAATCGAA

                   1540         *      1560         *      1580         *      1600         *      1620
Guan_DV2  : AAGATGAAA----------ATATATACATACATTTGGTAAAGGTGAGGTCACTTAACATTAATATTAATTGCAGAGCTGAAACAATGTTC : 1597
Zheng_DV2 : AAGATGAAATTTCAACGAAATATATACATACATTTGGTAAAGGTGAGGTCACTTAACATTAATATTAATTGCAGAGCTGAAGCAATGTTC : 1580
Liu_DV2   : AAGATGAAATTTCAACGAAATATATACATACATTTGGTAAAGGTGAGGTCACTTAACATTAATATTAATTGCAGAGCTGAAACAATGTTC : 1605
            AAGATGAAAtttcaacgaaATATATACATACATTTGGTAAAGGTGAGGTCACTTAACATTAATATTAATTGCAGAGCTGAAaCAATGTTC

                *      1640         *      1660         *      1680         *      1700         *
Guan_DV2  : AAGTGTGGAAAAAGATGAATATGGAATTCCAATTGGTCCTTGTAGTTGTGGCTGGGTTAGTCTTGATCAATTATTGATAATAACACATCC : 1687
Zheng_DV2 : AAGTGTGGAAAAAGATGAATATGGAATTCCAATTGGTCCTTGTAGTTGTGGTTGGGTTAGTCTTGATCAATTATTGATAATAACACATCC : 1670
Liu_DV2   : AAGTGTGGAAAAAGATGAATATGGAATTCCAATTGGTCCTTGTAGTTGTGGCTGGGTTAGTCTTGATCAATTATTGATAATAACACATCC : 1695
            AAGTGTGGAAAAAGATGAATATGGAATTCCAATTGGTCCTTGTAGTTGTGGcTGGGTTAGTCTTGATCAATTATTGATAATAACACATCC

                   1720         *      1740         *      1760         *      1780         *      1800
Guan_DV2  : ACCTACACACTCAACCAATTGACACTATGGTTTAGAAAACTTTATGCTGAAATCGTATCATTTTTTTACCAAGGATTTTATGGAGTGTGA : 1777
Zheng_DV2 : ACCTACACACTCAACCAATTGATACTATGGTTTAGAAAACTTTGTGCTGAAATAGTACCATTTTTA-ACCAAGGATTTTCTGGAGTGTGA : 1759
Liu_DV2   : ACCTACACACTCAACCAATTGACACTATGGTTTAGAAAACTTTATGCTGAAATCGTATCATTTTTTTACCAAGGATTTTCTGGAGTGTGA : 1785
            ACCTACACACTCAACCAATTGAcACTATGGTTTAGAAAACTTTaTGCTGAAATcGTAtCATTTTTttACCAAGGATTTTcTGGAGTGTGA

                *      1820         *      1840         *      1860         *      1880         *
Guan_DV2  : AAAGAACGAATATTGTGATATTAGTGATGGATTCTTAGGCTTTTGTGAGCGATGTAAGTTCCGAGTATATTGCGAAATAATAAT---GTT : 1864
Zheng_DV2 : AAAGAACGAATATTGTGATATTAGTGATGGATTCCTAGGCTTTTGTGAGCGATGTAAGTTCCGAGTATATTGCGAAATAATAATAATGTT : 1849
Liu_DV2   : AAAGAACGAATATTGTGATATTAGTGATGGATTCTTAGGCTTTTGTGAGCGATGTAAGTTCCGAGTATATTGCGAAATAATAATAATGTT : 1875
            AAAGAACGAATATTGTGATATTAGTGATGGATTCtTAGGCTTTTGTGAGCGATGTAAGTTCCGAGTATATTGCGAAATAATAATaatGTT

                   1900         *      1920         *      1940         *      1960         *      1980
Guan_DV2  : ATCAATTGGTGATGAAGTTTGACATTTTTTTCTCACATAAACGTTTAAAAATCAGTGTATTGTTCCGGTGACCCGGATAAAGATTCTCCA : 1954
Zheng_DV2 : ATCAATTGGTGATGAAGTTTGACATTTTTTTCTCACATAAACGTTTAAAAATCAGTGTATTGTTCCGGTGACCCGGATAAAGATTCTCCA : 1939
Liu_DV2   : ATCAATTGGTGATGAAGTTTGACATTTTTTTCTCACATAAACGTTTAAAAATCAGTGTATTGTTCCGGTGACCCGGATAAAGATTCTCCA : 1965
            ATCAATTGGTGATGAAGTTTGACATTTTTTTCTCACATAAACGTTTAAAAATCAGTGTATTGTTCCGGTGACCCGGATAAAGATTCTCCA

                *      2000         *      2020         *      2040         *      2060         *
Guan_DV2  : GTGTGCATATGTGAAGTCAGTAACATCTTATCTAATGCTAATAATTATTATTATAAATTAATAATAATAATAATAAAGATAATTATGACG : 2044
Zheng_DV2 : GTGTGCATATGTGAAGTCAGTAACATCTTATCTAATGCTAATAATTATTATTATAAATTAATAATAATAATAATAAAGATAATTATGACG : 2029
Liu_DV2   : GTGTGCATATGTGAAGTCAGTAACATCTTATCTAATGCTAATAATTATTATTATAAATTAATAATAATAATAATAAAGATAATTATGACG : 2055
            GTGTGCATATGTGAAGTCAGTAACATCTTATCTAATGCTAATAATTATTATTATAAATTAATAATAATAATAATAAAGATAATTATGACG

                   2080         *      2100         *      2120         *      2140         *      2160
Guan_DV2  : AGAAAATGAAGAAATGTCTTTTGTCTTTCAGGATAAGCGATGCAACCATGATCAATACTGTAGGAACGTTGATGGCAAGTTGGTATGTCA : 2134
Zheng_DV2 : AGAAAATGAAGAAATGTCTTTTGTCTTTCAGGATAAGCGATGCAACCATGATCAATACTGTGCGAACGTTGATGGCAAGTTGGTATGTCA : 2119
Liu_DV2   : AGAAAATGAAGAAATGTCTTTTGTCTTTCAGGATAAGCGATGCAACCATGATCAATACTGTGCGAACGTTGATGGCAAGTTGGTATGTCA : 2145
            AGAAAATGAAGAAATGTCTTTTGTCTTTCAGGATAAGCGATGCAACCATGATCAATACTGTgcGAACGTTGATGGCAAGTTGGTATGTCA

                *      2180         *      2200         *      2220         *      2240         *
Guan_DV2  : TGAATGTGAGTTTGTCTTAGCTGTTTT----TTTTATTGTGAGACACGTTATTTTAAAGTTGTTAATATCACGATTT-AAAAAAAATATTA : 2219
Zheng_DV2 : TGAATGTGAGTTTGTCTTAATTGTTTTGTTTTTTTATTGTGAGACACGTTATTTTAAAGTTGTTAATATCACGATTTTAAAAAAAATATTA : 2209
Liu_DV2   : TGAATGTGAGTTTGTCTTAGTTGTTTT----TTTTATTGTGAGACACGTTATTTTAAAGTTGTTAATATCACGATTT-AAAAAAAATATTA : 2230
            TGAATGTGAGTTTGTCTTAgtTGTTTT    TTTTATTGTGAGACACGTTATTTTAAAGTTGTTAATATCACGATTT AAAAAAAATATTA

                   2260         *      2280         *      2300         *      2320         *      2340
Guan_DV2  : TTATAGTATTACATATAATAATTTATTTATATAATATTATTATTAACAACGTTATTATTATTAATATTAGAGAATTTCTTGATATTAATA : 2309
Zheng_DV2 : TTATAGTATTACATATAATAATTTATTTATATAATATTATTATTAACAACGTTATTATTATTAATATTAGAGAATTTCTTGATATTAATA : 2299
Liu_DV2   : TTATAGTATTACATATAATAATTTATTTATATAATATTATTATTAACAACGTTATTATTATTAATATTAGAGAATTTCTTGATATTAATA : 2320
            TTATAGTATTACATATAATAATTTATTTATATAATATTATTATTAACAACGTTATTATTATTAATATTAGAGAATTTCTTGATATTAATA

                *      2360         *      2380         *      2400         *      2420         *
Guan_DV2  : TTGATATTATTTTATTATTAATATAAATATTATTTTTAATAAGAAGACGATATAAGCATATAATTGGAATATCATTTTTATTAATAAAAT : 2399
Zheng_DV2 : TTGATATTATTTTATTATTAATATAAATATTATTTTTAATAAGAAGACGATA-AAGCATATAAT-AGAATATCATTTTTATTATTAAA-T : 2386
Liu_DV2   : TTGATATTATTTTATTATTAATATAAATATTATTTTTAATAAGAAGACGATATAAGCATATAATAGGAATATCATTTTTATTAATAAAAT : 2410
            TTGATATTATTTTATTATTAATATAAATATTATTTTTAATAAGAAGACGATAtAAGCATATAAT gGAATATCATTTTTATTAaTAAAaT
```

```
                    2440         *        2460         *        2480         *        2500         *        2520
Guan_DV2   : ACTATAAGAATTCCTAAATCACAATATATTAGCAATGTTAATATTTTCTTTTAGCTTTTTTGTTTTTATCTCCAGGACTCTGAATGTAGT : 2489
Zheng_DV2  : ACTATAAGAATTCCTAAATCACAATATATTAGCAATGTTAATATTTTCTTTTAGCTTTTTTGTTTTTATCTCCAGAACTCTGAATGTAGT : 2476
Liu_DV2    : ACTATAAGAATTCCTAAATCACAATATATTAGCAATGTTAATATTTTCTTTTAGCTTTTTTGTTTTTATCTCCAGGACTCTGAATGTAGT : 2500
             ACTATAAGAATTCCTAAATCACAATATATTAGCAATGTTAATATTTTCTTTTAGCTTTTTTGTTTTTATCTCCAGgACTCTGAATGTAGT

                      *        2540         *        2560         *
Guan_DV2   : ACCAAAGACAGATGCATTGTCACAAAAGGAGATTATAGTAACTACTGCGATTGA : 2543
Zheng_DV2  : ACCAAAGACAGATGCATTGTCACAAAAGGAGATTATAGTAACTACTGCGATTGA : 2530
Liu_DV2    : ACCAAAGACAGATGCATTGTCACAAAAGGAGATTATAGTAACTACTGCGATTGA : 2554
             ACCAAAGACAGATGCATTGTCACAAAAGGAGATTATAGTAACTACTGCGATTGA
```

Figure S3B. Multiple sequence alignments of putative decorsin Hman_DV2 proteins derived from the genome data of *H. manillesis* provided by Guan et al. (2020), Zheng et al. (2023) and Liu et al. (2023), respectively. The cysteine residues are marked in bold and yellow and the RGD/KGD motifs are marked in cyan and bold. The signal peptide is underlined.

```
                      *        20         *        40         *        60         *        80         *
Guan_DV2   : MLVLIICLLLSFSLEIDANTPKDCHSVAKDKFGLPTAPCICAWDKECAEHEYCQLARGDNINQCLGKYCTGDKTDAPMCICGDKRCGYNE :  90
Zheng_DV2  : MLVLIICLLLSFSLVIDANTPKDCQLVAKDKFGLPTAPCICAWDKECAEHEYCQLARGDNINQCLGKYCTGEKTDAPMCVCGDKRCGYNE :  90
Liu_DV2    : MLVLIICLLLSFSLVIDANTPKDCHSVAKDKFGLPTAPCICAWDKECAEHEYCQLARGDNINQCLGKYCTGEKTDAPMCVCGDKRCGYNE :  90
             MLVLIICLLLSFSLvIDANTPKDChsVAKDKFGLPTAPCICAWDKECAEHEYCQLARGDNINQCLGKYCTGeKTDAPMC6CGDKRCGYNE

                    100         *        120         *        140         *        160         *        180
Guan_DV2   : DCKKVEDKLQCVEKLKQCSSVEKDEYGIPIGPCSCGWDFMECEKNEYCDISDGFLGFCERLYCSGDPDKDSPVCICEDSECSTKDRCIVT : 180
Zheng_DV2  : DCKKVEDKLQCVEKLKQCSSVEKDEYGIPIGPCSCGWDFLECEKNEYCDISDGFLGFCERLYCSGDPDKDSPVCICENSECSTKDRCIVT : 180
Liu_DV2    : DCKKVEDKLQCVEKLKQCSSVEKDEYGIPIGPCSCGWDFLECEKNEYCDISDGFLGFCERLYCSGDPDKDSPVCICEDSECSTKDRCIVT : 180
             DCKKVEDKLQCVEKLKQCSSVEKDEYGIPIGPCSCGWDF6ECEKNEYCDISDGFLGFCERLYCSGDPDKDSPVCICE1SECSTKDRCIVT

                      *
Guan_DV2   : KGDYSNYCD-  : 189
Zheng_DV2  : KGDYSNYCD-  : 189
Liu_DV2    : KGDYSNYCD-  : 189
             KGDYSNYCD
```
