# Supplementary material for: Monomeric and Oligomeric Decorsins of the Asian Medicinal Leech Hirudinaria manillensis
Source: Int J Mol Sci. 2025 Nov 14;26(22):11017. doi: 10.3390/ijms262211017 (PMC12651989; doi:10.3390/ijms262211017)
Supplement: Supplementary file 1 [file ijms-26-11017-s001.zip › File S4.pdf]

Figure S4A. Multiple sequence alignments of putative decorsin Hman_DV3 genes derived from the genome data of *H. manillensis* provided by Guan et al. (2020), Zheng et al. (2023) and Liu et al. (2023), respectively. The exons are labeled in green and the introns are labeled in red. Start and stop codons are marked in bold, the cysteine codons are marked in bold and yellow and the RGD motif encoding codons are marked in cyan and bold.

```
                   *        20         *        40         *        60         *        80         *
Guan_DV3  : ATGATAGTGTTGATGATTTGTCTTGCCCTGCCTGCGGGTTTTGCAAATGGTATGGTATATTATTTTCAATCGCATGTTAATGCTGATTAA :   90
Zheng_DV3 : ATGATAGTGTTGATGATTTGTCTTGCCCTGCCTGTGGGTTTTGCAAATGGTATGGTATATTATTTTCAATCGCATGTTAATGCTGATTAA :   90
Liu_DV3   : ATGATAGTGTTGATGATTTGTCTTGCCCTGCCTGTGGGTTTTGCAAATGGTATGGTATATTATTTTCAATCGCATGTTAATGCTGATTAA :   90
            ATGATAGTGTTGATGATTTGTCTTGCCCTGCCTGtGGGTTTTGCAAATGGTATGGTATATTATTTTCAATCGCATGTTAATGCTGATTAA

                  100         *       120         *       140         *       160         *       180
Guan_DV3  : TGCCGTTATTTGATAATCAATTATCGTGTTTTTGTTTCCTATTAACCTTTTAAATGCCAAGAAATTAGAATTTACGGATGATTACGGCCA :  180
Zheng_DV3 : TGCCGTTATTTGATAATTAATTATCGTGTTTTTGTTTCCTATTAACCTTTTAAATGCCAAGAAATTAGAATTTACGGATGATTACGGCCA :  180
Liu_DV3   : TGCCGTTATTTGATAATCAATTATCGTGTTTTTGTTTCCTATTAACCTTTTAAATGCCAAGAAATTATAATTTACGGATGATTACGGCCA :  180
            TGCCGTTATTTGATAATcAATTATCGTGTTTTTGTTTCCTATTAACCTTTTAAATGCCAAGAAATTAgAATTTACGGATGATTACGGCCA

                   *       200         *       220         *       240         *       260         *
Guan_DV3  : CTACAATCATCCTTTAATCTTCAATATTGATCCTTTAGGGTATTTTTTGAAATAATAAGTTTTGTTATTAATCCAGATTAACAATAACTA :  270
Zheng_DV3 : CTATAATCATCCTTTAATCTTCAATATTGATCCTTTAGGGTATTTTTTGAAATAATAAGTTTTGTTATTAATCCAGATTAACAATAACTA :  270
Liu_DV3   : CTACAATCATCCTTTAATCTTCAATATTGATCCTTTAGGGTATTTTTTGAAATAATAAGTTTTGTTATTAATCCAGATTAACAATAACTA :  270
            CTAcAATCATCCTTTAATCTTCAATATTGATCCTTTAGGGTATTTTTTGAAATAATAAGTTTTGTTATTAATCCAGATTAACAATAACTA

                  280         *       300         *       320         *       340         *       360
Guan_DV3  : ATTGTAAAGATGGTAAGCTTAAATCATTTTTCAATCATCAGTATAATTATCTATGTGTCATATGAATGCTATAAATGACATTTTATGTTA :  360
Zheng_DV3 : ATTG---------TAAGCTTAAATCATTTTTCAATCATCAGTATAATTATCTATGTGTCATATGAATGCTATAAATGACATTTTATGTTA :  351
Liu_DV3   : ATTGTAAAGATGGTAAGCTTAAATCATTTTTAAATCATCAGTATAATTATCTATGTGTCATATGAATGCTATAAATGACATTTTATGTTA :  360
            ATTGtaaagatggTAAGCTTAAATCATTTTTcAATCATCAGTATAATTATCTATGTGTCATATGAATGCTATAAATGACATTTTATGTTA

                   *       380         *       400         *       420         *       440         *
Guan_DV3  : TACATACATGTATGTACTTCATATAAATATAGAATAATAATCAATATTATTAGTATTATTAAATCGATGACTATGTTGTGTTTGTTTACA :  450
Zheng_DV3 : TACATACATGTATGTACTTCATATACATATAGAATAATAATCAATATTATTAGTATTATTAAATCGATGACTATGTTGTGTTTGTTTACA :  441
Liu_DV3   : TACATACATGTATGTACTTCATATACATATAGAATAATAATCAATATTATTAGTACTATTAAATCGATGACTATGTTGTGTTTGTTTACA :  450
            TACATACATGTATGTACTTCATATAcATATAGAATAATAATCAATATTATTAGTAtTATTAAATCGATGACTATGTTGTGTTTGTTTACA

                  460         *       480         *       500         *       520         *       540
Guan_DV3  : TGAAGCCGATTTACAGTCTTGTCGCTCGGTGAAATTCGATTGGAGGAATCAACCTATTACTGCTTGCAAATGCGGGGTAATAATAAACTT :  540
Zheng_DV3 : TGAAGCCGATTTACAGTCTTGTCGCTCGGTGAAATTCGATTGGAGGAATCAACCTATTACTGCTTGCAAATGCGGGGTAATAATAAACTT :  531
Liu_DV3   : TGAAGCCGATTTACAGTCTTGTCGCTCGGTGAAATTTGATTGGAGGAATCAACCTATTACTGCTTGCAAATGCGGGGTAATAATAAACTT :  540
            TGAAGCCGATTTACAGTCTTGTCGCTCGGTGAAATTcGATTGGAGGAATCAACCTATTACTGCTTGCAAATGCGGGGTAATAATAAACTT

                   *       560         *       580         *       600         *       620         *
Guan_DV3  : AAATAATAATTAAAATTCAAAATAATATTTGGTCTTTTTTTTTAATGACAAAAGCAACTTGATTTTTTTCC-GATGTTCACAATTAATTA :  629
Zheng_DV3 : AAATAATAATTAAAATTCAAAATAATATTTGGTCTTTTTTTTTAATGACAAAAGCAACTTGATTTTTTTCC-GATGTTCACAATTAATTA :  620
Liu_DV3   : AAATAATAATTAAAATTCAAAATAATATTTGGTCTTTTTTTTTAATGACAAA-GCAACTTGATTTTTTTCCCGATGTTCACAATTAAATA :  629
            AAATAATAATTAAAATTCAAAATAATATTTGGTCTTTTTTTTTAATGACAAAaGCAACTTGATTTTTTTCC GATGTTCACAATTAAtTA

                  640         *       660         *       680         *       700         *       720
Guan_DV3  : ATAACATAATTTATAATAAGTATAAGTATCTAATAATAATAAGTATAAGTTTATCTAAGATCATGTCATAGATTTATAATCCTCTCACAG :  719
Zheng_DV3 : ATAACATAATTTATAATAAGTATAAGTATCTAATAATAATAAGTATAAGTTTATCTAAGATCATGTCATAGATTTATAATCCTCTCACAG :  710
Liu_DV3   : ATAACATAATTTATAATAAGTATAAGTATCTAATAATAATAAGTATAAGTTTATCTAAGATCATGTCATTGATTTATAATCCTCTCACAG :  719
            ATAACATAATTTATAATAAGTATAAGTATCTAATAATAATAAGTATAAGTTTATCTAAGATCATGTCATaGATTTATAATCCTCTCACAG

                   *       740         *       760         *       780         *       800         *
Guan_DV3  : GAAAGGCAATGCCACGAGTACGAAGAGTGCGTGATGCCGAGAGGCGATTCCTTTGATTACTGCAGACAGGGTATTACTAATTCCTAATTC :  809
Zheng_DV3 : GAAAGGCAATGCCACGAGTACGAAGAGTGCGTGATGCCGAGAGGCGATTCCTTTGATTACTGCAGACAGGGTATTACTAATTCCTAATTC :  800
Liu_DV3   : GAAAGGCAATGCCACGAGTACGAAGAGTGCGTGATGCCGAGAGGCGATTCCTTTGATTACTGCAGACAGGGTATTACTAATTCCTAATTC :  809
            GAAAGGCAATGCCACGAGTACGAAGAGTGCGTGATGCCGAGAGGCGATTCCTTTGATTACTGCAGACAGGGTATTACTAATTCCTAATTC

                  820         *       840         *       860         *       880         *       900
Guan_DV3  : CTAATTTTATCTTTCTGCTTTTTTAATTATTTGGTGAAAGTAATTCATGTATTTGAAAAAAAA-TAACAACAAAGAATAATTTAAATGT :  898
Zheng_DV3 : CTAATTTTATCTTTCTGCTTTTTTAATTATTTGGTGAAAGTAATTCATGTATTTGAAAAAAAAATAACAACAAAGAATAATTTAAATGT :  890
Liu_DV3   : CTAATTTTATCTTTCTGCTTTTTTAATTATTTGGTGAAAGTAATTCATGTATTTGAAAAAAAA-TAACAACAAAGAATAATTTAAATGT :  898
            CTAATTTTATCTTTCTGCTTTTTTAATTATTTGGTGAAAGTAATTCATGTATTTGAAAAAAAA TAACAACAAAGAATAATTTAAATGT

                   *       920         *       940         *       960         *       980         *
Guan_DV3  : GAATATAACATTTTACTAACGAATATAGTCATTCGGATATGATAGAGATTAAATGATAATACTATTTTATTTTAAAACCAGTTCATCCTT :  988
Zheng_DV3 : GAATATAACATTTTACTAACGAATATAGTCATTCGGATATGATAGAGATTAAATGATAATACTATTTTATTTTAAAACCAGTTCATCCTT :  980
Liu_DV3   : GAATATAACATTTAACTGACGAATATAGTCATTCGGAGATGATAGAGATTAAATAATAATACTATTTTATTTCAAAAACAGTTCATCCTT :  988
            GAATATAACATTTtACTaACGAATATAGTCATTCGGAtATGATAGAGATTAAATgATAATACTATTTTATTTtAAAAcCAGTTCATCCTT

                 1000         *      1020         *      1040         *      1060         *      1080
Guan_DV3  : TTTGTACTGGCGATACGGTTAAGGACGACTGGGATTGCTTTTGTGGAGTAAGCCAAATTTAAGTACAATATATTTAAATATTACATATTT : 1078
Zheng_DV3 : TTTGTACTGGCGATACGGTTAAGGACGACTGGGATTGCTTTTGTGGAGTAAGCCAAATTTAAGTA-AATATATTTAAATATTACATATTT : 1069
Liu_DV3   : TTTGTACTGGCGATACGGTTAAGGACGACTGGGACTGCTTTTGCGGAGTAAGCCAAATTTAAGTAAAATAT--TCAAATATTACATATTT : 1076
            TTTGTACTGGCGATACGGTTAAGGACGACTGGGAtTGCTTTTGtGGAGTAAGCCAAATTTAAGTA AATATatTtAAATATTACATATTT

                   *      1100         *      1120         *      1140         *      1160         *
Guan_DV3  : AATAATTAAATAAATCTAATAGTTATGCCAGCAAACATGTACTCT--TCTTC-TTCTCATTATTACTATTAATATTATTATTATTATTAT : 1165
Zheng_DV3 : AATAATTAAATAAATCTAATAGTTATGCCAGCAAACATGTACTTTCTTCTTCATTATTACTATTATTATTAATATTATTATTATTATTAT : 1159
Liu_DV3   : AATAATTAAATAAATCTAATAGTTATGCCAGCAAACATGTACTTT--TCTTC-TTCTCATTATTACTATTAATATAAATATTATTATTAT : 1163
            AATAATTAAATAAATCTAATAGTTATGCCAGCAAACATGTACTtT  TCTTC TTcTcAtTATTAcTATTAATATtAtTATTATTATTAT
```

```
                 1180         *       1200         *       1220         *       1240         *       1260
Guan_DV3  : TATTATCATTATTATTACTATTATTATTATTACTATTGT--------------------------------TATTATTTTTATTATCGAT : 1223
Zheng_DV3 : TATTATTATTATTATTATTATTATTATTATTATTATTATCATTATTATTACTATTATTATTATTACTATTTTATTATTTTTATTATCGAT : 1249
Liu_DV3   : TATTATCATTATTATTACTATTATTATTATTACTATTGT--------------------------------TATTATTTTTATTATCGAT : 1221
            TATTATcATTATTATTAcTATTATTATTATTAcTATTgT                                TATTATTTTTATTATCGAT

                    *       1280         *       1300         *       1320         *       1340         *
Guan_DV3  : GTCATTAATTTTAAACAAAATGAAAAAATGGTATTTGTCTTTCAGGATAGCCGATGCCGTCACACGGAAGACTGCAAGAAAGTTGAAGGC : 1313
Zheng_DV3 : GTCATTAATTTTAAACAAAATGAAAAAATGGTATTTGTCTTTCAGGATAGCCGATGCCGTCACACGGAAGACTGCAAGAAAGTTGAAGGC : 1339
Liu_DV3   : GTCATTAATTTTAAACAAAATGAAAAAATGGTATTTGTCTTTCAGGATAGACGATGCCGTCACACGGAAGACTGCAAGAAAGTTGAAGGC : 1311
            GTCATTAATTTTAAACAAAATGAAAAAATGGTATTTGTCTTTCAGGATAGcCGATGCCGTCACACGGAAGACTGCAAGAAAGTTGAAGGC

                 1360         *       1380         *       1400         *       1420         *       1440
Guan_DV3  : GTCTTACAATGCGTCGAAAGTGAGTCTCTCTAAGTTGTTTCAATGTAAAATAAAAAAAATAATAAAATGAAAATAACAAAAAATTAAAT : 1403
Zheng_DV3 : GTCTTACAATGCGTCGAAAGTGAGTCTCTCTAAGTTGTTTCAATGTAAAATTAAAAAAAATAATAAAATGAAAATAACAAAAAATTAAAT : 1429
Liu_DV3   : GTCTTACAATGCGTCGAAAGTGAGTCTCTCTAAGTTGTTTCAATGTAAAATAAAAAAA--TTATAAAATAAAAATAACAAAAAATTAAAT : 1399
            GTCTTACAATGCGTCGAAAGTGAGTCTCTCTAAGTTGTTTCAATGTAAAATaAAAAAAAaTaATAAAATgAAAATAACAAAAAATTAAAT

                    *       1460         *       1480         *       1500         *       1520         *
Guan_DV3  : ATATTTATAATACACAAAATATGCATAATACATATAGGTAATACAATATAATATATGTATGATGCTATCCATATATTAGTTATTATTATA : 1493
Zheng_DV3 : ATATTTATAATACACAAAATATGCATAATACATATAGGTAATACAAAATAATATATATATGATGCTATCCATATATTAGTTATTATTATA : 1519
Liu_DV3   : ATATTTACAATACATAA--TATACATA-TACATATAGGTAATATAATATAATATATGTATGTTACTATCCATATATTAGTTATTATTATA : 1486
            ATATTTAtAATACAcAAaaTATgCATAaTACATATAGGTAATAcAAtATAATATATgTATGaTgCTATCCATATATTAGTTATTATTATA

                 1540         *       1560         *       1580         *       1600         *       1620
Guan_DV3  : TTATTAATCCGGTTAAGTAGTTAAGCGGTTAAGTAGTAACATAGATATTCAATGCAGGACCAGAGGATTGCACAGTCGTGGAAAGAGACG : 1583
Zheng_DV3 : TTATTAATCCGGTTAAGTAGTTAAGCGGTTAAGTAGTAACATAGATATTCAATGCAGGACCAGAGGATTGCACAGTCGTGGAAAGAGACG : 1609
Liu_DV3   : TTATTAATCCGGTTACGTAGTTAAGCGGTTAAGTAGTAAGATAGATATTCAATGCAGGACCAGAGGATTGCACAGTCGTGGAGAGAGACG : 1576
            TTATTAATCCGGTTAaGTAGTTAAGCGGTTAAGTAGTAAcATAGATATTCAATGCAGGACCAGAGGATTGCACAGTCGTGGAaAGAGACG

                    *       1640         *       1660         *       1680         *       1700         *
Guan_DV3  : AATTTGGATTACCAATCGGTCCTTGCATTTGTGCCTGGGTTAGTCACTGATCAGTTTTAATAATATGTATGCTCAGTAAAACGTGCTCAT : 1673
Zheng_DV3 : AATTTGGATTACCAATCGGTCCTTGCATTTGTGCCTGGGTTAGTCACTGATCAGTTTTAATAATATGTATGCTCAGTAAAACGTGCTCAT : 1699
Liu_DV3   : AATTTGGATTACCAATAGGTCCTTGCATTTGTGCCTGGGTTAGTCACTGATCAGTTTTAATAATATGTATGCTCATTAAAACGTGCTCAT : 1666
            AATTTGGATTACCAATcGGTCCTTGCATTTGTGCCTGGGTTAGTCACTGATCAGTTTTAATAATATGTATGCTCAgTAAAACGTGCTCAT

                 1720         *       1740         *       1760         *       1780         *       1800
Guan_DV3  : AAGCAATTTCTCAATTTGGTATCACTCACGACATTTATATAAATTATGAAGCTGATAAATAGTTTAAAAAATACTATGAGGCCATGCTAT : 1763
Zheng_DV3 : AAGCAATTTCTCAATTTGGTATCACTCACGACATTTATATAAATTATGAAGCTGATAAATAGTTTAAAAAATACTATGAGGCCATGCTAT : 1789
Liu_DV3   : AAGCAATTTCTCAATTTGGTATCACTCACGACATTTATATAAATTATGAAGCTGATAAATAGTTTAGAAAATACTACGAGGCCATGCTAT : 1756
            AAGCAATTTCTCAATTTGGTATCACTCACGACATTTATATAAATTATGAAGCTGATAAATAGTTTAaAAAATACTAtGAGGCCATGCTAT

                    *       1820         *       1840         *       1860         *       1880         *
Guan_DV3  : TATTTATCAAGGGTAAGGAGTGTGCAGATAACGAATATTGTCAAATTGCTAGAGGAGACAATATCAACCAATGCTTAGGAAGTAAGTTTT : 1853
Zheng_DV3 : TATTTATCAAGGGTAAGGAGTGTGCAGATAACGAATATTGTCAAATTGCTAGAGGAGACAATATCAACCAATGCTTAGGAAGTAAGTTTT : 1879
Liu_DV3   : TATTTATCAAGGGTAAGGAGTGTGCAGATAACGAATATTGTCAAATTGCTAGAGGAGACAATATCAACCAATGCTTAGGAAGTAAGTTTT : 1846
            TATTTATCAAGGGTAAGGAGTGTGCAGATAACGAATATTGTCAAATTGCTAGAGGAGACAATATCAACCAATGCTTAGGAAGTAAGTTTT

                 1900         *       1920         *       1940         *       1960         *       1980
Guan_DV3  : GAGTAAATTGCGTAATGAAAAATAATGTCATCATTTAATGTTGCCCTTTTAAAATCAGAATATTGCACTGGTAGGGCTAATGATCCACCA : 1943
Zheng_DV3 : GAGTAAATTGCGTAATGAAGAATAATGTCATCATTTAATGTTGCCCTTTTAAAATCAGAATATTGCACTGGTAGGGCTAATGATCCACCA : 1969
Liu_DV3   : GAGAAAATTGCGTAATGAAGAATAATGTCATCATTTAATGTTGCCCTTTTAAAATCAGAATATTGCACTGGTAGGGCTAATGATCCACCA : 1936
            GAGtAAATTGCGTAATGAAgAATAATGTCATCATTTAATGTTGCCCTTTTAAAATCAGAATATTGCACTGGTAGGGCTAATGATCCACCA

                    *       2000         *       2020         *       2040         *       2060         *
Guan_DV3  : AGGTGCATTTGTGGAGTAAGTGAAATTTTATGTAATATCTATTATTATAATTATTATTAATATAATAATATATAATAATAATATAATAAT : 2033
Zheng_DV3 : AGGTGCATTTGTGGAGTAAGTGAAATTTTATGTA-TATCTATTATTATAATTATTATTAATATAATAATA-ATAATAATA--ATAATAAT : 2055
Liu_DV3   : AGGTGCATTTGTGGAGTAAGTGAAATTTTATGTAATATCTATTATTATAATTATTATTAATATAATAATA-ATAATAATATAATAATAAT : 2025
            AGGTGCATTTGTGGAGTAAGTGAAATTTTATGTAaTATCTATTATTATAATTATTATTAATATAATAATA ATAATAATA  ATAATAAT

                 2080         *       2100         *       2120         *       2140         *       2160
Guan_DV3  : AATAATATAATAAT--------------TATTATTATTA--TTATGAGAAAATAATGAAATATCTTT-TGTATTTCAGGGTAAGCAATGC : 2106
Zheng_DV3 : A-TAATATAATATAA-------------TATTAATATAAAATAATATAAATATCTTTTATTATTATTATGTATTTCAGGGTAAGCAATGC : 2131
Liu_DV3   : AATAATATAATAATATAATAATAATATTTATTATTATTA--TTATGAGAAAATAATGAAATATCTTT-TGTATTTCAGGGTAAGCAATGC : 2112
            AaTAATATAATAAata             TATTAtTATtA  TtATgagAAaATaaTgaAaTATctTT TGTATTTCAGGGTAAGCAATGC

                    *       2180         *       2200         *       2220         *       2240         *
Guan_DV3  : GACCATAATCAATACTGCAGGAACTTTAATGGCAAATTGCAATGTACTAAAAGTGAGTTTTTCTTAAGTGTTTTATATAGTGAGACACGT : 2196
Zheng_DV3 : GACCATAATCAATACTGCAGGAACTTTAATGGCAATTTGCAATGTACTAAAAGTGAGTTTCTCTTAAGTGTTTTTTATTGTGAGACACGT : 2221
Liu_DV3   : GACCATAATCAATACTGCAGGAACTTTAATGGCAAATTGCAATGTACTAAAAGTGAGTTTCTCTTAAGTGTTTTTTATTGTGAGACACGT : 2202
            GACCATAATCAATACTGCAGGAACTTTAATGGCAAaTTGCAATGTACTAAAAGTGAGTTTcTCTTAAGTGTTTTtTATtGTGAGACACGT

                 2260         *       2280         *       2300         *       2320         *       2340
Guan_DV3  : TATTTTAAAGTTGTTAGTACCACGATTTATAAACAAATTCTATGTATAATATTGTAATAATAATAATAATTATAATAGTAATTATTATTA : 2286
Zheng_DV3 : TATTTTAAAGTT-TTAGTACCA---TTTATAAACAAATTCTATGTATAATATT------------------------------------A : 2271
Liu_DV3   : TATTTTAAAGTTGTTAGTACCACGATTTATAAACAAATTCTATGTATAATATG------------------------------------A : 2256
            TATTTTAAAGTTgTTAGTACCAcgaTTTATAAACAAATTCTATGTATAATATt                                    A

                    *       2360         *       2380         *       2400         *       2420         *
Guan_DV3  : TACTATTACATTAGTAAAGTATTTCATCAAACGGACCTTCTGTTATGACGATAGCTCTAATATTTGTTATATAAAGTTATGTGCACATTG : 2376
Zheng_DV3 : TACTATTACATTAGTAAAATATTTCATCAAACGGACCTTCTGTTATGACGATAGCTCTAATATTTGTTATATAAAGTTATGTGCACATTG : 2361
Liu_DV3   : TACTATTACATTAGTAACGTATTTCATCAAACGGACCTTCTGTTATGACGATAGCTCTAATATTTGTTATATAAAGTTATGTGCACATTG : 2346
            TACTATTACATTAGTAAagTATTTCATCAAACGGACCTTCTGTTATGACGATAGCTCTAATATTTGTTATATAAAGTTATGTGCACATTG
```

```
                 2440        *       2460        *       2480        *       2500        *       2520
Guan_DV3  : TTCTTAGGTTATTAATTTAGTTAAATTTAATTTGTAACAACTAAGTTAA-TTTGGCTATTAAAGGTTAATCCGCTGTAAACTATTATTTT : 2465
Zheng_DV3 : TTCTTAGGTTATTAATTTAGTTAAATTTAATTTGTAACAACTAAGTTAAGTTTGGCTATTAAAGGTTAATCCGTTGTAAACTATTATTAT : 2451
Liu_DV3   : TTCTTAGGTTATTAATTTAGTTAAATTTAATTTGTAACAACTAAGTTAA-TTTGGCTATTAAAGGTTAATCCGCTGTAAACTATTATTAT : 2435
            TTCTTAGGTTATTAATTTAGTTAAATTTAATTTGTAACAACTAAGTTAA TTTGGCTATTAAAGGTTAATCCGcTGTAAACTATTATTaT

                    *       2540        *       2560        *       2580        *       2600        *
Guan_DV3  : ATTATTATTAAATGCAGTACCGAATCGTTGCGAAAAGATGGAGAAGGACAAACTCGGTCGCCCCATTAGTCCTTGCGTTTGTGGAATGGT : 2555
Zheng_DV3 : ATTATTATTAAATGCAGTACCGAATCGTTGCGAAAAGATGGATAAGGACAAACTCGGTCGCCCCATTAGTCCTTGCGTTTGTGGAATGGT : 2541
Liu_DV3   : ATTATTATTAAATGCAGTACCGAATCGTTGCGAAAAGATGGAGAAGGACAAACTCGGTCGCCCCATTAGTCCTTGCGTTTGTGGAATGGT : 2525
            ATTATTATTAAATGCAGTACCGAATCGTTGCGAAAAGATGGAgAAGGACAAACTCGGTCGCCCCATTAGTCCTTGCGTTTGTGGAATGGT

                 2620        *       2640        *       2660        *       2680        *       2700
Guan_DV3  : TAGTCGTGGATCAAATTTGGTCATCGGAATGCTCTTACAAATCGTAATAATATTTTATTTAATTAGGCGATTTGATATTTAACATTCAAA : 2645
Zheng_DV3 : TAGTCGTGGATCAAATTTGGTCATCGGAATGCTCTTACAAATCGTAATAATATTTTATTTAATTAGGCGATTTGATATTTAACATTCAAA : 2631
Liu_DV3   : TAGTCGTGGATCAAATTTGGTCATCGGAATGCTCTTACAAATCGTAATAATATTTTATTTAATTAGGCGATTTGATATTTAACATTCAAA : 2615
            TAGTCGTGGATCAAATTTGGTCATCGGAATGCTCTTACAAATCGTAATAATATTTTATTTAATTAGGCGATTTGATATTTAACATTCAAA

                    *       2720        *       2740        *       2760        *       2780        *
Guan_DV3  : ACGGTGTAAAATTTAGAA-TTAATTTAGAAGACCTTTATGAAATCGCAATCATTATTTACCAAGGGTCAGGTGTGTGAAAAGCACGAATA : 2734
Zheng_DV3 : ACGGTGTAAAATTTAGAAATTAATTTAGAAGACCTTTATGAAATCGCAATCATTATTTACTAAGGGTCAGGTTTGTGAAAAGCACGAATA : 2721
Liu_DV3   : ACGGTGTAAAATTTAGAA-TTAATTTAGAAGACCTTTATGAAATCGCAATCATTATTTACCAAGGGTCAGGTGTGTGAAAAGCACGAATA : 2704
            ACGGTGTAAAATTTAGAA TTAATTTAGAAGACCTTTATGAAATCGCAATCATTATTTACcAAGGGTCAGGTgTGTGAAAAGCACGAATA

                 2800        *       2820        *       2840        *       2860        *       2880
Guan_DV3  : CTGTGATATGATACATGAAGATGCCACCAACTTTTGCTTACCAAGTATGTTTCGTTTAGATTATGAAA-TAAACAATTATACGAAGATTT : 2823
Zheng_DV3 : CTGTAATATAACACATGAAGGTGCAACCAAAGTTTGCTTACCAAGTATGTTTCGTTTAGATTATGAAAATAAACAATTATACCAAGATTT : 2811
Liu_DV3   : CTGTGATATGATACATGAAGATGCCACCAACTTTTGCTTACCAAGTATGTTTCGTTTAGATTATGAAA-TAAACAATTATACGAAGATTT : 2793
            CTGTgATATgAtACATGAAGaTGCcACCAActTTTGCTTACCAAGTATGTTTCGTTTAGATTATGAAA TAAACAATTATACgAAGATTT

                    *       2900        *       2920        *       2940        *       2960        *
Guan_DV3  : AGCGATTAATTCTCACATTGATTTATATTAACGCTTTGATTGTCCCTAGAATATCTCCCGTGCAAACCGGGGTCAATTGTTCAGAAGAAA : 2913
Zheng_DV3 : AGCGATTAATTCTCACATTGATTTATATTA----TTTGATTGTCCTTAGAATATCTCCCGTGCAAACTAAAGTCAATTGTAGAGAAGAAA : 2897
Liu_DV3   : AGCGATTAATTCTCACATTGATTTATATTAACGCTTTGATTGTCCCTAGAATATCTCCCGTGCAAACCGGGGTCAATTGTTCAGAAGAAA : 2883
            AGCGATTAATTCTCACATTGATTTATATTAacgcTTTGATTGTCCcTAGAATATCTCCCGTGCAAACcgggGTCAATTGTtcAGAAGAAA

                 2980        *       3000        *       3020        *       3040        *       3060
Guan_DV3  : TGTTTATGTGGTGTTAGTAATTA------TTACTAAC--AAACATTCTTTAGTATTGCCGATTAAGAGATAATTATTACATTAAAGTACC : 2995
Zheng_DV3 : TGTTTATGTGGTGTTAGTAACTAATACTGTTATTAACTTAACTATTCTTTAGTATTTCCGATTAAGAGATTATTATTATATTAAAGTACC : 2987
Liu_DV3   : TGTTTATGTGGTGTTAGTAATTA------TTACTAAC--AAACATTCTTTAGTATTGCCGATTAAGAGATAATTATTACATTAAAGTACC : 2965
            TGTTTATGTGGTGTTAGTAAtTA      TTAcTAAC  AAacATTCTTTAGTATTgCCGATTAAGAGATaATTATTAcATTAAAGTACC

                    *       3080        *       3100        *       3120        *       3140        *
Guan_DV3  : TAAAAATAACATTAAAATAAAGTTATCCATATCTTATACATGTAGAATAAGTCGTGCAACAAAGGAAAGAGATGCAAATCGATTAAAGGC : 3085
Zheng_DV3 : TAAAAATAATATTAAAATAAAGTTAGCCATATGTTATACATGTAGAATAAGTCGTGCAACAAAGGAAAGAAATGCAAATTGGTTAAAGGC : 3077
Liu_DV3   : TAAAAATAACATTAAAATAAAGTTATCCATATCTTATACATGTAGAATAAGTCGTGCAACAAAGGAAAGAGATGCAAATCGATTAAAGGC : 3055
            TAAAAATAAcATTAAAATAAAGTTAtCCATATcTTATACATGTAGAATAAGTCGTGCAACAAAGGAAAGAgATGCAAATcGaTTAAAGGC

                 3160        *       3180        *       3200        *       3220        *       3240
Guan_DV3  : GAAGAAAAATGCTTGGAAGGTTGTATACACATCTTCGTTTCTACATTGATGTTGTTAAAACATGAATTTGAGTTTTGGAATGATTATTAC : 3175
Zheng_DV3 : GAAGAAAAATGCTTGGAAGGTTGTATGCACACCTTCGTTTCTACATTGACGTTTTTGAAATATGAATTTGAGTTTTGGAATTATTATTAC : 3167
Liu_DV3   : GAAGAAAAATGCTTGGAAGGTTGTATACACATCTTCGTTTCTACATTGATGTTGTTGAAACATGAATTTGAGTTTTGGAATGATTATTAC : 3145
            GAAGAAAAATGCTTGGAAGGTTGTATaCACAtCTTCGTTTCTACATTGAtGTTgTTgAAAcATGAATTTGAGTTTTGGAATgATTATTAC

                    *       3260        *       3280        *       3300        *       3320        *
Guan_DV3  : TATTAATAATGCTATTAATATTATTAGATTAATATTATTATTAATTCTATTATTTTCAGCTGGCAAAAAGAAGAAGGTATCCAAAAAATC : 3265
Zheng_DV3 : TATTAATTATGCTATTA--------------ATATTATTATTAATTCTATTATTTTCAGTTAGCAAAAAGAAGAAGATATCCAAAAAATC : 3243
Liu_DV3   : TATTAATAATGCTATTAATATTATTAGATTAATATTATTATTAATTCTATTATTTTCAGTTAGCAAAAAGAAGAAGATATCCAAAAAATC : 3235
            TATTAATaATGCTATTAatattattagattaATATTATTATTAATTCTATTATTTTCAGtTaGCAAAAAGAAGAAGaTATCCAAAAAATC

                 3340
Guan_DV3  : CAGCAAAAATGCCTAATAA : 3284
Zheng_DV3 : CAGCAAAAATTCCGAATAA : 3262
Liu_DV3   : CAGCAAAACTTCCGAATAA : 3254
            CAGCAAAAaTtCCgAATAA
```

Figure S4B. Multiple sequence alignments of putative decorsin Hman_DV3 proteins derived from the genome data of *H. manillesis* provided by Guan et al. (2020), Zheng et al. (2023) and Liu et al. (2023), respectively. The cysteine residues are marked in bold and yellow and the RGD motifs are marked in cyan and bold. The signal peptide is underlined.

```
                         *        20         *        40         *        60         *        80         *
Guan_DV3  : MIVLMICLALPAGFANADLQSCRSVKFDWRNQPITACKCGERQCHEYEECVMPRGDSFDYCRQVHPFCTGDTVKDDWDCFCGDSRCRHTE :  90
Zheng_DV3 : MIVLMICLALPVGFANADLQSCRSVKFDWRNQPITACKCGERQCHEYEECVMPRGDSFDYCRQVHPFCTGDTVKDDWDCFCGDSRCRHTE :  90
Liu_DV3   : MIVLMICLALPVGFANADLQSCRSVKFDWRNQPITACKCGERQCHEYEECVMPRGDSFDYCRQVHPFCTGDTVKDDWDCFCGDRRCRHTE :  90
            MIVLMICLALPvGFANADLQSCRSVKFDWRNQPITACKCGERQCHEYEECVMPRGDSFDYCRQVHPFCTGDTVKDDWDCFCGDsRCRHTE

                  100         *       120         *       140         *       160         *       180
Guan_DV3  : DCKKVEGVLQCVERPEDCTVVERDEFGLPIGPCICAWGKECADNEYCQIARGDNINQCLGKYCTGRANDPPRCICGGKQCDHNQYCRNFN : 180
Zheng_DV3 : DCKKVEGVLQCVERPEDCTVVERDEFGLPIGPCICAWGKECADNEYCQIARGDNINQCLGKYCTGRANDPPRCICGGKQCDHNQYCRNFN : 180
Liu_DV3   : DCKKVEGVLQCVERPEDCTVVERDEFGLPIGPCICAWGKECADNEYCQIARGDNINQCLGKYCTGRANDPPRCICGGKQCDHNQYCRNFN : 180
            DCKKVEGVLQCVERPEDCTVVERDEFGLPIGPCICAWGKECADNEYCQIARGDNINQCLGKYCTGRANDPPRCICGGKQCDHNQYCRNFN

                         *       200         *       220         *       240         *       260         *
Guan_DV3  : GKLQCTKIPNRCEKMEKDKLGRPISPCVCGMGQVCEKHEYCDMIHEDATNFCLPKYLPCKPGSIVQKKCLCGNKSCNKGKRCKSIKGEEK : 270
Zheng_DV3 : GNLQCTKIPNRCEKMDKDKLGRPISPCVCGMGQVCEKHEYCNITHEGATKVCLPKYLPCKLKSIVEKKCLCGNKSCNKGKKCKLVKGEEK : 270
Liu_DV3   : GKLQCTKIPNRCEKMEKDKLGRPISPCVCGMGQVCEKHEYCDMIHEDATNFCLPKYLPCKPGSIVQKKCLCGNKSCNKGKRCKSIKGEEK : 270
            GkLQCTKIPNRCEKMeKDKLGRPISPCVCGMGQVCEKHEYC16iHEdATnfCLPKYLPCKpgSIV2KKCLCGNKSCNKGK4CKs6KGEEK

                  280         *
Guan_DV3  : CLEAGKKKKVSKKSSKNA-- : 288
Zheng_DV3 : CLEVSKKKKISKKSSKNSE- : 289
Liu_DV3   : CLEVSKKKKISKKSSKTSE- : 289
            CLEvsKKKK6SKKSSKnse
```
