# Supplementary material for: Monomeric and Oligomeric Decorsins of the Asian Medicinal Leech Hirudinaria manillensis
Source: Int J Mol Sci. 2025 Nov 14;26(22):11017. doi: 10.3390/ijms262211017 (PMC12651989; doi:10.3390/ijms262211017)
Supplement: Supplementary file 1 [file ijms-26-11017-s001.zip › File S5.pdf]

Figure S5A. Multiple sequence alignments of putative decorsin Hman_DV4 genes derived from the genome data of *H. manillensis* provided by Guan et al. (2020), Zheng et al. (2023) and Liu et al. (2023), respectively. The exons are labeled in green and the introns are labeled in red. Start and stop codons are marked in bold, the cysteine codons are marked in bold and yellow and the RGD motif encoding codons are marked in cyan and bold.

```
                     *        20         *        40         *        60         *        80         *
Guan_DV4  : ATGTTAGTTCTGATAATTTGTCTTCTACTGTCCTTTTCATTGGACATCAATGCAAAGAGTGAGAAACGATTCATTTGAGATGATATATCA :  90
Zheng_DV4 : ATGTTAGTTCTGATAATTTGTCTTCTACTCTCCTTTTCATTGGACATCAATGCAAAGAGTGAGAAACGATTCATTTGAGATGATATATCA :  90
Liu_DV4   : ATGTTAGTTCTGATAATTTGTCTTCTACTGTCCTTTTCATTGGACATCAATGCAAAGAGTGAGAAACGATTCATTTGAGATGATATATCA :  90
            ATGTTAGTTCTGATAATTTGTCTTCTACTgTCCTTTTCATTGGACATCAATGCAAAGAGTGAGAAACGATTCATTTGAGATGATATATCA

                   100         *       120         *       140         *       160         *       180
Guan_DV4  : AAACATATGCATTATTTATTATATTAATATTTTAGGCGAATTTTCATTAACTAAATTCTTGCATAACAGATAAATTTAAGTAAATAAATG : 180
Zheng_DV4 : AAACATATGCATTATTTATTATATTAATATTTTAGGCGAATTTTCATTAACTAAATTCTTGCATAACAGATAAATTTAAGTAAATAAATG : 180
Liu_DV4   : AAACATATGCATTATTTATTATATTAATATTTTAGGCGAAT------TAACTAAATTCTTGCATAACAGATAAATTTAAGTAAATAAATG : 174
            AAACATATGCATTATTTATTATATTAATATTTTAGGCGAATtttcatTAACTAAATTCTTGCATAACAGATAAATTTAAGTAAATAAATG

                     *       200         *       220         *       240         *       260         *
Guan_DV4  : TAATTAAAAAA-GATTAAAGATAAATTCTTATTTTAGAAATGAAGTAAAATAC---------ATCATGTCTAA---------CCATATA- : 250
Zheng_DV4 : TAATTAAAAAAAGATTAAAGATAAATTCTTATTTTAGAAATGAAGTAAAATATTATTTATGAATCATGTCTAAAAATAATGTCTATATAT : 270
Liu_DV4   : TAATTAAAAAA-GATTAAAGATAAATTCTTATTTTAGAAATGAAGTAAAATAC---------ATCATGTCTAA---------CCATATA- : 244
            TAATTAAAAAA GATTAAAGATAAATTCTTATTTTAGAAATGAAGTAAAATAc         ATCATGTCTAA         CcATATA

                   280         *       300         *       320         *       340         *       360
Guan_DV4  : -CAATTAACCAATCTTCAAAATACGTTGATTAGGAACTGGTTATTAATAATTACGCATATTTAATTAGCAATAAATATTAATTGAAATTA : 339
Zheng_DV4 : TCAATTAACCAATCTTCAAAATACGTTGATTAGGAACTGGTTATTAATAATTACGCATATTTAATTAGCAATAAATATTAATTGAAATTA : 360
Liu_DV4   : -CAATTAACCAATCTTCAAAATACGTTGATTAGGAACTGGTTATTAATAATTACGCATATTTAATTAGCAATAAATATTAATTGAAATTA : 333
             CAATTAACCAATCTTCAAAATACGTTGATTAGGAACTGGTTATTAATAATTACGCATATTTAATTAGCAATAAATATTAATTGAAATTA

                     *       380         *       400         *       420         *       440         *
Guan_DV4  : CATCTAAATAGTTATTCGGTTATGAATGCGTTTATTTAAACTTTAGAAGATATACCAGGCTGTCATCCCGAGGTAGGATTGCCACCCCCA : 429
Zheng_DV4 : CATCTAAATAGTTATTCGGTTATGAATGCGTTTATTTAAACTTTAGAAGATATACCAGGCTGTCATCCCGAGGTAGGATTGCCACCTCCA : 450
Liu_DV4   : CATCTAAATAGTTATTCGGTTATGAATGCGTTTATTTAAACTTTAGAAGATATACCAGGCTGTCATCCCGAGGTAGGATTGCCACCTCCA : 423
            CATCTAAATAGTTATTCGGTTATGAATGCGTTTATTTAAACTTTAGAAGATATACCAGGCTGTCATCCCGAGGTAGGATTGCCACCtCCA

                   460         *       480         *       500         *       520         *       540
Guan_DV4  : TCTGGAGTTACCTGCATTTGTGGAGTAGGAATATCTTTAAAATAATAATAATAATAATAAT---------TTATCATGGCTATCTTATTT : 510
Zheng_DV4 : TCTGGAGTTACCTGCATTTGTGGAGTAGGAATATCTTTAAAATAATAATAATAATAATAAT---------TTATCATGGCTATCTTATTT : 531
Liu_DV4   : TCTGGAGTTACCTGCATTTGTGGAGTAGGAATATCTTTAAAATAATAATAATAATAATAATAATAATAATTTATCATGGCTATCTTATTT : 513
            TCTGGAGTTACCTGCATTTGTGGAGTAGGAATATCTTTAAAATAATAATAATAATAATAAT         TTATCATGGCTATCTTATTT

                     *       560         *       580         *       600         *       620         *
Guan_DV4  : GAAAAGAAATACGTTTGTTTACAAACAATATAAACCCAAGTTTTATTATTGCGTTTATGAAACATGAAAGGTAATCTATTGAAATTTAGA : 600
Zheng_DV4 : GAAAAGAAATACGTTTGTTTACAAACAACATAAACCCAAGTTTTATTATTGCGTTTATGAAACATGAAAGGTAATCTATTGAAATTTAGA : 621
Liu_DV4   : GAAAAGAAATACGTTTGTTTACAAACAATATAAACCCAAGTTTTATTATTGCGTTTATGAAACATGAAAGGTAATCTATTGAAATTTAGA : 603
            GAAAAGAAATACGTTTGTTTACAAACAAtATAAACCCAAGTTTTATTATTGCGTTTATGAAACATGAAAGGTAATCTATTGAAATTTAGA

                   640         *       660         *       680         *       700         *       720
Guan_DV4  : AAAGTGTTTTCTATCCATATATTTTATGGTAGGGAATTCATTGATATTAATATTGATATTATTTTATGTATTATTATTAATATAAATATC : 690
Zheng_DV4 : AAATTGTTTTCTATCCATATATTTTATGGTAGGGAATTCATTGATATTAATATTGATATTATTTTATGTATTATTATTAATATAAATATC : 711
Liu_DV4   : AAAGTGTTTTCTATCCATACATTTTATGGTAGGGAATTCATTGATATTAATATTGATATTATTTTATGTATTATTATTAATATAAATATC : 693
            AAAgTGTTTTCTATCCATAtATTTTATGGTAGGGAATTCATTGATATTAATATTGATATTATTTTATGTATTATTATTAATATAAATATC

                     *       740         *       760         *       780         *       800         *
Guan_DV4  : ATTTTAAAAAATTATATAAATATACATTTGAATAATAAAAAATTATATAAATATAAATTTGAATAATAAAAAATTAAAAGAATTCACAAT : 780
Zheng_DV4 : ATTTTAAAAAATTATATAAATATA-------------------------------AATTTGAATAATAAAAAATTAAAAGAATTCACAAT : 770
Liu_DV4   : ATTTTAAAAAATTATATAAATATACATTTGAATAATAAAAAATTATATAAATATAAATTTGAATAATAAAAAATTAAAAGAATTCACAAT : 783
            ATTTTAAAAAATTATATAAATATAcatttgaataataaaaaattatataaatataAATTTGAATAATAAAAAATTAAAAGAATTCACAAT

                   820         *       840         *       860         *       880         *       900
Guan_DV4  : ATATTAAGTTTTGCTGTTGTTTTT-GTATCCAGAAAACAGAATGTAGTACCAAAGACAGATGCATTATCGCAAGAGGTGATAGTAGAAAC : 869
Zheng_DV4 : ATATTAAGTTTTGCTGTTGTTTTT-GTATCCAGAAAACAGAATGTAGTACCAAAGACAGATGCATTATCGCAAGAGGTGATAGTAGAAAC : 859
Liu_DV4   : ATATTAAGTTTTGCTGTTGTTTTTTGTATCCAGAAAACAGAATGTAGTACCAAAGACAGATGCATTATCGCAAGAGGTGATAGTAGAAAC : 873
            ATATTAAGTTTTGCTGTTGTTTTT GTATCCAGAAAACAGAATGTAGTACCAAAGACAGATGCATTATCGCAAGAGGTGATAGTAGAAAC

                     *
Guan_DV4  : TACTGCGCTTGA : 881
Zheng_DV4 : TACTGCGCTTGA : 871
Liu_DV4   : TACTGCGCTTGA : 885
            TACTGCGCTTGA
```

Figure S5B. Multiple sequence alignments of putative decorsin Hman_DV4 proteins derived from the genome data of *H. manillesis* provided by Guan et al. (2020), Zheng et al. (2023) and Liu et al. (2023), respectively. The cysteine residues are marked in bold and yellow and the RGD motif is marked in cyan and bold. The signal peptide is underlined.

```
                         *        20         *        40         *        60
Guan_DV4   : MLVLIICLLLSFSLDINAKNIPGCHPEVGLPPPSGVTCICGKTECSTKDRCIIARGDSRNYCA- : 63
Zheng_DV4  : MLVLIICLLLSFSLDINAKNIPGCHPEVGLPPPSGVTCICGKTECSTKDRCIIARGDSRNYCA- : 63
Liu_DV4    : MLVLIICLLLSFSLDINAKNIPGCHPEVGLPPPSGVTCICGKTECSTKDRCIIARGDSRNYCA- : 63
             MLVLIICLLLSFSLDINAKNIPGCHPEVGLPPPSGVTCICGKTECSTKDRCIIARGDSRNYCA
```
