# Supplementary material for: Monomeric and Oligomeric Decorsins of the Asian Medicinal Leech Hirudinaria manillensis
Source: Int J Mol Sci. 2025 Nov 14;26(22):11017. doi: 10.3390/ijms262211017 (PMC12651989; doi:10.3390/ijms262211017)
Supplement: Supplementary file 1 [file ijms-26-11017-s001.zip › File S6.pdf]

Figure S6A. Multiple sequence alignments of putative decorsin Hman_DV5 genes derived from the genome data of *H. manillensis* provided by Guan et al. (2020), Zheng et al. (2023) and Liu et al. (2023), respectively. The exons are labeled in green and the introns are labeled in red. Start and stop codons are marked in bold, the cysteine codons are marked in bold and yellow and the RGD motif encoding codons are marked in cyan and bold.

```
                    *        20         *        40         *        60         *        80         *
Guan_DV5  : ATGATAGTGTTGATGATTTGTCTTGCCCTGCCTGTGGGCTTTGCAAGTGGTATGGCATATTATTCAATCGCATGTTAATGCTGATTAATG :   90
Zheng_DV5 : ATGATAGTGTTGATGATTTGTCTTGCCCTGCCTGTGGGCTTTGCAAGTGGTATGGCATATTATTCAATCGCATGTTAATGCTGATTAATG :   90
Liu_DV5   : ATGATAGTGTTGATGATTTGTCTTGCCCTGCCTGTGGGCTTTGCAAGTGGTATGGCATATTATTCAATCGCATGTTAATGCTGATTAATG :   90
            ATGATAGTGTTGATGATTTGTCTTGCCCTGCCTGTGGGCTTTGCAAGTGGTATGGCATATTATTCAATCGCATGTTAATGCTGATTAATG

                   100         *       120         *       140         *       160         *       180
Guan_DV5  : CCGATAATTGTTATTCAATTAGTGTGTTTTTTGTTTTCTATTAAACTTTTGAATGTCAGGAAATTAGAATTTCAGTGTGACATACATTAT :  180
Zheng_DV5 : CCGATAATTGTTATTCAATTAGTGTGTTTTTTGTTTTCTATTAAACTTTTGAATGTCAGGAAATTAGAATTTCAGTGTGACATACATTAT :  180
Liu_DV5   : CCGATAATTGTTATTCAATTAGTGTGTTTTTTGTTTTCTATTAAACTTTTGAATGTCAGGAAATTAGAATTTCAGTGTGACATAC----- :  175
            CCGATAATTGTTATTCAATTAGTGTGTTTTTTGTTTTCTATTAAACTTTTGAATGTCAGGAAATTAGAATTTCAGTGTGACATACattat

                    *       200         *       220         *       240         *       260         *
Guan_DV5  : ATATATAATATATAAATATTAGTTTTGTTACATTACATTTGTTACATTGTTACATTATTAATTCGCGGTTAAGTAGTAACATTATTAGTC :  270
Zheng_DV5 : ATATATAATATATAAATATTAGTTTTGTTACATTACATTTGTTACATTGTTACATTATTAATTCGCGGTTAAGTAGTAACATTATTAGTC :  270
Liu_DV5   : ------------------------------------------------------------------------------------------ :  175
            atatataatatataaatattagttttgttacattacatttgttacattgttacattattaattcgcggttaagtagtaacattattagtc

                   280         *       300         *       320         *       340         *       360
Guan_DV5  : AATGCAGGATCGAAGGATTGCTCAGTCGTGGAGAGAGACTACTTTGGATTTCCAATTGGTCCTTGCATTTGTGCCTGGGTTAGTCACTGA :  360
Zheng_DV5 : AATGCAGGATCGAAGGATTGCTCAGTCGTGGAGAGAGACTACTTTGGATTTCCAATTGGTCCTTGCATATGTGCCTGGGTTAGTCACTGA :  360
Liu_DV5   : ------------------------------------------------------------------------------------------ :  175
            aatgcaggatcgaaggattgctcagtcgtggagagagactactttggatttccaattggtccttgcat tgtgcctgggttagtcactga

                    *       380         *       400         *       420         *       440         *
Guan_DV5  : TCAGTTTTGATAATATATCTGCTCATTAAAAATGTGCTTATTTGTAAATTTATTATCAGTCACGACATTTATATCATTTTGGAGCTGATA :  450
Zheng_DV5 : TCAGTTTTGATAATATGTCTGCTCATTAAAAATGTGCTTATGTGTAAATTTATTATCAGTCACGACATTTATATCATTTTGGAGCTGATA :  450
Liu_DV5   : ------------------------------------------------------------------------------------------ :  175
            tcagttttgataatat tctgctcattaaaaatgtgcttat tgtaaatttattatcagtcacgacatttatatcattttggagctgata

                   460         *       480         *       500         *       520         *       540
Guan_DV5  : AATAATTTAGAAAATACTATGAGGCCATGCCATCATTTATCAAGGGTAAGGAGTGTGGACAAGACGAATATTGTCAAATTGCTAGAGGAG :  540
Zheng_DV5 : AATAATTTAGAAAATACTATGAGGCCATGCCATTATTTATCAAGGGTAAGGAGTGTGGACAAGACGAATATTGTCAAATTGCTAGAGGAG :  540
Liu_DV5   : ------------------------------------------------------------------------------------------ :  175
            aataatttagaaaatactatgaggccatgccat atttatcaagggtaaggagtgtggacaagacgaatattgtcaaattgctagaggag

                    *       560         *       580         *       600         *       620         *
Guan_DV5  : ACAACATCAACTATTGTGTGAAAAGTAAGAACCGGATAAATTACGAAATAAGGAATAATGTCATCATTTAATGTTGTCCTTTTATAATCA :  630
Zheng_DV5 : ACAACATCAACTATTGTGTGAAAAGTAAGAACCGGATAAATTACGGAATAAGGAATAATGTCATCATTTAATGTTGTCCTTTTATAATCA :  630
Liu_DV5   : ------------------------------------------------------------------------------------------ :  175
            acaacatcaactattgtgtgaaaagtaagaaccggataaattacg aataaggaataatgtcatcatttaatgttgtccttttataatca

                   640         *       660         *       680         *       700         *       720
Guan_DV5  : GAATATTGCACTGGTGACAGGGCTAATGATTCAGCAAGGTGCATTTGTGGAGTAAGTGAAACTTTATGTAATATCTATTATTATTATTAT :  720
Zheng_DV5 : GAATATTGCACTGGTGACAGGGCTAATGATTCAGCAAGGTGCATTTGTGGAGTAAGTGAAATTTTATGTAATATCTATTATTATTATTAT :  720
Liu_DV5   : ------------------------------------------------------------------------------------------ :  175
            gaatattgcactggtgacagggctaatgattcagcaaggtgcatttgtggagtaagtgaaa tttatgtaatatctattattattattat

                    *       740         *       760         *       780         *       800         *
Guan_DV5  : ------------------------------GAGAAAATAATGAAATATCTTTTGTATTTCAGGATAAGCGATGCGGGTATAACGAGGACT :  780
Zheng_DV5 : TATTATTATTATTATTATTATTATTATTATGAGAAAATAATGAAATATCTTTTGTATTTCAGGATAAGCGATGCGGGTATAACGAGGACT :  810
Liu_DV5   : ------------------------------------------------------------------------------------------ :  175
                                          gagaaaataatgaaatatcttttgtatttcaggataagcgatgcgggtataacgaggact

                   820         *       840         *       860         *       880         *       900
Guan_DV5  : GCAAGAAAAAATGGAAATAAATTGCAATGTGTCGAATGTCAGTATCATTTAGATGTTTTTATTGTGTTTCAAAATTGTTGCTAACACGATT :  870
Zheng_DV5 : GCAAGAAAAAATGGAAACAAATTGCAATGTGTCGAAAGTCAGTATCATTTAGATGTTTTTATTGTGTTTCAAAATTGTTGCTAACACGATT :  900
Liu_DV5   : ------------------------------------------------------------------------------------------ :  175
            gcaagaaaaaatggaaa aaattgcaatgtgtcgaa gtcagtatcatttagatgtttttattgtgtttcaaaattgttgctaacacgatt

                    *       920         *       940         *       960         *       980         *
Guan_DV5  : GTAAAACAATATGTTATTATATGTACACAAGGATCATATTTCACAGACGAGTCCGTGTCTTTTATTAAATCTCGTTCGTACGGGATAATA :  960
Zheng_DV5 : GTAAAACAATATGTTATTATATGTACACAAGGATCATATTTCACAGACGAGTCCGTGTCTTTTTTTAACTCTCGGTCGTACGGGATAATA :  990
Liu_DV5   : ------------------------------------------------------------------------------------------ :    -
            gtaaaacaatatgttattatatgtacacaaggatcatatttcacagacgagtccgtgtctttt ttaa tctcg tcgtacgggataata

                  1000         *      1020         *      1040         *      1060         *      1080
Guan_DV5  : GCGAGTCGTGATTCTTTCTGTTGACTTTGTGAAAATTAATATAGACTGAAAATTAATTCATTCGATAATTGCTTTGAGCTTTGTATTTAC : 1050
Zheng_DV5 : GCGAGTCGTGATTCTTTCTGTTGACTTTGTGAAAATTAATATAGACTGAAAATTAATTCATTCGATAATTGCTTTGAGCTTTGTATTTAC : 1080
Liu_DV5   : ------------------------------------------------------------------------------------------ :  175
            gcgagtcgtgattctttctgttgactttgtgaaaattaatatagactgaaaattaattcattcgataattgctttgagctttgtatttac

                    *      1100         *      1120         *      1140         *      1160         *
Guan_DV5  : TCATGTACCTTGATCAATCAACGGACGTGAGTGATCTCAACTCGAAGAAAGTTTAAACAAAGTTTAGACATGTTTCTTCCCAATGATAGG : 1140
Zheng_DV5 : TCATGTACCTTGATCAATCAACGGACGTGAGTGATCTCAACTCGAAGAAAGTTTAAACAAAGTTTAGACATGTTTCTTCCCAATGATAGG : 1170
Liu_DV5   : ------------------------------------------------------------------------------------------ :  175
            tcatgtaccttgatcaatcaacggacgtgagtgatctcaactcgaagaaagtttaaacaaagtttagacatgtttcttcccaatgatagg
```

```
                      1180         *      1200         *      1220         *      1240         *      1260
Guan_DV5   : CTTTACAAAAATTTGTATTAGTATAACATTTTTT-AAAATGTTTATTTTGAAAACATTCTCGCAGATGATTTAAGATGCTTTAGGAGCA : 1229
Zheng_DV5  : CTTTACAAAAATTTGTATTAGTATAACATTTTTTTAAAATGTTTATTTTGAAAACATTCTCGCAGATGATTTAAGATGCTTTAGGAGCA : 1260
Liu_DV5    : ------------------------------------------------------------------------------------------ :  175
             ctttacaaaaatttgtattagtataacatttttt aaaatgtttattttgaaaacattctcgcagatgatttaagatgctttaggagca

                    *      1280         *      1300         *      1320         *      1340         *
Guan_DV5   : TGTCTTTTAGAAAATGTGAAACACGTCACATGGACTGTCGATTTTTATCATTTTATTTACCAAACATTTCCTTATAAATTCTTTACTAAC : 1319
Zheng_DV5  : TGTCTTTTAGAAAATGTGAAACACGTCACATGGACTGTCGATTTTTATCATTTTATTTACCAAACATTTCCTTATAAATTCTTTACTAAC : 1350
Liu_DV5    : ------------------------------------------------------------------------------------------ :  175
             tgtcttttagaaaatgtgaaacacgtcacatggactgtcgatttttatcattttatttaccaaacatttccttataaattctttactaac

                      1360         *      1380         *      1400         *      1420         *      1440
Guan_DV5   : TGGTGTACGAATTCTTTGGATGTCGCAGGAAGACATCATTGATTTATTTCAGAATGGACAGGTGGGAAAGGTGGCTAAAGTTTTAATAAC : 1409
Zheng_DV5  : TGGTGTACGAATTCTTTGGATGTCGCAGGAAGACATCATTGATTTATTTCAGAATGGACAGGTGGGAAAGGTGGCTAAAGTTTTAATAAC : 1440
Liu_DV5    : ------------------------------------------------------------------------------------------ :  175
             tggtgtacgaattctttggatgtcgcaggaagacatcattgatttatttcagaatggacaggtgggaaaggtggctaaagttttaataac

                    *      1460         *      1480         *      1500         *      1520         *
Guan_DV5   : ATATTAGATTTTACTGAAAGCTACAAGATATCTAGGTATAAAGATATGCTAATTTGTTATGGTCGAAAAGATGAAATTTTGACAAAATAC : 1499
Zheng_DV5  : ATATTAGATTTTACTGAAAGCTACAAGATATCTAGGTATAAAGATATGCTAATTTGTTATGGTCGAAAAGATGAAATTTTGACAAAATAC : 1530
Liu_DV5    : ------------------------------------------------------------------------------------------ :  175
             atattagattttactgaaagctacaagatatctaggtataaagatatgctaatttgttatggtcgaaaagatgaaattttgacaaaatac

                      1540         *      1560         *      1580         *      1600         *      1620
Guan_DV5   : ACATAAATAGATAAAGTTGAGATCACATAACATTAATATTAATTGCAGAACCTCAGCATTGTTCACATGTGGAGAAAGATGAATTCGGAA : 1589
Zheng_DV5  : ACATAAATAGATAAAGTTGAGATCACATAACATTAATATTAATTGCAGAACCTCAGCATTGTTCACATGTGGAGAAAGATGAATTCGGAA : 1620
Liu_DV5    : ------------------------------------------------------------------------------------------ :  175
             acataaatagataaagttgagatcacataacattaatattaattgcagaacctcagcattgttcacatgtggagaaagatgaattcggaa

                    *      1640         *      1660         *      1680         *      1700         *
Guan_DV5   : TACCCATTGGTCCTTGTATTTGTTATGTTAGTCTTGATCAAGTACTTTACTCGTTAAAAACGTGCTTGTTTGTAATTTGTCAATTTGTTA : 1679
Zheng_DV5  : TACCCATTGGTCCTTGTATTTGTTATGTTAGTCTTGATCAAGTACTTTACTCGTTAAAAACGTGCTTGTT-GTAATTTGTCAATTTGTTA : 1709
Liu_DV5    : ------------------------------------------------------------------------------------------ :  175
             tacccattggtccttgtatttgttatgttagtcttgatcaagtactttactcgttaaaaacgtgcttgtt gtaatttgtcaatttgtta

                      1720         *      1740         *      1760         *      1780         *      1800
Guan_DV5   : ACATTCATGACATTTGTATAACTGTAAAGTTTAATTAATTAAAAGGAGGCGCTTCGTTCTTTATCCGCTCATCCATCCATCCATCCATCC : 1769
Zheng_DV5  : ACATTCATGACATTTGTATAACTGTAAAGTTTAATTAATTAAAAGGAGGCGCTTCGTTCTTTATCCGCTCATCCAACCAT---------- : 1789
Liu_DV5    : ------------------------------------------------------------------------------------------ :  175
             acattcatgacatttgtataactgtaaagtttaattaattaaaaggaggcgcttcgttctttatccgctcatcca ccat

                    *      1820         *      1840         *      1860         *      1880         *
Guan_DV5   : ATCCATTAATTCATCTACGCATTCATCCATCTTATCCTTCCTTCCATCTATACATCCATTCAACCAACCAACCACCCATCCACCTACCTA : 1859
Zheng_DV5  : ------TAATTCATCTACGCATTCATCCATCTTATCCTTCCTTCCATCTATACATCCATTCAACCAACCAACCACCCATCCACCTACCTA : 1873
Liu_DV5    : ------------------------------------------------------------------------------------------ :  175
                   taattcatctacgcattcatccatcttatccttccttccatctatacatccattcaaccaaccaaccacccatccacctaccta

                      1900         *      1920         *      1940         *      1960         *      1980
Guan_DV5   : GTAAACCAATTTACACAATGGTTTACAAAACATTGTGTTGAAATCGTACTATTATTTACCAAGCATCTCGAGTGTGAAGAGAACGAATAT : 1949
Zheng_DV5  : GTAAACCAATTTACACAATGGTTTACAAAACATTGTGTTGAAATCGTACTATTATTTACCAAGCATCTCGAGTGTGAAGAGAACGAATAT : 1963
Liu_DV5    : -----------------------------------------------------ATTTACCAAGTATCTCGAGTGTGAAGAGAACGAATAT :  212
             gtaaaccaatttacacaatggtttacaaaacattgtgttgaaatcgtactattATTTACCAAGcATCTCGAGTGTGAAGAGAACGAATAT

                    *      2000         *      2020         *      2040         *      2060         *
Guan_DV5   : TGTAATGTTACCGGAGGAATGTACTTCAGCTTTTGTGAACGATGTAAGTTCCAAGTAAATTTCGAAATAAACATCATTTAATGAATAATT : 2039
Zheng_DV5  : TGTAATGTTACCGGAGGAATGTACTTCAGCTTTTGTGAACGATGTAAGTTCCAAGTAAATTTCGAAATAAACATCATTTAATGAATAATT : 2053
Liu_DV5    : TGTAATGTTACCGGAGGAATGTACTTCAGCTTTTGCGAACGATGTAAGTTCCAAGTAAATTTCGAAATAAACATCATTTAATGAATAATT :  302
             TGTAATGTTACCGGAGGAATGTACTTCAGCTTTTGtGAACGATGTAAGTTCCAAGTAAATTTCGAAATAAACATCATTTAATGAATAATT

                      2080         *      2100         *      2120         *      2140         *      2160
Guan_DV5   : TTCACATTTATTCCTCCGCTAATATTTAATATAATTAAGTGAATATTTAATTTAATGCAATTATTCAATTTTCGTAAAAAATAGAGAAAT : 2129
Zheng_DV5  : TTCACATTTATTCCTCCGCTAATATTTAATATAATTAAGTGAATATTTAATTTAATGCAATTATTCAATTTTCGTAAAAAATAGAGAAAT : 2143
Liu_DV5    : TTCACATTTATTCCTCCGCTAATATTTAATATAATTAAGTGAATATTTAATTTAATGCAATTATTCAATTTTCGTAAAAAATAGAGAAAT :  392
             TTCACATTTATTCCTCCGCTAATATTTAATATAATTAAGTGAATATTTAATTTAATGCAATTATTCAATTTTCGTAAAAAATAGAGAAAT

                    *      2180         *      2200         *      2220         *      2240         *
Guan_DV5   : AACTTAAATGTAAATGTCAGATTAAAATGATTAACCTTAAAACACAAACGTTTTCAAATCAGTGTATTGTACGGGTAACCCGGCTGAAGA : 2219
Zheng_DV5  : AACTTAAATGTAAATGTCAGATTAAAATGATTAACCTTAAAACACAAACGTTTTCAAATCAGTGTATTGTACGGGTAACCCGGCTGAAGA : 2233
Liu_DV5    : AACTTAAATTTAAATGTCAGATTAAAATGATTAACCTTAAAACACAAACGTTTTCAAATCAGTGTATTGTGCGGGTAACCCGGCTGAAGA :  482
             AACTTAAATgTAAATGTCAGATTAAAATGATTAACCTTAAAACACAAACGTTTTCAAATCAGTGTATTGTaCGGGTAACCCGGCTGAAGA

                      2260         *      2280         *      2300         *      2320         *      2340
Guan_DV5   : TTCTCCATTGTGCATATGTAAAGTCAGTAACATTTTATATAACACTAATAATTACTGTTATAAATTATACAATTCTAAATTAATAATAAT : 2309
Zheng_DV5  : TTCTCCATTGTGCATATGTAAAGTCAGTAACATTTTATCTAACACTAATAATTACTGTTATAAATTATACAATTCTAAATTAATAATAAT : 2323
Liu_DV5    : TTCTCCATTGTGCATATGTAAAGTCAGTAACATTTTATCTAATACTAATAATTACTGTTATAAATTATACAATTCTAAATTAATAATAAT :  572
             TTCTCCATTGTGCATATGTAAAGTCAGTAACATTTTATcTAAcACTAATAATTACTGTTATAAATTATACAATTCTAAATTAATAATAAT

                    *      2360         *      2380         *      2400         *      2420         *
Guan_DV5   : ATTTATGAGGCTAATTATGACGAAAAAATGAAGAAATATCTTTTTGTCTTACAGGATAAGCGATGCAAGCATAATCAATACTGCAGGAAC : 2399
Zheng_DV5  : ATTTATAAGGATAATTATGACGAAAAAATGAAGAAATATCTTTT-GTCTTGCAGGATAAGCGATGCAAGCATAATCAATACTGCAGGAAC : 2412
Liu_DV5    : ATTTATAAGGATAATTATGACGAAAAAATGAAGAAATATCTTTTTGTCTTGCAGGATAAGCGATGCGACCATAATCAATACTGCAGGAAC :  662
             ATTTATaAGGaTAATTATGACGAAAAAATGAAGAAATATCTTTTtGTCTTgCAGGATAAGCGATGCaAgCATAATCAATACTGCAGGAAC
```

```
                  2440         *      2460         *      2480         *      2500         *      2520
Guan_DV5    :  TTTAATGGCAAGATGCAATGTACTAAAAGTGAGTTTCTCTTAAGTGTTTTTTATTGTGAGGAACATTATTTTAAGGTTGTTAATATCATT  : 2489
Zheng_DV5   :  TTTAATGGCAAGATGCAATGTACTAAAAGTGAGTTTCTCTTA-GTGTTTTTTATTGTGAGGAACATTATTTTAAGGTTGTTAATATCATT  : 2501
Liu_DV5     :  TTTAATGGCAAGATGCAATGTACTAAAAGTGAGTTTCTCTTAGCTGTTTTTTATTGTGAGGAACATTATTTTAAGGTTGTTAATATCATT  :  752
               TTTAATGGCAAGATGCAATGTACTAAAAGTGAGTTTCTCTTA gTGTTTTTTATTGTGAGGAACATTATTTTAAGGTTGTTAATATCATT

                        *      2540         *      2560         *      2580         *      2600         *
Guan_DV5    :  GATAGCTTTAAAATAATATATATTTCTATGTATTGTAATAATAATAAATATCATATGTAGGTATTAATAATAATAACAATAATATTATAA  : 2579
Zheng_DV5   :  GATAGCTTTAAAATAATATATATTTCTATGTATTGTAATAATAATAAATATCATATGTAAGTATTAATAATAATAACAATAATATTATAA  : 2591
Liu_DV5     :  GATAGCTTTAAAATAATATATATTTCTATGTATTGTAATAATAATAAATATCATATGTAGGTATTAATAATAATAACAATAATATTATAA  :  842
               GATAGCTTTAAAATAATATATATTTCTATGTATTGTAATAATAATAAATATCATATGTAgGTATTAATAATAATAACAATAATATTATAA

                  2620         *      2640         *      2660         *      2680         *      2700
Guan_DV5    :  TAACAATAATGCTAATAATAATGATGATGTATTTTATCAAGCGCATTAATAGAAGGTATGACTAATGCTCTAATATGTTATGTAAAATTA  : 2669
Zheng_DV5   :  TAACAATAATGCTAATAATAATGATGATGTATTTTATCAAGCGGATTAATAGAAGGTATGACTAATGCTCTA---TGTTATGTAAAATTA  : 2678
Liu_DV5     :  TAACAATGATGCTAATAATAATGATGATGTATTTTATCAAGCGGATTAATAGAAGGTATGACTAATGCTCTA---TGTTATGTAAAATTA  :  929
               TAACAATaATGCTAATAATAATGATGATGTATTTTATCAAGCGgATTAATAGAAGGTATGACTAATGCTCTA   TGTTATGTAAAATTA

                        *      2720         *      2740         *      2760         *      2780         *
Guan_DV5    :  AGTTCATATTGCTATTAGATTATCAGTTTAGTCAAACCGATGTTGTACAGCCATGTTAATTTTTTAGCCTGCTAACAGTTAATATGCTGT  : 2759
Zheng_DV5   :  AGTTCATATCGCTATTAGATTATCAGTTTAGTCAAACCGATGTTGTACAACCATGTTAATTTTTTAGCCTGCTAACAGTTAATATGCTGT  : 2768
Liu_DV5     :  AGTTCACATCGCTATTAGATTATCAGTTTAGTCAAACCGATGTTGTACAACCATGTTAATTTTTTAGCCTGCTAACAGTTAATATGCTGT  : 1019
               AGTTCAtATcGCTATTAGATTATCAGTTTAGTCAAACCGATGTTGTACAaCCATGTTAATTTTTTAGCCTGCTAACAGTTAATATGCTGT

                  2800         *      2820         *      2840         *      2860         *      2880
Guan_DV5    :  TAAGTATTATTATGTTATTATTATTAAATGCAGGACCGGAGAGTTGCGAAAATGTGCGAAAGGACAAACTCAATCGCCCCATTAGTCCTT  : 2849
Zheng_DV5   :  TAAGTATTATTATGTTATTATTATTAAATGCAGGACCGGAGAGTTGCGAAAATGTGCGAAAGGACAAACTCAATCGCCCCATTAGTCCTT  : 2858
Liu_DV5     :  TAAGTATTATTATGTTATTATTATTAAATGCAGGACCGGAGAGTTGCGAAAATGTGCGAAAGGACAAACTCAATCGCCCCATTAGTCCTT  : 1109
               TAAGTATTATTATGTTATTATTATTAAATGCAGGACCGGAGAGTTGCGAAAATGTGCGAAAGGACAAACTCAATCGCCCCATTAGTCCTT

                        *      2900         *      2920         *      2940         *      2960         *
Guan_DV5    :  GCGTTTGTGGAATGGTTAGTCGTCGATAAATTTTGGTCATTTTTAAAATTGTACTCAAATTTTAATTAATTTCTCAATTTATTATAATTT  : 2939
Zheng_DV5   :  GCGTTTGTGGAATGGTTAGTCGTTGATAAATTT-GGTCATTTTTAAAATTGTACTCAAATTTTAATTAATTTCTCAATTTATTATAATTT  : 2947
Liu_DV5     :  GCGTTTGTGGAATGGTTAGTCGTCGATAAATTTTGGTCATTTTTAAAATTGTACTCAAATTTTAATTAATTTCTCAATTTATTATAATTT  : 1199
               GCGTTTGTGGAATGGTTAGTCGTcGATAAATTTtGGTCATTTTTAAAATTGTACTCAAATTTTAATTAATTTCTCAATTTATTATAATTT

                  2980         *      3000         *      3020         *      3040         *      3060
Guan_DV5    :  TAAAGGCTGTAAAGTTCAGAATTGGTTTAGAAGATATTTATGATTACACAATCATTATTTACCAAGGGTCGGGTTTGTGAAAAGCACGAA  : 3029
Zheng_DV5   :  TAAACGCTGTAAAGTTCAGAATTGGTTTAGAAGATATTTATGATTTCACAATCATTATTTACCAAGGGTCGGGTTTGTGAAAAGCACGAA  : 3037
Liu_DV5     :  TAAACGCTGTAAAGTTCAGAATTGGTTTAGAAGATATTTATGATTTCACAATCATTATTTACCAAGGGTCGGGTTTGTGAAAAGCACGAA  : 1289
               TAAAcGCTGTAAAGTTCAGAATTGGTTTAGAAGATATTTATGATTtCACAATCATTATTTACCAAGGGTCGGGTTTGTGAAAAGCACGAA

                        *      3080         *      3100         *      3120         *      3140         *
Guan_DV5    :  TACTGTAATATGACACATGAAGATGCAACCAACGTTTGCTTACCAAGTACGTTTCGTTTAGATTATGAAATAAACAATTACATAAAGATT  : 3119
Zheng_DV5   :  TACTGTAATATGACACATGAAGATGCAACCAACGTTTGCTTACCAAGTACGTTTCGTTTAGATTATGAAATAAACAATTACATAAAGATT  : 3127
Liu_DV5     :  TACTGTAATATGACACATGAAGATGCAACCAACGTTTGCTTACCAAGTACGTTTCGTTTAGATTATGAAATAAACAATTACATAAAGATT  : 1379
               TACTGTAATATGACACATGAAGATGCAACCAACGTTTGCTTACCAAGTACGTTTCGTTTAGATTATGAAATAAACAATTACATAAAGATT

                  3160         *      3180         *      3200         *      3220         *      3240
Guan_DV5    :  TATGGATTAATTTTCACATTGATTCATATTAACGCTTTGATTTTACTTAGAATATCTCCCGTGCAAACTGGGACCAATTGTTCAGAAGAA  : 3209
Zheng_DV5   :  TATGGATTAATTTTCACATTGATTCATATTAACGCTTTGATTTTACCTAGAATATCTCCCGTGCAAACTGGGACCAATTGTTCAGAAGAA  : 3217
Liu_DV5     :  TATGGATTAATTTTCACATTGATTCATATTAACGCTTTGATTTTACCTAGAATATCTCCCGTGCAAACTGGGACCAATTGTTCAGAAGAA  : 1469
               TATGGATTAATTTTCACATTGATTCATATTAACGCTTTGATTTTACcTAGAATATCTCCCGTGCAAACTGGGACCAATTGTTCAGAAGAA

                        *      3260         *      3280         *      3300         *      3320         *
Guan_DV5    :  ATGTTTATGTGGTGTTAGTAACTATTACTGACAGACATTCTTTTGTATTTCAGATTAAGAGATATTTATTACATTAAAGTACCTAAAAAT  : 3299
Zheng_DV5   :  ATGTTTATGTGGTGTTAGTAATTATTACTGACAGACATTCTTTTGTATTTCAGATTAAGAGATATTTATTACATTAAAGTACCTAAAAAT  : 3307
Liu_DV5     :  ATGTTTATGTGGTGTTAGTAACTATTACTGACAGACATTCTTTTGTATTTCAGATTAAGAGATATTTATTACATTCAAGTACCTATAAAT  : 1559
               ATGTTTATGTGGTGTTAGTAAcTATTACTGACAGACATTCTTTTGTATTTCAGATTAAGAGATATTTATTACATTaAAGTACCTAaAAAT

                  3340         *      3360         *      3380         *      3400         *      3420
Guan_DV5    :  AACATTAAAATAAAGTTAGCCATATGTTATACATGTAGAATAAGTCGTGCAACAAAGGAAAGAAATGCAAATCGATTAAAGGCGAAGCAA  : 3389
Zheng_DV5   :  AACATTAAAATAAAGTTAGCCATATGTTATACATGTAGAATAAGTCGTGCAACAAAGGAAAGAAATGCAAATCGATTAAAGGCGAAGCAA  : 3397
Liu_DV5     :  AACATTAAAATAAAGTTATCCATATCTTATACATGTAGAATAAGTCGTGCAACAAAGGAAAGAAATGCAAATCGATTAAAGGCGAAGCAA  : 1649
               AACATTAAAATAAAGTTAgCCATATgTTATACATGTAGAATAAGTCGTGCAACAAAGGAAAGAAATGCAAATCGATTAAAGGCGAAGCAA

                        *      3440         *      3460         *      3480         *      3500         *
Guan_DV5    :  AATGCTTGAAAGGTTGTATACACATCTTCGTTTCTACATTGATATTGTTGAAATATGAATTTGAGTTTTGGAATGATTATCACTATTAAT  : 3479
Zheng_DV5   :  AATGCTTGAAAGGTTGTATACACATCTTCGTTTCTACATTGATATTGTTGAAATATGAATTAGAGTTTTGGATTGATTATCACTATTAAT  : 3487
Liu_DV5     :  AATGCTTGAAAGGTTGTATGCACATCTTCGTTTCTACATTGATATTGTTGAAATATGAATTTGAGTTTTGGAATGATTATCACTATTAAT  : 1739
               AATGCTTGAAAGGTTGTATaCACATCTTCGTTTCTACATTGATATTGTTGAAATATGAATTtGAGTTTTGGAaTGATTATCACTATTAAT

                  3520         *      3540         *      3560         *      3580         *      3600
Guan_DV5    :  AATGCTACTAATATTATTAGATTAATATTATTATTAATTCTATTATTTTCAGTTAGCAAAAAGAAGAAGATATACAAAAATGCCGAATAA  : 3569
Zheng_DV5   :  AATGCTACTAATATTATTAGATTAATATTATTATTAATTCTATTATTTTCAGTTAGCAAAAAGAAGAAGATATACAAAAATGCCGAATAA  : 3577
Liu_DV5     :  AATGCTACTAATATTATTAGATTAATATTATTATTAATTCTATTATTTTCAGTTAGCAAAAAGAAGAAGATATACAAAAATGCCGAATAA  : 1829
               AATGCTACTAATATTATTAGATTAATATTATTATTAATTCTATTATTTTCAGTTAGCAAAAAGAAGAAGATATACAAAAATGCCGAATAA
```

Figure S6B. Multiple sequence alignments of putative decorsin Hman_DV5 proteins derived from the genome data of *H. manillesis* provided by Guan et al. (2020), Zheng et al. (2023) and Liu et al. (2023), respectively. The cysteine residues are marked in bold and yellow and the RGD motif is marked in cyan and bold. The signal peptide is underlined.

```
                          *        20         *        40         *        60         *        80         *
Guan_DV5   : MIVLMICLALPVGFASGSKDCSVVERDYFGFPIGPCICAWGKECGQDEYCQIARGDNINYCVKKYCTGDRANDSARCICGDKRCGYNEDC :  90
Zheng_DV5  : MIVLMICLALPVGFASGSKDCSVVERDYFGFPIGPCICAWGKECGQDEYCQIARGDNINYCVKKYCTGDRANDSARCICGDKRCGYNEDC :  90
Liu_DV5    : MIVLMICLALPVGFAS-------------------------------------------------------------------------- :  16
             MIVLMICLALPVGFASgskdcsvverdyfgfpigpcicawgkecgqdeycqiargdninycvkkyctgdrandsarcicgdkrcgynedc

                    100         *       120         *       140         *       160         *       180
Guan_DV5   : KKNGNKLQCVE-PQHCSHVEKDEFGIPIGPCICYHLECEENEYCNVTGGMYFSFCERLYCTGNPAEDSPLCICKDKRCKHNQYCRNFNGK : 179
Zheng_DV5  : KKNGNKLQCVEKPQHCSHVEKDEFGIPIGPCICYHLECEENEYCNVTGGMYFSFCERLYCTGNPAEDSPLCICKDKRCKHNQYCRNFNGK : 180
Liu_DV5    : ----------------------------------YLECEENEYCNVTGGMYFSFCERLYCAGNPAEDSPLCICKDKRCDHNQYCRNFNGK :  72
             kkngnklqcve pqhcshvekdefgipigpcicyhLECEENEYCNVTGGMYFSFCERLYCtGNPAEDSPLCICKDKRCkHNQYCRNFNGK

                      *       200         *       220         *       240         *       260         *
Guan_DV5   : MQCTKRPESCENVRKDKLNRPISPCVCGMGRVCEKHEYCNMTHEDATNVCLPKYLPCKLGPIVQKKCLCGNKSCNKGKKCKSIKGEAKCL : 269
Zheng_DV5  : MQCTKRPESCENVRKDKLNRPISPCVCGMGRVCEKHEYCNMTHEDATNVCLPKYLPCKLGPIVQKKCLCGNKSCNKGKKCKSIKGEAKCL : 270
Liu_DV5    : MQCTKRPESCENVRKDKLNRPISPCVCGMGRVCEKHEYCNMTHEDATNVCLPKYLPCKLGPIVQKKCLCGNKSCNKGKKCKSIKGEAKCL : 162
             MQCTKRPESCENVRKDKLNRPISPCVCGMGRVCEKHEYCNMTHEDATNVCLPKYLPCKLGPIVQKKCLCGNKSCNKGKKCKSIKGEAKCL

                    280
Guan_DV5   : KVSKKKKIYKNAE- : 282
Zheng_DV5  : KVSKKKKIYKNAE- : 283
Liu_DV5    : KVSKKKKIYKNAE- : 175
             KVSKKKKIYKNAE
```
