# Supplementary material for: Monomeric and Oligomeric Decorsins of the Asian Medicinal Leech Hirudinaria manillensis
Source: Int J Mol Sci. 2025 Nov 14;26(22):11017. doi: 10.3390/ijms262211017 (PMC12651989; doi:10.3390/ijms262211017)
Supplement: Supplementary file 1 [file ijms-26-11017-s001.zip › Table S1.pdf]

Table S1. List of oligonucleotide primers used in the study.

Hman_DV1_fw:	5`-AAT AGA TCA CAG CCA **TGT** GAA TTA G-3`<br>Tm: 63 °C
Hman_DV1_rev:	5`-TT*C TGC AGT TA*C AAC CTT GT**G CA**A TTT GC-3`<br>Tm: 71 °C
Hman_DV1k_rev:	5`-TT*A AGC* ***TTA*** AAT GAT GAG ATT ATT CTT-3`<br>Tm: 58 °C
Hman_DV3s-1a_fw:	5`-GAT TTA CAG TCT **TGT** CGC TCG-3`<br>Tm: 64 °C
Hman_DV3s-1a_rev:	5`-GC*A AGC* ***TTA*** GGA ATT AGT AAT ACC CTG TCT **GCA** GTA ATC-3`<br>Tm: 72 °C
Hman_DV3s-4a_fw:	5`-AGA CCA GAG GAT **TGC** ACA GTC G-3`<br>Tm: 70 °C
Hman_DV3s-4a_rev:	5`-GC*A AGC* ***TTA*** AAA CTT ACT TCC TAA **GCA** TTG GTT G-3`<br>Tm: 70 °C
Hman_DV4_fw:	5`-AAG AAT ATA CCA GGC **TGT** CAT CC-3`<br>Tm: 65 °C
Hman_DV4_rev:	5`-TT*A AGC TT*C AAG CGC AGT AGT TTC TAC-3`<br>Tm: 67 °C

standard PCR mix:

5x Q5 DNA polymerase reaction buffer	:	5.0 µl
forward primer	:	2.5 µl to a final concentration of 1 µmol/l
reverse primer	:	2.5 µl to a final concentration of 1 µmol/l
dNTP mix (10 mmol/l of each dNTP)	:	0.5 µl
synthetic gene construct (0.1 µg/µl)	:	0.5 µl
Q5 DNA polymerase	:	0.5 µl
aqua bidest (molecular biology grade)	:	13.5 µl

expected lengths of amplicons:	DV1:	780 bp
	DV1k:	198 bp
	DV3s-1a:	162 bp

DV3s-4a:	159 bp
DV4:	145 bp
