# Supplementary material for: Monomeric and Oligomeric Decorsins of the Asian Medicinal Leech Hirudinaria manillensis
Source: Int J Mol Sci. 2025 Nov 14;26(22):11017. doi: 10.3390/ijms262211017 (PMC12651989; doi:10.3390/ijms262211017)
Supplement: Supplementary file 1 [file ijms-26-11017-s001.zip › Table S2.pdf]

Table S2. Combinations of globular domains that can be deduced from the Hman_DV3 gene of *H. manillensis* by regular and alternative splicing using the exon skipping mechanism. "1" - "6" are the repeats of the globular domain, "a" indicates the first and "b" the second codon that encode the globular domain. Exons that contain an RGD motif are marked in bold and cyan. The total numbers of combinations are labeled in bold and yellow, the numbers in brackets indicate factors that contain two, one or no globular domain, respectively.

**Hexameric factors**	**Pentameric factors**	**Tetrameric factors**	**Trimeric factors**	**Dimeric factors**	Monomeric factors
1a**1b**2a2b3a**3b**4a4b5a5b6a6b	1a**1b**2a2b3a**3b**4a4b5a5b	1a**1b**2a2b3a**3b**4a4b	1a**1b**2a2b3a**3b**	1a**1b**2a2b	1a**1b**
	1a**1b**2a2b3a**3b**4a4b5a6b	1a**1b**2a2b3a**3b**4a5b	1a**1b**2a2b3a4b	1a**1b**2a3b	1a2b
	1a**1b**2a2b3a**3b**4a4b6a6b	1a**1b**2a2b3a**3b**4a6b	1a**1b**2a2b3a5b	1a**1b**2a4b	1a**3b**
	1a**1b**2a2b3a**3b**4a5b6a6b	1a**1b**2a2b3a**3b**5a5b	1a**1b**2a2b3a6b	1a**1b**2a5b	1a4b
	1a**1b**2a2b3a**3b**5a5b6a6b	1a**1b**2a2b3a**3b**5a6b	1a**1b**2a2b4a4b	1a**1b**2a6b	1a5b
	1a**1b**2a2b3a4b5a5b6a6b	1a**1b**2a2b3a**3b**6a6b	1a**1b**2a2b4a5b	1a**1b**3a**3b**	1a6b
	1a**1b**2a2b4a4b5a5b6a6b	1a**1b**2a2b4a4b5a5b	1a**1b**2a2b4a6b	1a**1b**3a4b	2a2b
	1a**1b**2a**3b**4a4b5a5b6a6b	1a**1b**2a2b4a4b5a6b	1a**1b**2a2b5a5b	1a**1b**3a5b	2a**3b**
	1a**1b**3a**3b**4a4b5a5b6a6b	1a**1b**2a2b4a4b6a6b	1a**1b**2a2b5a6b	1a**1b**3a6b	2a4b
	1a2b3a**3b**4a4b5a5b6a6b	1a**1b**2a2b5a5b6a6b	1a**1b**2a2b6a6b	1a**1b**4a4b	2a5b
	2a2b3a**3b**4a4b5a5b6a6b	1a**1b**3a**3b**4a4b5a5b	1a**1b**3a**3b**4a4b	1a**1b**4a5b	2a6b
		1a**1b**3a**3b**4a4b5a6b	1a**1b**3a**3b**4a5b	1a**1b**4a6b	3a**3b**
		1a**1b**3a**3b**4a4b6a6b	1a**1b**3a**3b**4a6b	1a**1b**5a5b	3a4b
		1a**1b**3a**3b**4a5b6a6b	1a**1b**3a**3b**5a5b	1a**1b**5a6b	3a5b
		1a**1b**3a**3b**5a5b6a6b	1a**1b**3a**3b**5a6b	1a**1b**6a6b	3a6b
		1a2b3a**3b**4a4b5a5b	1a**1b**3a**3b**6a6b	1a2b3a**3b**	4a4b
		1a2b3a**3b**4a4b5a6b	1a**1b**4a4b5a5b	1a2b3a4b	4a5b
		1a2b3a**3b**4a4b6a6b	1a**1b**4a4b5a6b	1a2b3a5b	4a6b
		1a2b3a**3b**4a5b6a6b	1a**1b**4a4b6a6b	1a2b3a6b	5a5b
		1a2b3a**3b**5a5b6a6b	1a**1b**4a5b6a6b	1a2b4a4b	5a6b
		1a2b3a4b5a5b6a6b	1a**1b**5a5b6a6b	1a2b4a5b	6a6b
		1a2b4a4b5a5b6a6b	1a2b3a**3b**4a4b	1a2b4a6b	
		1a**3b**4a4b5a5b6a6b	1a2b3a**3b**4a5b	1a2b5a5b	
		2a2b3a**3b**4a4b5a5b	1a2b3a**3b**4a6b	1a2b5a6b	
		2a2b3a**3b**4a4b5a6b	1a2b3a**3b**5a6b	1a2b6a6b	
		2a2b3a**3b**4a4b6a6b	1a2b3a**3b**6a6b	1a**3b**4a4b	
		2a2b3a**3b**4a5b6a6b	1a2b4a4b5a5b	1a**3b**4a5b	

		2a2b3a**3b**5a5b6a6b	1a2b4a4b5a6b	1a**3b**4a6b	
		2a2b3a4b5a5b6a6b	1a2b4a4b6a6b	1a**3b**5a5b	
		2a2b4a4b5a5b6a6b	1a2b4a5b6a6b	1a**3b**5a6b	
		2a**3b** 4a4b5a5b6a6b	1a2b5a5b6a6b	1a**3b**6a6b	
		3a**3b** 4a4b5a5b6a6b	1a**3b**4a4b5a5b	1a4b5a5b	
			1a**3b**4a4b5a6b	1a4b5a6b	
			1a**3b**4a4b6a6b	1a4b6a6b	
			1a**3b**4a5b6a6b	1a5b6a6b	
			1a**3b**5a5b6a6b	2a2b3a**3b**	
			1a4b5a5b6a6b	2a2b3a4b	
			2a2b3a**3b**4a4b	2a2b3a5b	
			2a2b3a**3b**4a5b	2a2b3a6b	
			2a2b3a**3b**4a6b	2a2b4a4b	
			2a2b3a**3b**5a5b	2a2b4a5b	
			2a2b3a**3b**5a6b	2a2b4a6b	
			2a2b3a**3b**6a6b	2a2b5a5b	
			2a2b4a4b5a5b	2a2b5a6b	
			2a2b4a4b5a6b	2a2b6a6b	
			2a2b4a4b6a6b	2a**3b**4a4b	
			2a2b4a5b6a6b	2a**3b**4a5b	
			2a2b5a5b6a6b	2a**3b**4a6b	
			2a**3b**4a4b5a5b	2a**3b**5a5b	
			2a**3b**4a4b5a6b	2a**3b**5a6b	
			2a**3b**4a4b6a6b	2a**3b**6a6b	
			2a**3b**4a5b6a6b	2a4b5a5b	
			2a**3b**5a5b6a6b	2a4b5a6b	
			2a4b5a5b6a6b	2a4b6a6b	
			3a**3b**4a4b5a5b	2a5b6a6b	
			3a**3b**4a4b5a6b	3a**3b**4a4b	
			3a**3b**4a4b6a6b	3a**3b**4a5b	
			3a**3b**4a5b6a6b	3a**3b**4a6b	
			3a**3b**5a5b6a6b	3a**3b**5a5b	
			3a4b5a5b6a6b	3a**3b**5a6b	

			4a4b5a5b6a6b	3a**3b**6a6b<br>3a4b5a5b<br>3a4b5a6b<br>3a4b6a6b<br>3a5b6a6b<br>4a4b5a5b<br>4a4b5a6b<br>4a4b6a6b<br>4a5b6a6b<br>5a5b6a6b	
**1** (1-0-0)	**11** (7-4-0)	**32** (11-17-4)	**61** (7-40-14)	**70** (1-34-35)	**21** (0-4-17)

total: **196** (27-99-70)
